# A K^+^-promoted Diels–Alder reaction by using a self-assembled macrocyclic boronic ester containing two crown ether moieties†
†Electronic supplementary information (ESI) available. CCDC 1541693. For ESI and crystallographic data in CIF or other electronic format see DOI: 10.1039/c9sc01597c


**DOI:** 10.1039/c9sc01597c

**Published:** 2019-07-03

**Authors:** Kosuke Ono, Morikazu Niibe, Nobuharu Iwasawa

**Affiliations:** a Department of Chemistry , Tokyo Institute of Technology , O-okayama, Meguro-ku , Tokyo 152-8551 , Japan . Email: niwasawa@chem.titech.ac.jp

## Abstract

A K^+^-promoted Diels–Alder reaction of 1,4,9,10-anthradiquinone with various dienes is achieved in the presence of a self-assembled macrocyclic boronic ester **[2+2]_crown_** containing two crown ether moieties. The reaction rate is remarkably accelerated (up to 206-fold) compared to that in the absence of the promoter. Furthermore, the reaction proceeds regioselectively to yield an internal adduct. The self-assembly protocol was also demonstrated.

## Abstract 2

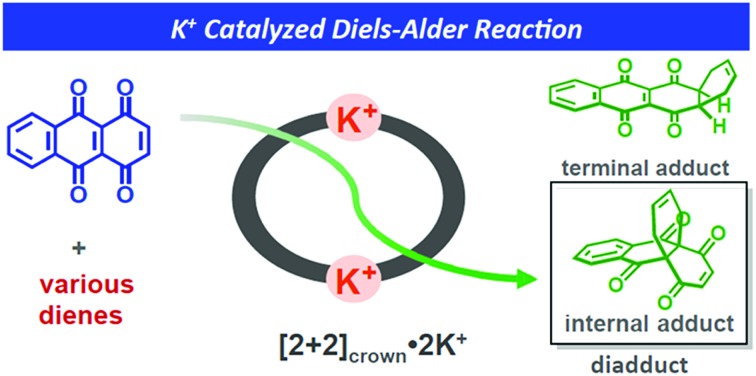
A K^+^-promoted Diels–Alder reaction of 1,4,9,10-anthradiquinone with various dienes is achieved in the presence of a self-assembled macrocyclic boronic ester.

## Introduction

One of the major goals for utilization of synthetic host molecules is their application as catalysts.^1^ Among various organic reactions, the Diels–Alder reaction, which is one of the most useful reactions for the construction of 6-membered carbocycles, has been studied using synthetic host molecules, such as hydrogen-bonded capsules,^2^ cyclic metalloporphyrin trimers,^3^ coordination cages,^4^ and so on.^5^ Through these studies, reaction characteristics such as acceleration of the reaction rate,^1^–^5^ formation of products with unique regio- or stereo-selectivity,4b–d catalytic turnover,2c,d,4b,h or asymmetric induction4*f* were demonstrated depending on the host molecules. These reaction characteristics were mostly achieved by a strategy in which both a diene and a dienophile were pre-organized inside the host molecules. However, this strategy often led to a problem known as product inhibition, because the host usually binds the product more strongly than the substrates.^1^–^5^ To solve this problem, we devised a new type of supramolecular catalyst using a macrocyclic boronic ester containing two crown ether moieties. The macrocyclic host binds and activates the dienophile through M^+^-crown ether moieties and promotes the Diels–Alder reaction with various dienes. After the reaction, the bent-shaped product that showed weaker binding affinity could be replaced by the dienophile easily through the open framework. In this strategy, the entropic disadvantage arising from the need to bind both substrates is also reduced. Thus, the exchange process of the product with the dienophile is thought to be energetically neutral. Recently, the group of Lusby introduced this simple dienophile-binding strategy and realized the efficient catalytic turnover by using the coordination cage that activated the dienophile by hydrogen bonding.4h,^6^

**Fig. 1:**
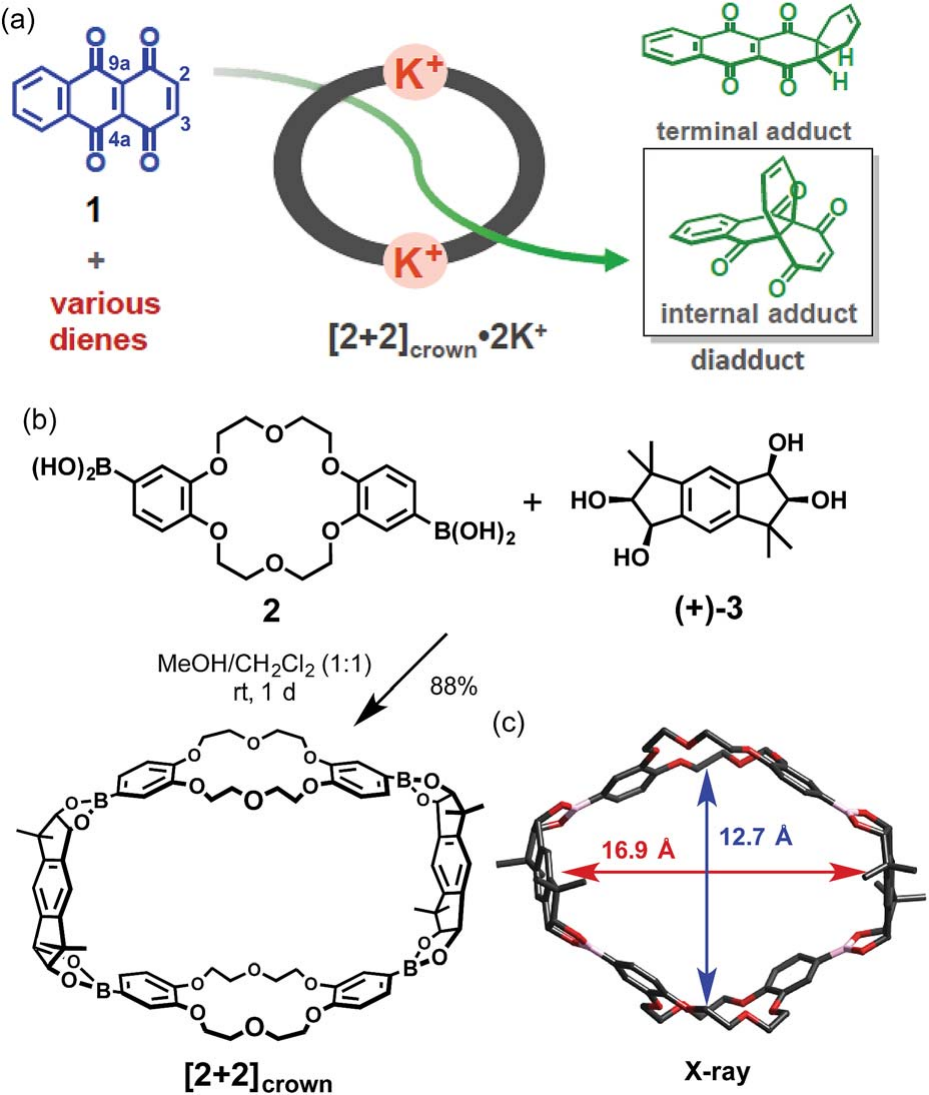
(a) Schematic representation of the Diels–Alder reaction of 1,4,9,10-anthradiquinone **1** and various dienes accelerated using **[2+2]_crown_·2K^+^**. (b) Self-assembly of **[2+2]_crown_**. (c) X-ray structure of **[2+2]_crown_**.

Research is being carried out on dynamic self-assembly utilizing boronic ester formation of diboronic acids with an indacene-type tetrol.^7^ During the examination of the possibility of utilizing our hosts as catalysts, we observed a concise self-assembly of the macrocyclic boronic ester **[2+2]_crown_**, containing two dibenzo-18-crown-6 moieties, and a hitherto unknown K^+^-accelerated Diels–Alder reaction, which showed not only acceleration of the reaction rate but also enhancement of the internal regioselectivity in the reaction of 1,4,9,10-anthradiquinone and various dienes (Fig. 1).

## Results and discussion

The macrocyclic boronic ester **[2+2]_crown_** was quite easily prepared in high yield by simply mixing a diboronic acid of dibenzo-18-crown-6 **2** and optically pure tetrol **(+)-3** (ref. 8) in MeOH–CH_2_Cl_2_ (1 : 1), followed by GPC purification (Fig. 1b).^9^,^10^ Formation of the desired **[2+2]_crown_** was fully confirmed by ^1^H NMR and FAB-MS, and the structure was confirmed by X-ray crystallographic analysis (Fig. 1c), which suggested that its cavity size (16.9 × 12.7 Å) was suitable for inclusion of quinone type compounds (Fig. 1c).^11^

**Fig. 2:**
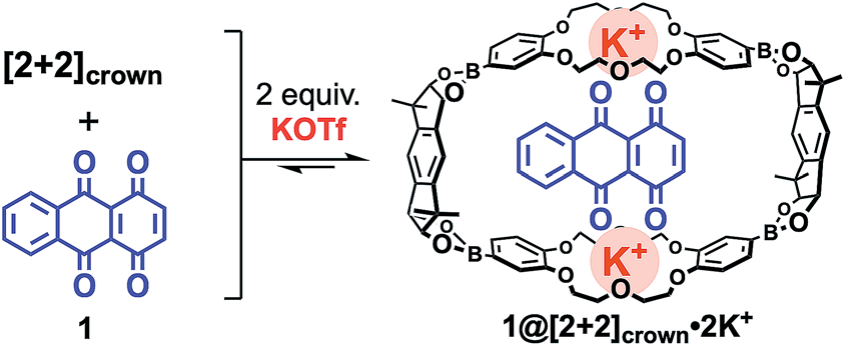
Complexation of **[2+2]_crown_** with diquinone **1** in the presence of K^+^.

With the desired macrocyclic boronic ester **[2+2]_crown_** in hand, the binding behavior of **[2+2]_crown_** containing alkaline metal salts with several quinonoid compounds was examined. From ^1^H NMR and isothermal titration calorimetry (ITC) studies, 1 : 1 complexation behavior was revealed for 1,4,9,10-anthradiquinone **1** and **[2+2]_crown_·2K^+^** with a high association constant (*K*_a_ = 3.54 × 10^3^ M^–1^; CHCl_3_/CH_3_CN (1 : 1))^12^ (Fig. 2)^9^,^13^ (see the ESI†). It should be noted that the use of other metal cations (Li^+^ or Na^+^) instead of K^+^ or a mixture of monomeric pinacol ester of dibenzo-18-crown-6 diboronic acid **2_pin_**,^14^ KOTf, and **1** did not show an obvious shift of the signals of **1** by ^1^H NMR. These results suggested that the macrocyclic structure of **[2+2]_crown_** with K^+^ ions was essential for the efficient binding of **1**.^9^,^13^

As it is known that the Diels–Alder reaction of **1** with dienes occurs at both the C-2 and C-4a double bonds giving an internal and a terminal regioisomeric adduct and sometimes a diadduct,^15^ the reaction of **1** with anthracene **4** in the presence or absence of **[2+2]_crown_** and K^+^ was examined with the expectation that high regioselectivity owing to the restriction of the host framework would be achieved.^16^ When a mixture of **1** and **4** was kept at room temperature in a mixed solution of CHCl_3_ and CH_3_CN (1 : 1) for 1.5 hours, the Diels–Alder reaction proceeded very slowly (2% conversion) and a mixture of the internal adduct and terminal adduct was obtained in about a 4 : 1 ratio (Table 1, entry 1). Addition of **[2+2]_crown_** without K^+^ did not affect the result of the reaction (Table 1, entry 2). On the other hand, when the same reaction was carried out in the presence of a stoichiometric amount of **[2+2]_crown_** and 2 equivalents of KOTf, the reaction was dramatically accelerated to give the product in 95% yield under the same reaction conditions, and the rate constant *k* was 206 times larger than that of the reaction without **[2+2]_crown_** and KOTf. Importantly, only the adduct with the internal alkene was obtained selectively (entry 3). Control experiments were carried out using monomeric **2_pin_** with KOTf, and almost no accelerating effect was observed (entry 4). Thus, the macrocyclic host structure of **[2+2]_crown_** was essential for the acceleration. Potassium cations were much more effective than sodium cations, which only had a small accelerating effect (entry 3 *vs.* 5).

**Table 1:** Examination of the Diels–Alder reaction of **1** and **4**^*a*^

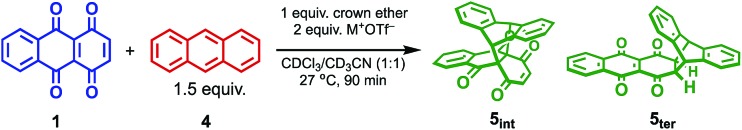						
Entry	Crown ether	**M^+^OTf^–^**	Conversion^*b*^ at 90 min	**5_int_** : **5_ter_**^*b*^	*k* ^*c*^ (M^–1^ min^–1^)	*k*/*k*_no cat_^*c*^
1	None	None	2%	4 : 1	0.033	—
2	**[2+2]_crown_**	None	2%	4 : 1	0.030	0.9
3	**[2+2]_crown_**	KOTf	95%	**5_int_** only	6.87	206
4^*d*^	**2_pin_**	KOTf	2%	4 : 1	0.038	1.1
5	**[2+2]_crown_**	NaTOf	5%	13 : 1	0.175	5.2

^*a*^Reaction conditions: **1** (6.5 mM), **4** (9.8 mM), **M^+^OTf^–^** (13 mM), **[2+2]_crown_** (6.5 mM).

^*b*^Conversion at 1.5 h and ratio of **5_int_**/**5_ter_** were determined by ^1^H NMR.

^*c*^Reaction rate *k* was estimated by using a second-order kinetic model. The value of *k*_no cat_ is taken from the reaction in the absence of crown ether and **M^+^OTf^–^**.

^*d*^ **2_pin_** (13 mM).

It is well known that Lewis acids accelerate the Diels–Alder reaction by coordinating with the lone pair of the carbonyl group resulting in lowering of the LUMO level of the dienophile.^17^ TiCl_4_, SnCl_4_, BF_3_·OEt_2_, *etc.* are employed as typical Lewis acids, and the use of alkaline metals for the acceleration of the Diels–Alder reaction has been mostly limited to the reaction using LiClO_4_ in ether.^18^ In fact, Na^+^ or K^+^ has not been employed for this purpose due to their very low ability to accept electron pairs in the vacant orbital of the metal to activate dienophiles.^19^,^20^ To our knowledge, this is the first example that the Diels–Adler reaction was effectively promoted by potassium ions^19^ and this unique effect was specific to the combination of **[2+2]_crown_** and potassium ions.

**Scheme 1:**
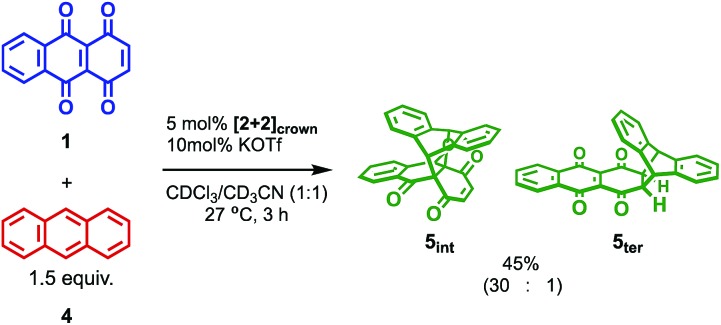
Catalytic conditions of the Diels–Alder reaction.

**Fig. 3:**
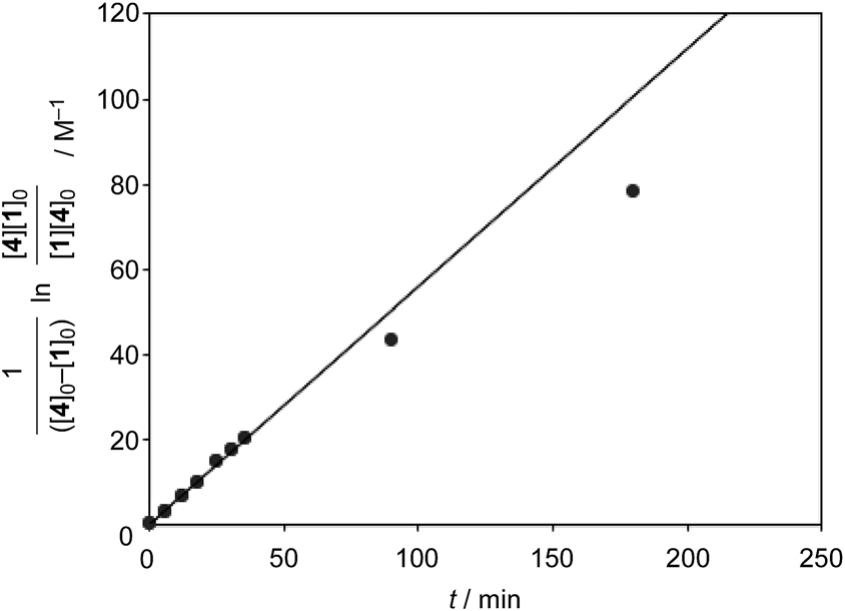
Second-order plot (1/([**4**]_0_ – [**1**]_0_)ln([**4**][**1**]_0_/[**1**][**4**]_0_)/M^–1^*vs.* t/min) for the catalytic conditions of the Diels–Alder reaction. [**4**]_0_ = initial concentration of **4**, [**1**]_0_ = initial concentration of **1**.

The catalytic version of this reaction was also examined, and even 5 mol% of **[2+2]_crown_·2K^+^** was sufficient to promote the reaction (Scheme 1). After 3 hours, 45% of **1** was transformed into the Diels–Alder adduct **5** (**5_int_**/**5_ter_** = 30 : 1), while only 5% conversion was observed without the catalyst. From the second-order plot, after 90 minutes, the reaction rate became slightly slower than that in the initial period, suggesting moderate product inhibition (Fig. 3).^21^ However, reasonable catalytic activity was maintained under the conditions even where the amount of the product was considerably larger than that of the catalyst.

The acceleration of the reaction was observed with various 2-mono and 2,3-di-substituted 1,3-butadienes (Table 2). When dienes with small substituents **6–8** were used, the reactions were remarkably accelerated and the reaction rate constants *k*_cat_ were approximately 20–50 times larger than those of the reaction without **[2+2]_crown_·2K^+^** (entry 1–6). On the other hand, the reactions with dienes with bulky substituents **9–12** were less accelerated (*k*_cat_/*k*_no cat_ ∼ 10) as shown in entries 7–14. Steric repulsion between the **[2+2]_crown_** framework and the bulky substituent has made it difficult to promote the reaction smoothly. However, in all cases, enhancement of the regioselectivity of the product was observed in the presence of **[2+2]_crown_·2K^+^**. In particular, in the case of diene **12** (entry 13 and 14), the ratio of the internal adduct/terminal adduct was dramatically increased from 1 : 1 to 45 : 1 by the addition of **[2+2]_crown_·2K^+^**. Furthermore, chirality induction was observed when 2-mono-substituted 1,3-dienes were used (see the ESI, Table S1†). This also suggested that the **[2+2]_crown_** framework recognized the substituent of dienes and the reaction proceeded inside the host, although the enantioselectivities were low (up to 19% ee). 1-Mono- and 1,4-di-substituted 1,3-butadienes were not applicable to this reaction probably because these dienes could not approach the quinone inside **[2+2]_crown_·2K^+^** due to the steric hindrance of the **[2+2]_crown_** framework (see the ESI, Table S2†).

**Table 2:** Diels–Alder reaction of **1** and various 2-mono and 2,3-di-substituted 1,3-butadienes in the presence or absence of **[2+2]_crown_·2K^+^**^*a*^

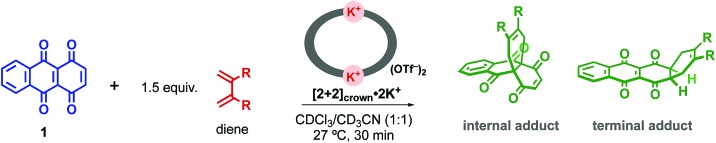						
Entry	Diene	**[2+2]_crown_·2K^+^**	*k* ^*d*^ (M^–1^ min^–1^)	*k*_cat_/*k*_no cat_^*e*^	Internal : terminal^*f*^	Conversion^*f*^ at 30 min
1^*b*^	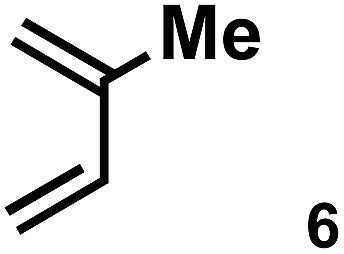	1.0 equiv.	1.43	19	19 : 1	60%
2^*b*^		None	0.077	—	1.6 : 1	8%
3	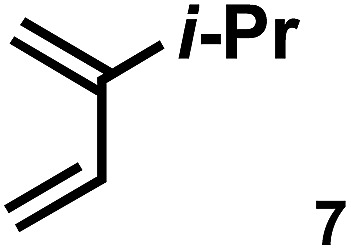	1.0 equiv.	10.90	51	Internal only	84%
4		None	0.21	—	2.4 : 1	11%
5	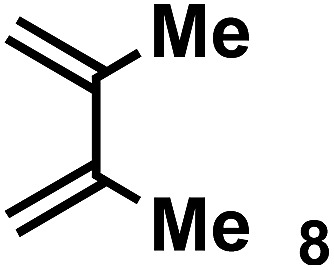	1.0 equiv.	18.27	43	Internal only	93%
6		None	0.43	—	9 : 1	21%
7	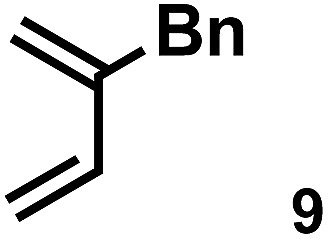	1.0 equiv.	0.74	9.1	10 : 1	13% (40% at 90 min)
8^*c*^		None	0.081	—	1.5 : 1	4% (13% at 90 min)
9	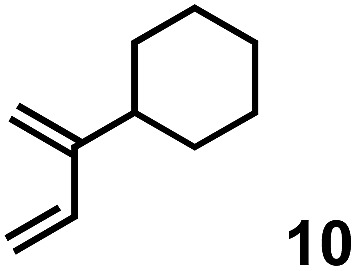	1.0 equiv.	4.64	10	13 : 1	60%
10		None	0.46	—	2 : 1	25%
11	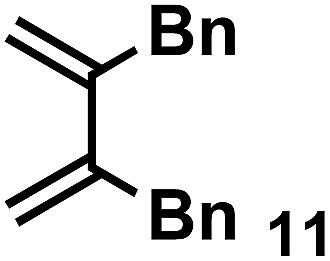	1.0 equiv.	0.64	8.1	Terminal trace	15% (37% at 90 min)
12		None	0.078	—	Terminal trace	7% (17% at 90 min)
13	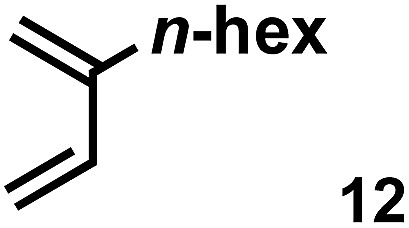	1.0 equiv.	3.30	11	45 : 1	51%
14		None	0.32	—	1 : 1	38%

^*a*^Reaction conditions: **1** (6.5 mM) in the presence of the catalyst; **1** (15 mM) in the absence of the catalyst.

^*b*^3 equiv. of diene were used.

^*c*^ **1** (13 mM).

^*d*^reaction rate *k* was estimated by using a second-order kinetic model with the assumption that **1** is completely complexed with **[2+2]_crown_·2K^+^**, although the estimated value of complexation based on the association constant is about 80% in the beginning.

^*e*^The value of *k*_cat_ or *k*_no cat_ is taken from the reaction in the presence or absence of the catalyst.

^*f*^Ratio of internal adduct/terminal adduct and conversion were determined by ^1^H NMR.

The reason why anthracene **4** shows excellent activity compared to the other dienes **6–12** was also investigated. In the reaction of anthracene (Table 1, entry 3), the signal of the anthracene shifted slightly up-field compared to that of free anthracene in ^1^H NMR spectra, suggesting the formation of a weak Michaelis complex **1**·**4**@**[2+2]_crown_·2K^+^** (Fig. S10†), while no obvious shift was observed without **[2+2]_crown_·2K^+^** (Fig. S11†).^22^ The formation of a substrate pair inside the host was thought to contribute to the acceleration of the reaction of anthracene as a result of the high local concentration of substrates. Subsequently, the possibility of whether the stabilization of the transition state (TS) was involved in the high activity of anthracene was also investigated. The adducts were used as TS analogues and their association constants with the host **[2+2]_crown_·2K^+^** were measured by ITC study.4*h* Interestingly, the association constant of the anthracene adduct **5_int_** was 1.08 × 10^3^ M^–1^ in CHCl_3_/CH_3_CN (1 : 1) (Fig. S12†),^21^ while the association constants of other internal adducts derived from acyclic dienes such as **6**, **9** and **11** that show lower activity were almost 0 M^–1^ (Fig. S25†). These results suggested that the TS stabilization effect of **[2+2]_crown_·2K^+^** could also contribute to the high activity of anthracene.

**Scheme 2:**
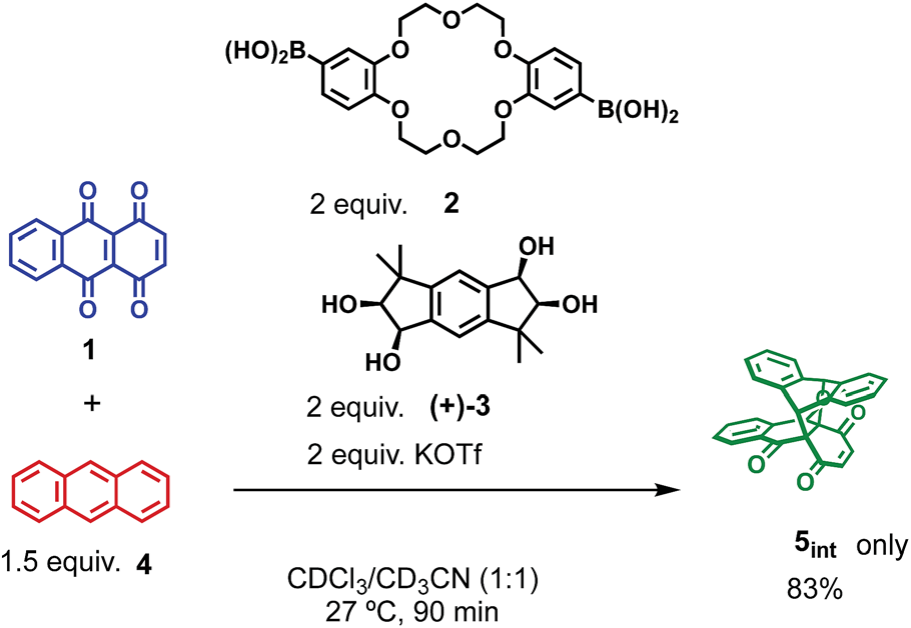
Self-assembly protocol for the Diels–Alder reaction of **1** and **4**.

**Fig. 4:**
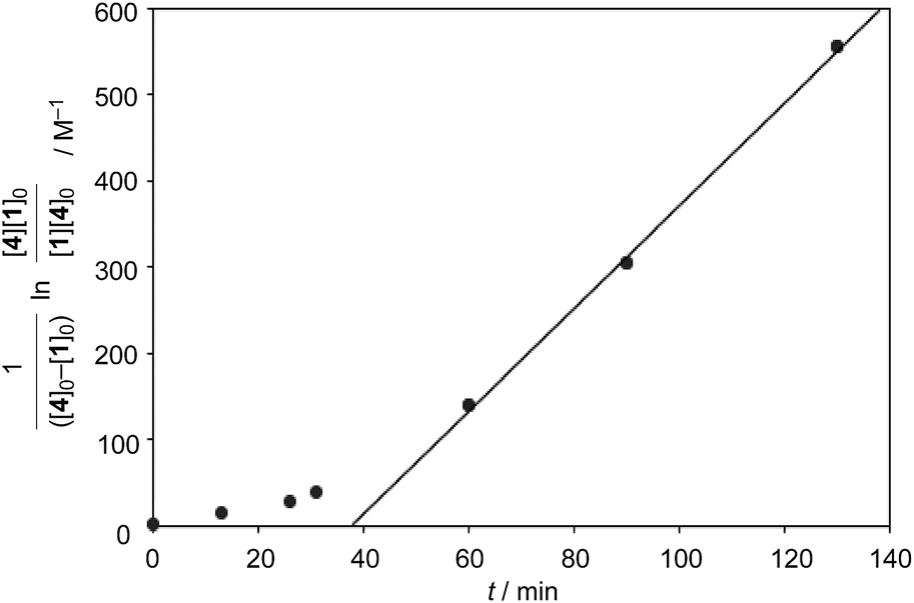
Second-order plot (**1**/([**4**]_0_ – [**1**]_0_)ln([**4**][**1**]_0_/[**1**][**4**]_0_)/M^–1^*vs.* t/min) for the self-assembled promoter system, where all components were mixed at the same time. [**4**]_0_ = initial concentration of **4**, [**1**]_0_ = initial concentration of **1**.

Finally, we examined the possibility of the self-assembly protocol for this reaction. All the components, **1**, **2**, **(+)-3**, and **4**, were mixed together and the process of the formation of the Diels–Alder adduct was monitored by ^1^H NMR (Scheme 2). The internal adduct **5_int_** was obtained as the exclusive product in 83% yield after 90 min. From the second-order plot shown in Fig. 4, the gradual increase of the reaction rate was observed and the reaction rate reached 5.95 M^–1^ min^–1^ from 60 min later. This value is comparable with that obtained by the reaction using preformed **[2+2]_crown_·2K^+^** (6.88 M^–1^ min^–1^, Table 1 entry 2). Thus, the self-assembly of the active **[2+2]_crown_·2K^+^** occurred rapidly in the reaction mixture simply by mixing the component molecules to accelerate the reaction.

## Conclusions

The present study shows that the macrocyclic boronic ester **[2+2]_crown_·2K^+^** efficiently promotes the Diels–Alder reactions of 1,4,9,10-anthradiquinone and various dienes with high regioselectivity. It is noteworthy that four-point binding of the carbonyl groups with potassium cations in the **[2+2]_crown_** framework effectively accelerated the Diels–Alder reaction. Furthermore, the self-assembly protocol was successfully demonstrated by utilizing the dynamic nature of boronic ester linkages, offering the possibility of a novel catalytic system combined with the reversibility of boronic ester formation.

## Conflicts of interest

There are no conflicts to declare.

## Supplementary Material

Supplementary information

Crystal structure data
