# Supplementary material for: A K+-promoted Diels–Alder reaction by using a self-assembled macrocyclic boronic ester containing two crown ether moieties
Source: Chem Sci. 2019 Jul 3;10(32):7627–32. doi: 10.1039/c9sc01597c (PMC6761878; doi:10.1039/c9sc01597c)
Supplement: Supplementary file 1 [file SC-010-C9SC01597C-s001.pdf]

Electronic Supplementary Material (ESI) for Chemical Science.
This journal is © The Royal Society of Chemistry 2019

K^+ Catalyzed Diels-Alder Reaction by Macrocyclic Bis(crown ether) Boronic Ester

Kosuke Ono, Morikazu Niibe and Nobuharu Iwasawa*,†

Department of Chemistry, Tokyo Institute of Technology, O-okayama, Meguro-ku, Tokyo 152-8551, Japan

*e-mail: niwasawa@chem.titech.ac.jp

Table of Contents

1. General Methods ………………………………………………………………….. S2
2. Self-assembly of **[2+2]crown**………….…………………………………..………….. S3
3. Complexation of **[2+2]crown** with 1,4,9,10-Anthradiquinone **1** in the Presence of 2 equiv. KOTf..………………………………………………………………………….. S5
3-1. NMR Study of the Complexation of **[2+2]crown** with **1** in the Presence of 2 equiv. of KOTf……………………………...……………………………...……… S5
3-2. ITC Study of the Complexation of **[2+2]crown** with **1** in the Presence of 2 equiv. of KOTf……………………………………………………………………………….. S5
3-3. Control Experiments of the Complexation of **[2+2]crown** with **1**…………………… S6
4. The Diels-Alder Reaction of **1** with Various Dienes ………………………………… S8
4-1. The Diels-Alder Reaction of **1** with Anthracene **4** Under Various Conditions…………………….. …………………………………………………... S8
4-2-1. The Diels-Alder Reaction of **1** with 2-Mono and 2,3-Di-substituted 1,3-Butadienes……………………………………………………………………….. S15
4-2-2. The Enantioselective Diels-Alder Reaction of **1** with 2-Substituted 1,3-Butadienes……………………………………………………………………….. S41
4-3. The Diels-Alder Reaction of **1** with 1-Mono and 1,4-Di-substituted 1,3-Butadienes... ……………………………………………………………………….. S43
5. Experimental Procedure and Characterization of New Compounds…………………….. S49
6. ^{1}H and ^{13}C NMR Spectra of New Compounds……….………………………… S53
7. X-ray Crystallographic Analysis of a Racemic Crystal of **[2+2]crown**……….…….. S59
8. References…………………………………………………………….……… S62

1. General Methods

All operations were performed under air unless otherwise noted. ^{1}H and ^{13}C NMR spectra were recorded on a JEOL ECX-500, a Bruker DRX-500 (500 MHz for ^{1}H and 125 MHz for ^{13}C), or a JEOL ECX-400, a JEOL AL-400, a JEOL Lambda-400 (400 MHz for ^{1}H and 100 MHz for ^{13}C), or a JEOL AL-300 (300 MHz for ^{1}H and 75 MHz for ^{13}C) spectrometer using $CDCl_3$ [residual $CHCl_3$ (7.26 ppm) served as an internal standard in ^{1}H NMR and $CDCl_3$ (77.0 ppm) in ^{13}C NMR], dimethylsulfoxide (DMSO)-d_6 [residual DMSO (2.49 ppm) served as an internal standard in ^{1}H NMR and DMSO-d_6 (39.73 ppm) in ^{13}C NMR] as a solvent. Chemical shifts are expressed in parts per million (ppm). High- or low-resolution mass analyses (FAB^{+}) were performed on a JEOL JMS-700 mass spectrometer. High- or low-resolution mass analyses (FD^{+}) were performed on a JEOL T-GCV mass spectrometer. IR spectra were recorded on a JASCO FT/IR-460 plus spectrometer. Gel permeation chromatography (GPC) was performed using LC-9130NEXT series (Japan Analytical Industry Co., Ltd.). ITC (Isothermal Titration Calorimetry) experiments were performed on a Microcal iTC200 at 298 K. GPC (Gel Permeation Chromatography) experiments were performed on a JAIGEL-1H and JAIGEL-2H columns. The single crystal X-ray diffraction data were collected on a Rigaku R-AXIS II with IP area detector system using Cu$K\alpha$ radiation (α = 1.54186 Å). Dehydrated dichloromethane, DMF, Et_2O, acetonitrile, toluene and benzene was purchased from Kanto Chemical Co., Inc. and all other solvents were distilled before using. Tetrahydrofuran (THF) was purified by solvent purification system of Glass-Contour. Other solvents were distilled according to the usual procedures and stored over molecular sieves. 1,4,9,10-Anthradiquinone **1**,[S1] enantiopure tetrol (+)-**3** and (–)-**3**,[S2] bis[2-(methylsulfonyl)oxyethyl]ether,[S3] 2-substituted-1,3-butadiene (**7**, **9**, **10**, **12**)[S4] were prepared according to the literature procedures.

2. Self-assembly of [2+2]$_{crown}$

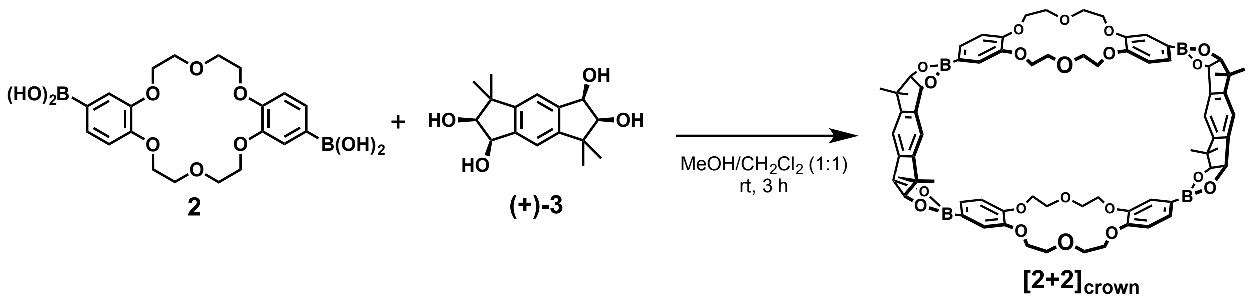

The (+)-tetrol **(+)-3** (17.7 mg, 0.064 mmol) was added to a methanol/ CH_2Cl_2 (1:1) solution (3.6 mL) of **2** (30 mg, 0.067 mmol). The reaction mixture became homogeneous in a few minutes. After the mixture was stirred at room temperature for 24 hours, solvent was removed under reduced pressure. The residue was purified by GPC to afford the desired product **[2+2]$_{crown}$** (37 mg, 88 %).

Physical data of **[2+2]$_{crown}$**

^{1}H NMR (500 MHz, $CDCl_3$): δ 7.34-7.32 (m, 8H), 7.17 (s, 4H), 6.75 (d, J = 8.8 Hz, 4H), 5.83 (d, J = 6.2 Hz, 4H), 4.80 (d, J = 6.2 Hz, 4H), 4.11 (br, 16H), 3.98 (br, 16H), 1.44 (s, 12H), 1.26 (s, 12H).

^{13}C NMR (125 MHz, $CDCl_3$): δ 151.3, 150.1, 147.8, 141.3, 129.1, 120.4, 119.9, 118.3. 111.7, 90.3, 83.0, 69.8, 69.7, 68.4, 67.9, 46.7, 31.5, 23.0.

HRMS (FAB$^+$, NBA): m/z Calcd. for $C_{72}H_{80}O_{20}B_4K$: 1346.5289., Found: 1346.5299 $[M]^+$.

IR (ATR): 1601, 1518, 1419, 1356, 1310, 1260, 1213, 1142, 1093, 1055, 953 cm^{-1}.

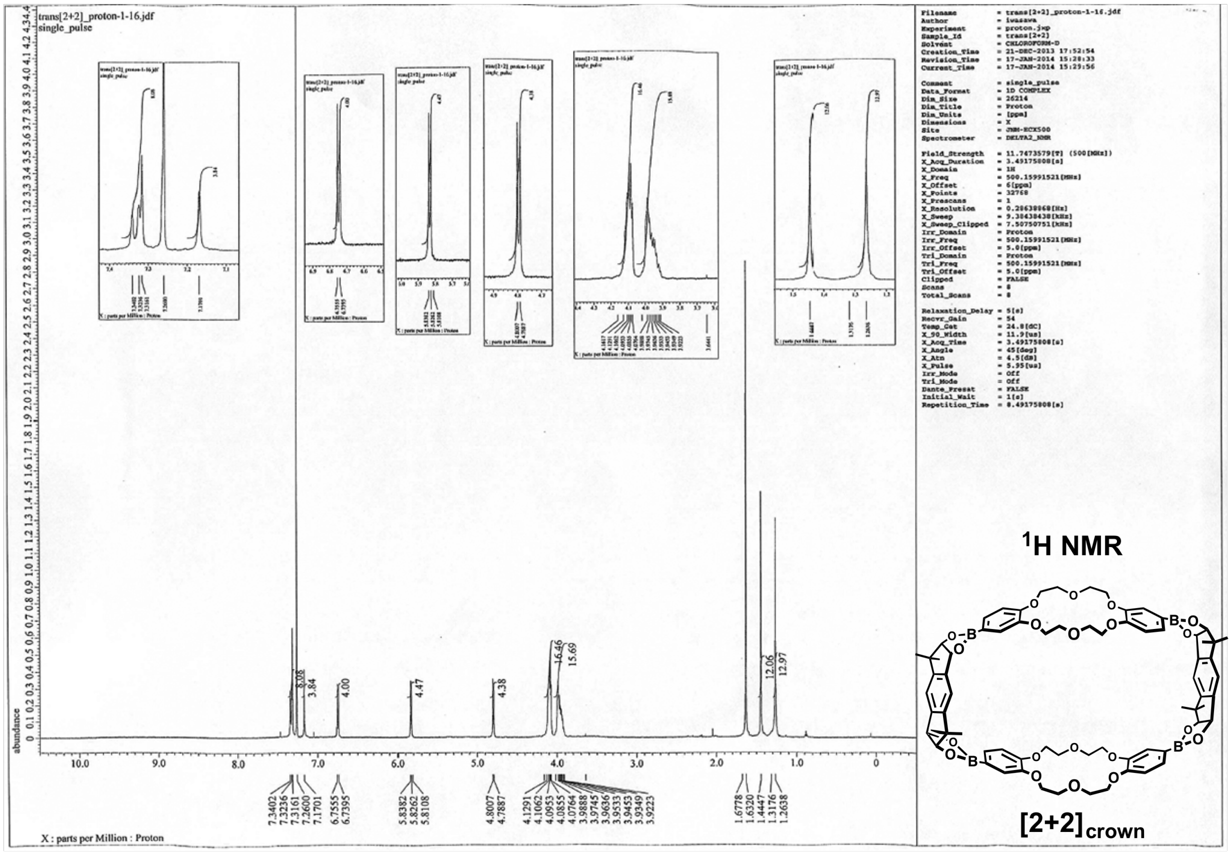
trans[2+2]_proton-1-16.jdf
single_pulse
8.08
3.84
4.00
4.47
4.38
16.46
15.69
12.06
12.97
7.3402
7.3236
7.3161
7.2600
7.1701
6.7555
6.7395
5.8382
5.8262
5.8108
4.8007
4.7887
4.1291
4.1062
4.0953
4.0855
4.0764
3.9888
3.9745
3.9636
3.9533
3.9453
3.9349
3.9223
1.6778
1.6320
1.4447
1.3176
1.2638
X : parts per Million : Proton
abundance
1H NMR
[2+2]crown

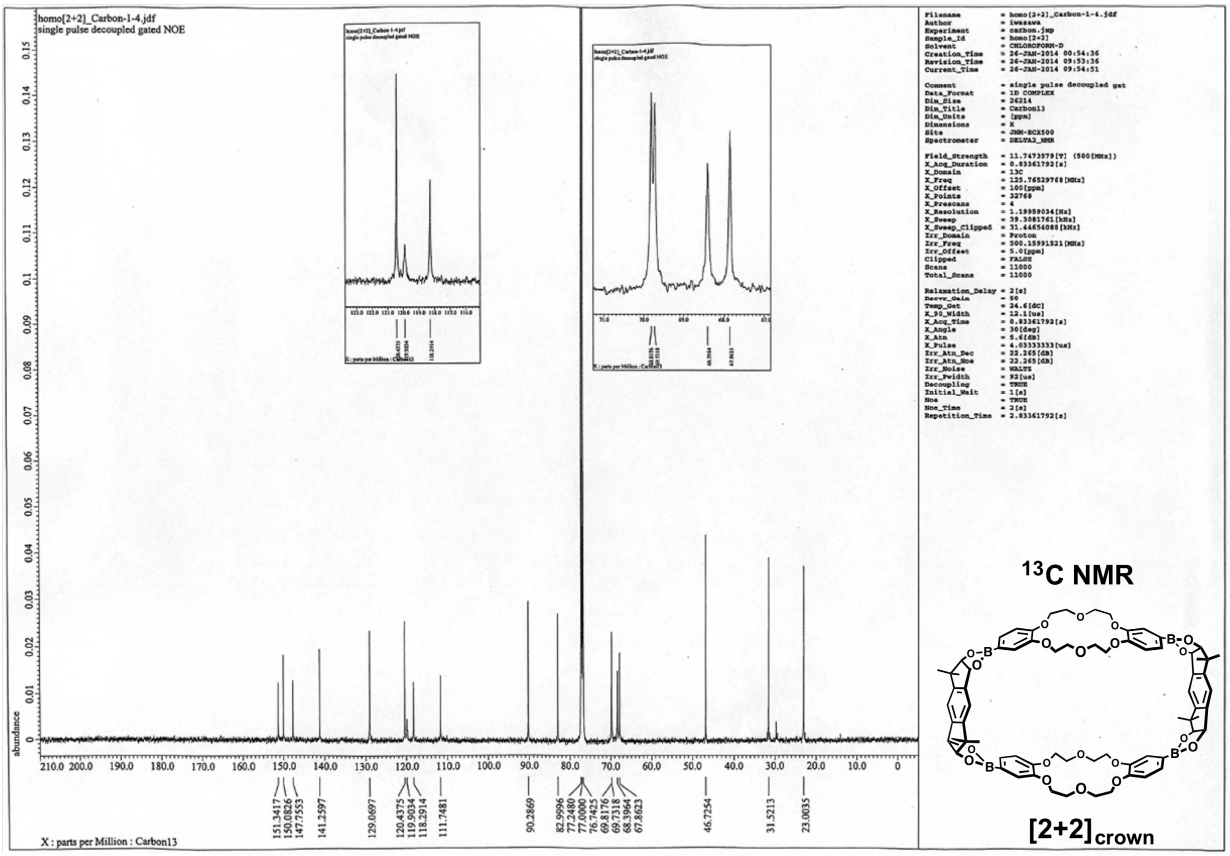
homo[2+2]_Carbon-1-4.jdf
single pulse decoupled gated NOE
151.3417
150.0826
147.7553
141.2597
129.0697
120.4375
119.9034
118.2914
111.7481
90.2869
82.9996
77.2480
77.0000
76.7425
69.8176
69.7318
68.3964
67.8623
46.7254
31.5213
23.0035
X : parts per Million : Carbon13
abundance
13C NMR
[2+2]crown

3. Complexation of [2+2]$_{\mathbf{crown}}$ with 1,4,9,10-Anthradiquinone 1 in the Presence of 2equiv. KOTf

3-1. NMR Study of the Complexation of [2+2]$_{\mathbf{crown}}$ with 1 in the Presence of 2 equiv. of KOTf

The binding behavior of **[2+2]$_{\mathbf{crown}}$** containing alkaline metal salts with 1,4,9,10-anthradiquinone **1** was examined (Fig. S1a). When **[2+2]$_{\mathbf{crown}}$** (6.5 mM), 2 equivalents of KOTf (13.0 mM), and 1 equivalent of **1** (6.5 mM) were mixed in $CDCl_3/CD_3CN$ (0.8 mL, 1:1), a large up-field shift of protons of the guest molecule **1** was observed as shown in Fig. S1b,c. Almost the same degree of upfield shifts of *A*, *B* and *C* of **1** as shown in Fig. S1b,c was due to the shielding effect of the benzene ring of the host framework, suggesting that **1** was included just inside the host molecule with its π-face facing outside as shown in Fig. S1a. This speculation suggests that **1** included in this **[2+2]$_{\mathbf{crown}}$•2K$^+$** could react with appropriate substrates on its π-face, where the reaction might be accelerated by coordination of four carbonyl oxygens of **1** to two K^+ ions. By the addition of another 1 equivalent of **1** to the solution, the peaks derived from **1** in the host shifted downfield and came closer to the peaks of free guest **1** (Fig. S1d). This ^{1}H NMR study suggested 1:1 complexation of **[2+2]$_{\mathbf{crown}}$** with **1** in the presence of 2 equiv. K^+.

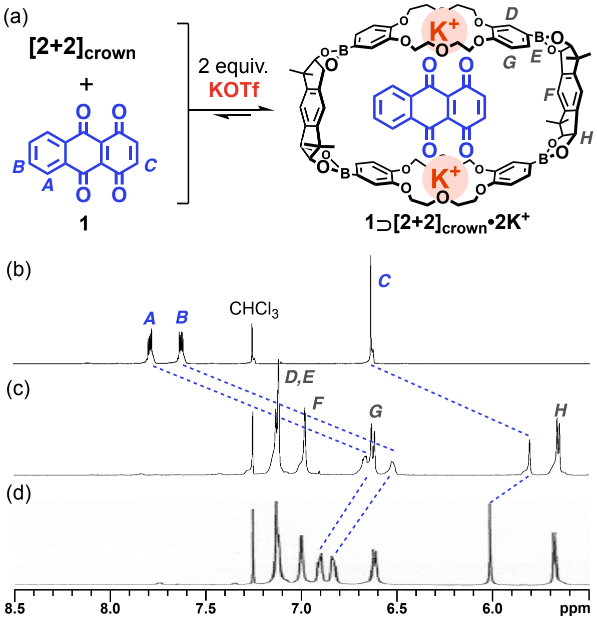

Fig. S1 (a) Complexation of [2+2]$_{\mathbf{crown}}$ with diquinone 1 in the presence of K$^+$. Partial ^{1}H NMR spectra (500 MHz, $CDCl_3/CD_3CN$ (1:1), 298 K) of (b) 1, (c) a mixture of [2+2]$_{\mathbf{crown}}$ (6.5 mM), 1 (6.5 mM), and KOTf (13mM), and (d) a mixture of [2+2]$_{\mathbf{crown}}$ (6.5 mM), 1 (13 mM), and KOTf (13mM).

3-2. ITC Study of the Complexation of with [2+2]$_{\mathbf{crown}}$ with 1 in the Presence of 2 Equiv. of KOTf

A 1 mM $CHCl_3/CH_3CN$ (1:1) solution of **[2+2]$_{\mathbf{crown}}$•2K$^+$** was prepared. Titration of this solution with 60 mM solution of **1** was operated. The titration curve was fitted with a simple 1:1 model and the thermodynamic parameters K_a = 3.54×10^3 M^{-1}, ΔH = –1284 cal/mol, and ΔS = 11.9 cal/mol/deg were

obtained for the association of **[2+2]$_{crown}$•2K$^+$** with **1** (Fig. S2b). The value of binding constant indicated that ca. 80% of the guest **1** was included in 6.5 mM each of **[2+2]$_{crown}$•2K$^+$** and **1** in $CHCl_3/CH_3CN$ (1:1).

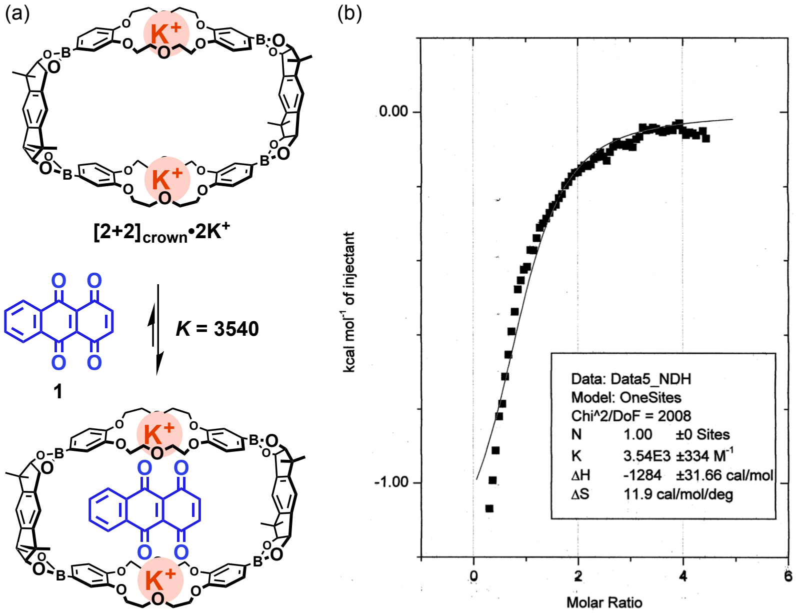

Fig. S2. (a) Complexation of [2+2]$_{crown}$•2K$^+$ with diquinone 1. (b) Titration curve of the addition of 60 mM solution of 1 charged with 1 mM [2+2]$_{crown}$•2K$^+$ in $CHCl_3/CH_3CN$ (1:1) at 25°C. The line represents the curve fitting analysis with the one set of sites model. Thermodynamic parameters are also shown.

3-3. Control Experiments of the Complexation of **[2+2]$_{crown}$** with **1**

The binding behavior of **[2+2]$_{crown}$** containing other alkaline metal salts with 1,4,9,10-anthradiquinone **1** was examined (Fig. 3a). When **[2+2]$_{crown}$** (6.5 mM), 2 equivalents of MOTf (M = Li, Na; 13.0 mM), and 1 equivalent of **1** (6.5 mM) were mixed in $CDCl_3/CD_3CN$ (0.8 mL, 1:1), no shift of the signals of the guest molecule **1** was observed as shown in Fig. S3b-d. A mixture of monomeric pinacol ester of dibenzo-18-crown-6 diboronic acid **2$_{pin}$**, KOTf, and **1** was also examanied. When **2$_{pin}$** (13.0 mM), 1 equivalent of KOTf (13.0 mM) and 0.5 equivalent of **1** (6.5 mM) were mixed in $CDCl_3/CD_3CN$ (0.8 mL, 1:1), no shift of the signals of the guest molecule **1** was observed as shown in Fig. S4b,c. These ^{1}H NMR studies suggested that the macrocyclic structure of **[2+2]$_{crown}$** and K$^+$ ion was essential for the efficient binding of **1**.

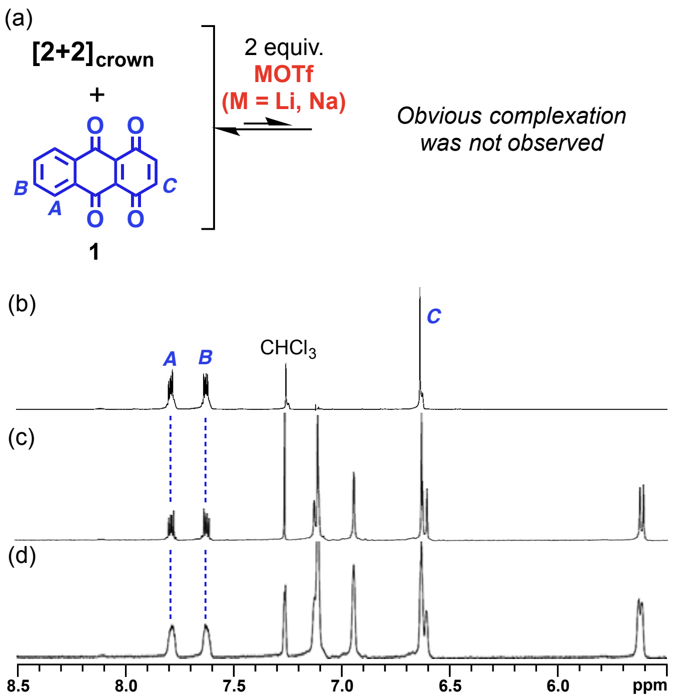

Fig. S3 (a) Examination of complexation of $[2+2]_{crown}$ with diquinone 1 in the presence of MOTf (M = Li, Na). Partial ^{1}H NMR spectra (400 MHz, $CDCl_3/CD_3CN$ (1:1), 298 K) of (b) 1, (c) a mixture of $[2+2]_{crown}$ (6.5 mM), 1 (6.5 mM), and LiOTf (13mM), and (d) a mixture of $[2+2]_{crown}$ (6.5 mM), 1 (6.5 mM), and NaOTf (13mM).

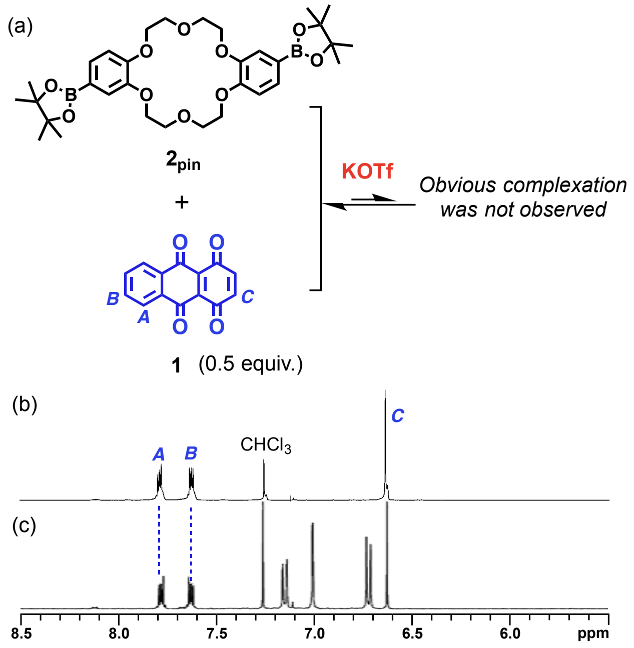

Fig. S4 (a) Examination of complexation of 2_{pin} with diquinone 1 in the presence of KOTf. Partial ^{1}H NMR spectra (400 MHz, $CDCl_3/CD_3CN$ (1:1), 298 K) of (b) 1, and (c) a mixture of 2_{pin} (13 mM), 1 (6.5 mM), and KOTf (13mM).

4. The Diels-Alder Reaction of 1 with Various Dienes

Typical experiment: **1** (5.2 μmol, 6.5 mM), diene (7.8 μmol, 9.8 mM), **[2+2]$_{crown}$** (5.2 μmol, 6.5 mM), and KOTf (10.4 μmol, 13 mM) were dissolved in 0.8 mL of $CDCl_3/CD_3CN$ (1:1) in an NMR tube. The sample was kept at 27 ºC and NMR spectrum was periodically recorded. Concentrations of **1**, products (internal and terminal adduct) and diene were monitored by using 1,4-dinitrobenzene as standard. The second-order rate constants were evaluated using a least-squares computer program (Excel program) from the plot ((1/([diene]$_0$–[**1**]$_0$)ln([diene][**1**]$_0$/[**1**][diene]$_0$) / M^{-1} vs. t /min). Control experiments (no cat.) were carried out under the same conditions but in the absence of **[2+2]$_{crown}$** and KOTf. The purification of the products was performed as follows. To the reaction mixture was added water and the organic material was extracted with dichloromethane. Organic layer was dried over $MgSO_4$, filtered, and concentrated under reduced pressure. The residue was purified by GPC to afford the products.

4-1. The Diels-Alder Reaction of 1 with Anthracene 4 Under Various Conditions

A = 1/([diene]$_0$–[**1**]$_0$)ln([diene][**1**]$_0$/[**1**][diene]$_0$)

Condition: Table 1 entry 1

(a)

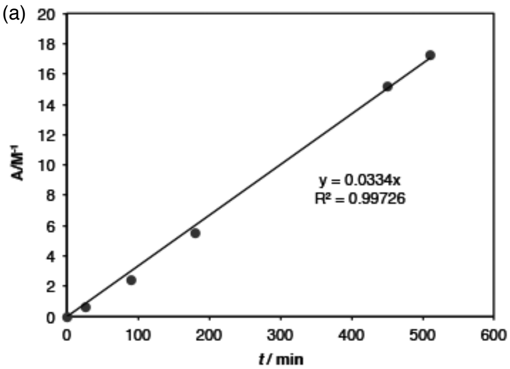

(b)

t/min	Conversion %	[1]	[4]	A/M⁻¹
0	0	0.00656	0.00853	0.0000
26	0.5	0.00653	0.00850	0.5890
90	2	0.00643	0.00840	2.3878
180	4.5	0.00627	0.00823	5.4962
450	11.6	0.00580	0.00777	15.1607
510	13	0.00571	0.00768	17.2286

(c) $k = 0.033\ min^{-1}M^{-1}$

Fig. S5 (a) Second-order plot (A / M^{-1} vs. t / min) (b) table of time dependence of conversion, [1], [4] and A and (c) reaction rate

Condition: Table 1 entry 2

(a)

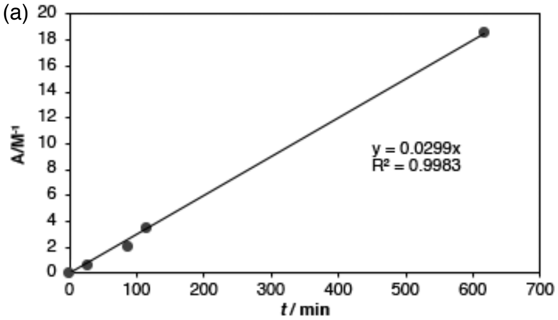

(b)

t/min	Conversion %	[1]	[4]	A/M⁻¹
0	0	0.00656	0.00894	0.0000
27	0.5	0.00653	0.00891	0.5619
88	1.8	0.00644	0.00882	2.0459
115	3	0.00637	0.00874	3.4464
617	14.5	0.00561	0.00799	18.5600

(c) $k = 0.030\ min^{-1}M^{-1}$

Fig. S6 (a) Second-order plot (A / M^{-1} vs. t / min) (b) table of time dependence of conversion, [1], [4] and A and (c) reaction rate

Condition: Table 1 entry 3

(a)

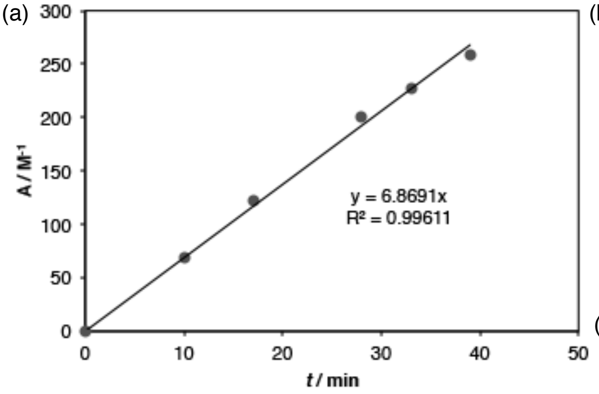

(b)

t/min	Conversion %	[1]	[4]	A/M^{-1}
0	0	0.00656	0.01125	0.0000
10	48	0.00341	0.00810	69.4334
17	65	0.00230	0.00698	122.2846
28	79	0.00138	0.00607	201.1827
33	82	0.00118	0.00587	227.0322
39	85	0.00098	0.00567	258.6519
90	95	0.00033	0.00502	466.8146

(c) $k = 6.87\ \mathrm{min^{-1}M^{-1}}$

Fig. S7 (a) Second-order plot (A / M^{-1} vs. t / min) (b) table of time dependence of conversion, [1], [4] and A and (c) reaction rate

Condition: Table 1 entry 4

(a)

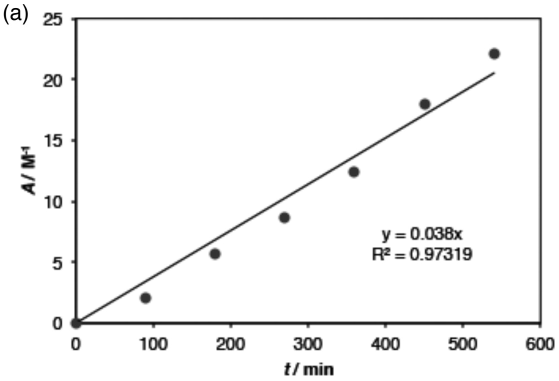

(b)

t/min	Conversion %	[1]	[4]	A/M^{-1}
0	0	0.00656	0.00886	0.0000
90	1.8	0.00644	0.00874	2.0646
180	4.8	0.00625	0.00855	5.6556
270	7.2	0.00609	0.00839	8.6726
360	10	0.00591	0.00820	12.3672
450	14	0.00564	0.00794	18.0019
540	16.7	0.00547	0.00776	22.0657

(c) $k = 0.038\ \mathrm{min^{-1}M^{-1}}$

Fig. S8 (a) Second-order plot (A / M^{-1} vs. t / min) (b) table of time dependence of conversion, [1], [4] and A and (c) reaction rate

Condition: Table 1 entry 5

(a)

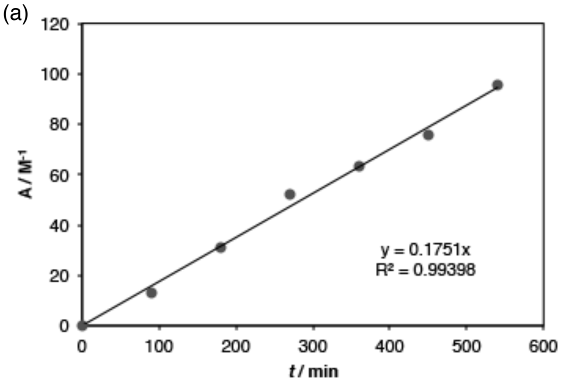

(b)

t/min	Conversion %	[1]	[4]	A/M^{-1}
0	0	0.00656	0.01330	0.0000
90	4.8	0.00625	0.01299	12.8225
180	11	0.00584	0.01258	30.8748
270	17.5	0.00541	0.01215	51.8904
360	20.7	0.00520	0.01194	63.1400
450	24	0.00499	0.01173	75.4450
540	29	0.00466	0.01140	95.6129

(c) $k = 0.175\ \mathrm{min^{-1}M^{-1}}$

Fig. S9 (a) Second-order plot (A / M^{-1} vs. t / min) (b) table of time dependence of conversion, [1], [4] and A and (c) reaction rate

In the reaction of anthracene (Table 1, entry 3), the signal of the anthracene slightly up-field shifted compared to free anthracene in ^{1}H NMR spectra, suggesting the formation of a weak Michaelis complex **1•4@[2+2]$_{crown}$•2K^{+}** (Fig. S10a,b).

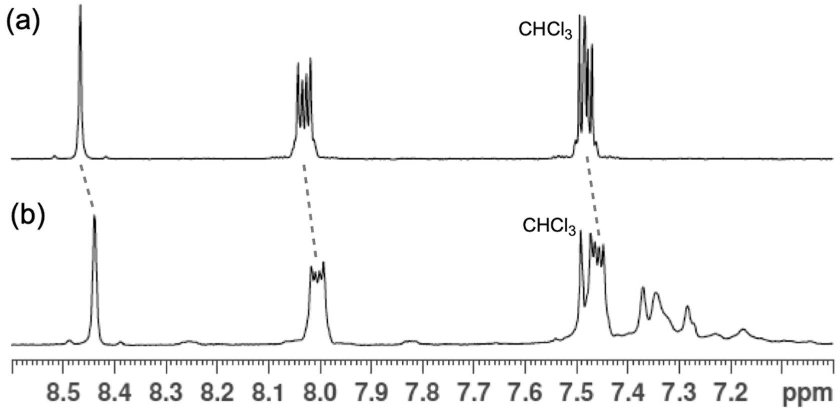

Fig. S10 Partial ^{1}H NMR spectra (400 MHz, $CDCl_3$/CD_3CN (1:1)) of (a) 4 and (b) a mixture of [2+2]$_{crown}$ (6.5 mM), 1 (6.5 mM), 4 (9.8 mM), and KOTf (13.0 mM). (Residual TMS was set at 0 ppm.)

When **1** (0.65 mM) and **4** (0.65 mM) were mixed in $CDCl_3$/CD_3CN (1:1), no shift of the signals was observed (Fig. S11).

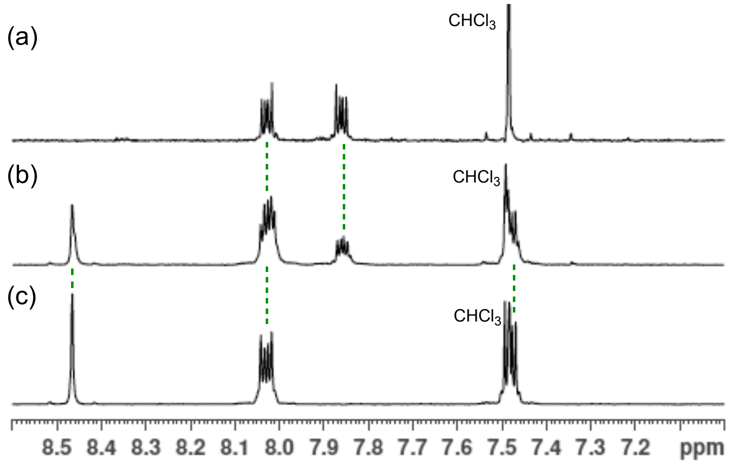

Fig. S11 Partial ^{1}H NMR spectra (400 MHz, $CDCl_3$/CD_3CN (1:1)) of (a) 1, (b) a mixture of 1 (6.5 mM), and 4 (6.5 mM) and (c) 4 . (Residual TMS was set at 0 ppm.)

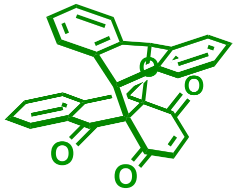

Physical data of **5$_{int}$**

^{1}H NMR (500 MHz, $CDCl_3$): δ 7.75-7.74 (m, 2H), 7.62-7.60 (m, 2H), 7.34-7.31 (m, 4H), 7.14-7.09 (m, 2H), 7.01-6.99 (m, 2H), 6.36 (s, 2H), 5.72 (s, 2H).

^{13}C NMR (125 MHz, $CDCl_3$): δ 190.0, 188.5, 139.5, 139.4, 139.2, 134.8, 134.7, 127.2, 127.0, 125.6, 125.5, 71.5, 54.2.

HRMS (FAB$^+$, NBA): *m/z* Calcd. for $C_{28}H_{17}O_4$: 417.1127., Found: 417.1134 $[M+H]^+$.

IR (ATR): 1705, 1607, 1593, 1274, 1244, 1087, 1045, 1003 cm^{-1}.

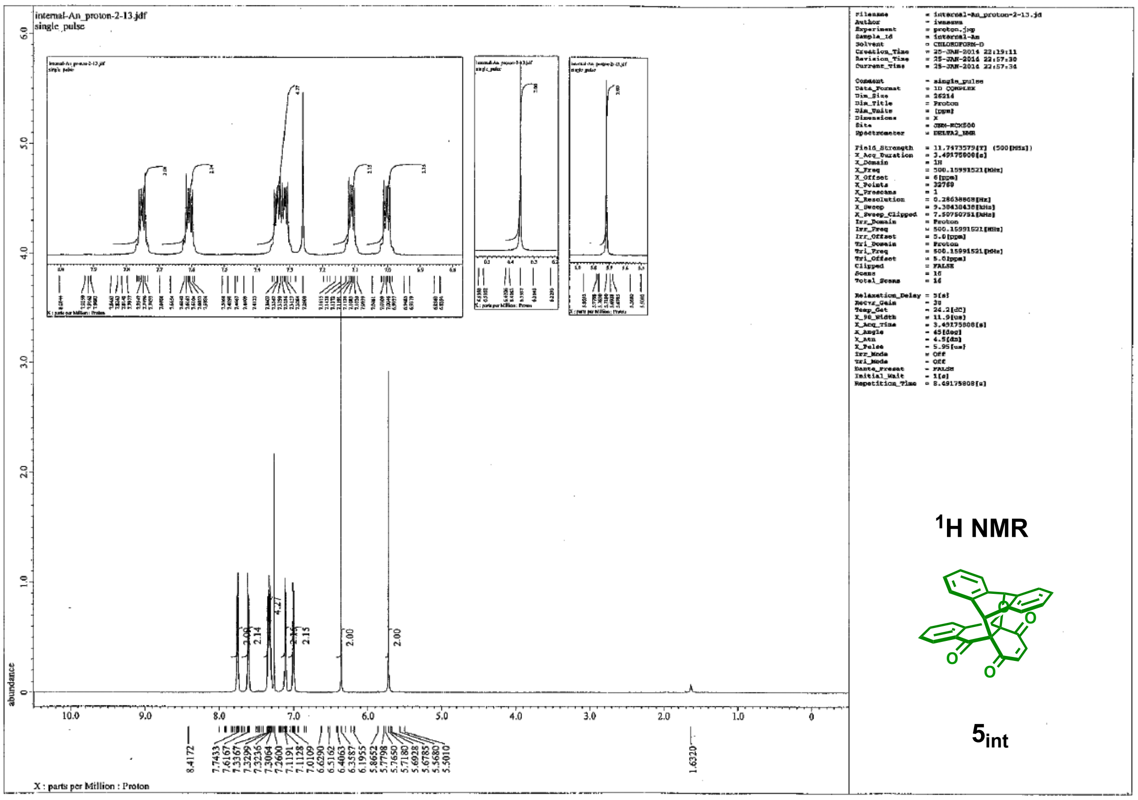

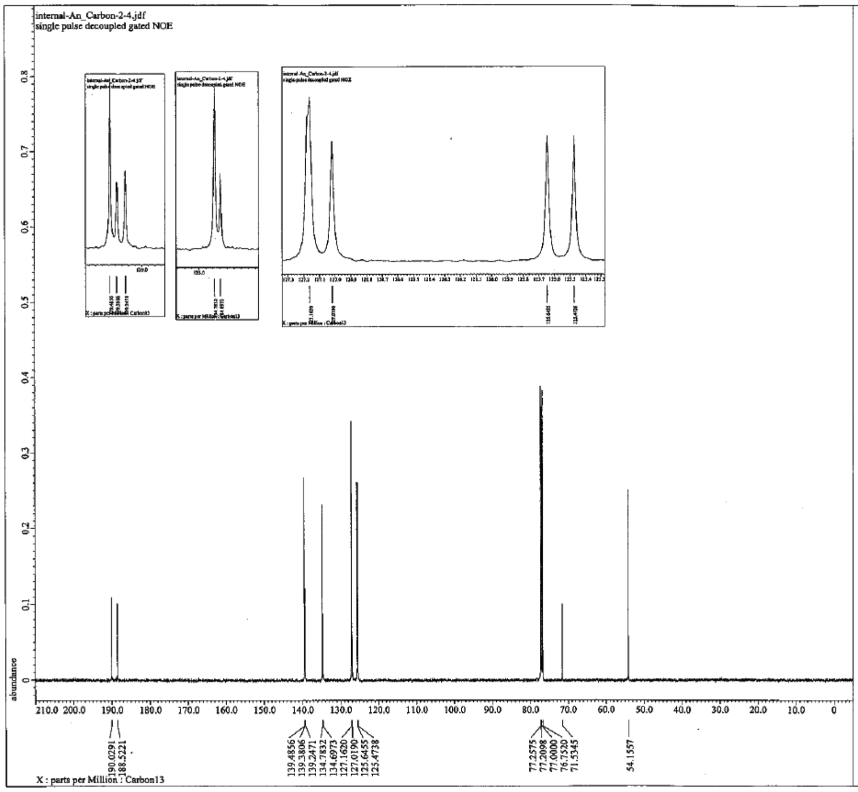

^{13}C NMR

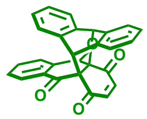

$\mathbf{5_{int}}$

The binding behavior of **[2+2]$_{\mathbf{crown}}$** containing K^+ with **5$_{\mathbf{int}}$** was examined (Fig. S12a). **[2+2]$_{\mathbf{crown}}$** (6.5 mM), 2 equivalents of KOTf (13.0 mM), and 1 equivalent of **5$_{\mathbf{int}}$** (6.5 mM) were mixed in $CDCl_3/CD_3CN$ (0.8 mL, 1:1) and ^{1}H NMR spectra were measured at 303 K and 323 K (Fig. S12c,d). Upfield shift and broadening of proton signals of **5$_{\mathbf{int}}$** was observed (Fig. 12b,c). These signals also became diastereotopic and the separation of signals was observed due to the chirality of the **[2+2]$_{\mathbf{crown}}$**, suggesting **5$_{\mathbf{int}}$** was included in the host. From the ITC study, association constant of **5$_{\mathbf{int}}$** with **[2+2]$_{\mathbf{crown}}$•2K$^+$** was measured. A 1 mM $CHCl_3/CH_3CN$ (1:1) solution of **[2+2]$_{\mathbf{crown}}$•2K$^+$** was titrated with 30 mM solution of **5$_{\mathbf{int}}$**. The titration curve was fitted with a simple 1:1 model and the thermodynamic parameters $K_a = 1.08\times10^3$ M^{-1}, $\Delta H = -3463$ cal/mol, and $\Delta S = 2.76$ cal/mol/deg were obtained for the association of **[2+2]$_{\mathbf{crown}}$•2K$^+$** with **5$_{\mathbf{int}}$** (Fig. S12e).

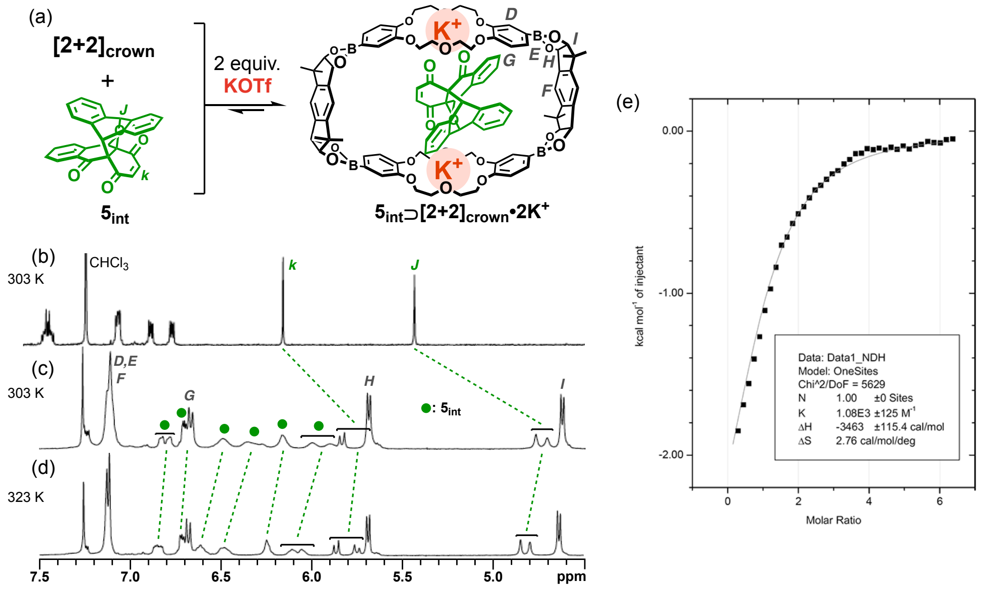

Fig. S12 (a) Complexation of [2+2]$_{\mathbf{crown}}$ with 5$_{\mathbf{int}}$ in the presence of K$^+$. Partial ^{1}H NMR spectra (400 MHz, $CDCl_3/CD_3CN$ (1:1)) of (b) 5$_{\mathbf{int}}$ at 303 K, (c) a mixture of [2+2]$_{\mathbf{crown}}$ (6.5 mM), 5$_{\mathbf{int}}$ (6.5 mM), and KOTf (13mM) at 303 K, and (d) the same sample at 323 K. (e) Titration curve of [2+2]$_{\mathbf{crown}}$•2K$^+$ with 5$_{\mathbf{int}}$ in $CHCl_3/CH_3CN$ (1:1) at 25°C. The line represents the curve fitting analysis with the one set of sites model. Thermodynamic parameters are also shown.

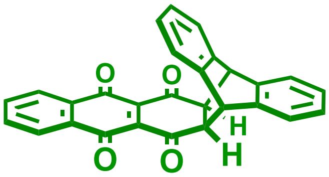

Physical data of **5**$_{ter}$

^{1}H NMR (500 MHz, $CDCl_3$): δ 7.97-7.94 (m, 2H), 7.77-7.74 (m, 2H), 7.42-7.40 (m, 2H), 7.24-7.22 (m, 4H), 7.21-7.19 (m, 2H), 6.83-6.79 (m, 2H), 4.80 (s, 2H), 3.34 (s, 2H).

^{13}C NMR (125 MHz, $CDCl_3$): δ 194.9, 181.7, 140.6, 139.2, 135.0, 134.6, 131.1, 127.4, 127.1, 126.3, 125.9, 124.2, 53.1, 47.7.

HRMS (FAB$^+$, NBA): *m/z* Calcd. for $C_{28}H_{17}O_4$: 417.1127., Found: 417.1109 $[M+H]^+$.

IR (ATR): 1705, 1607, 1593, 1274, 1244, 1087, 1045, 1003 cm^{-1}.

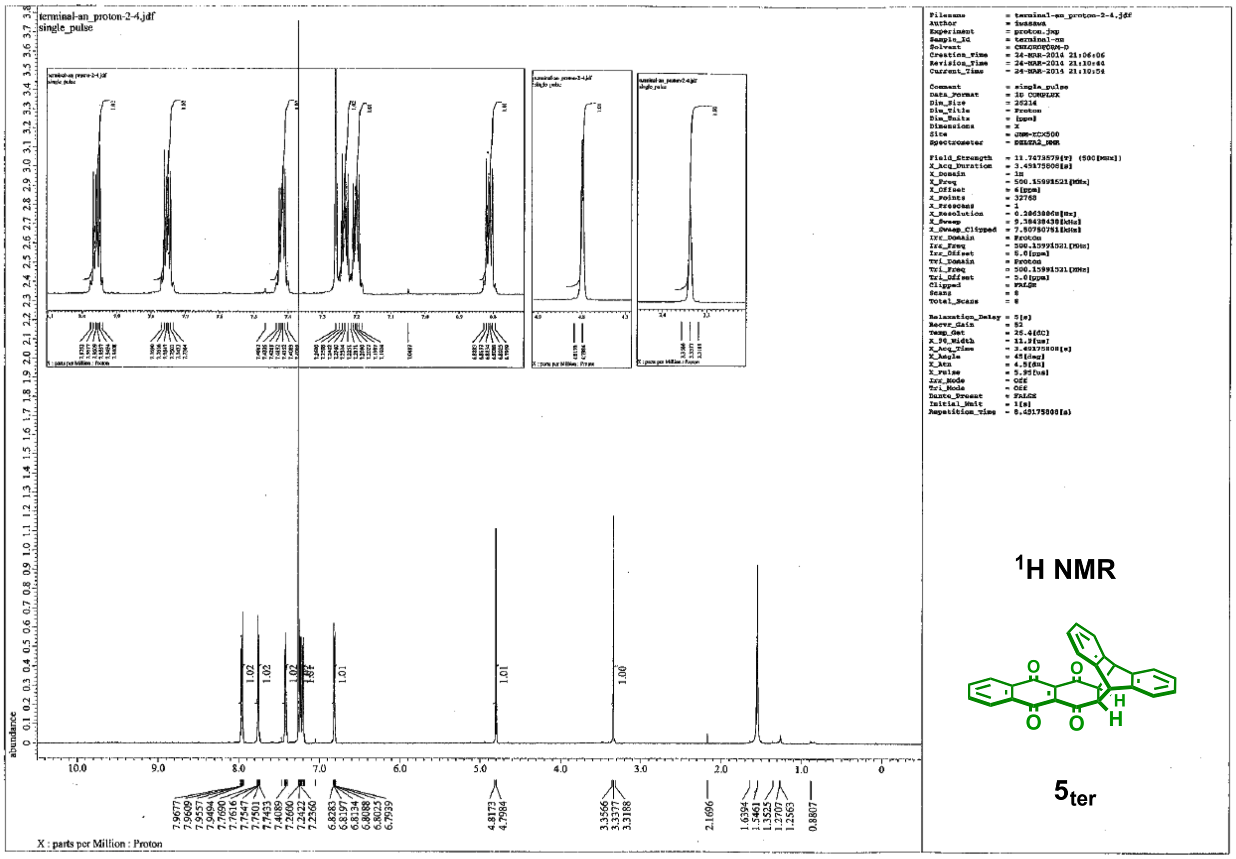

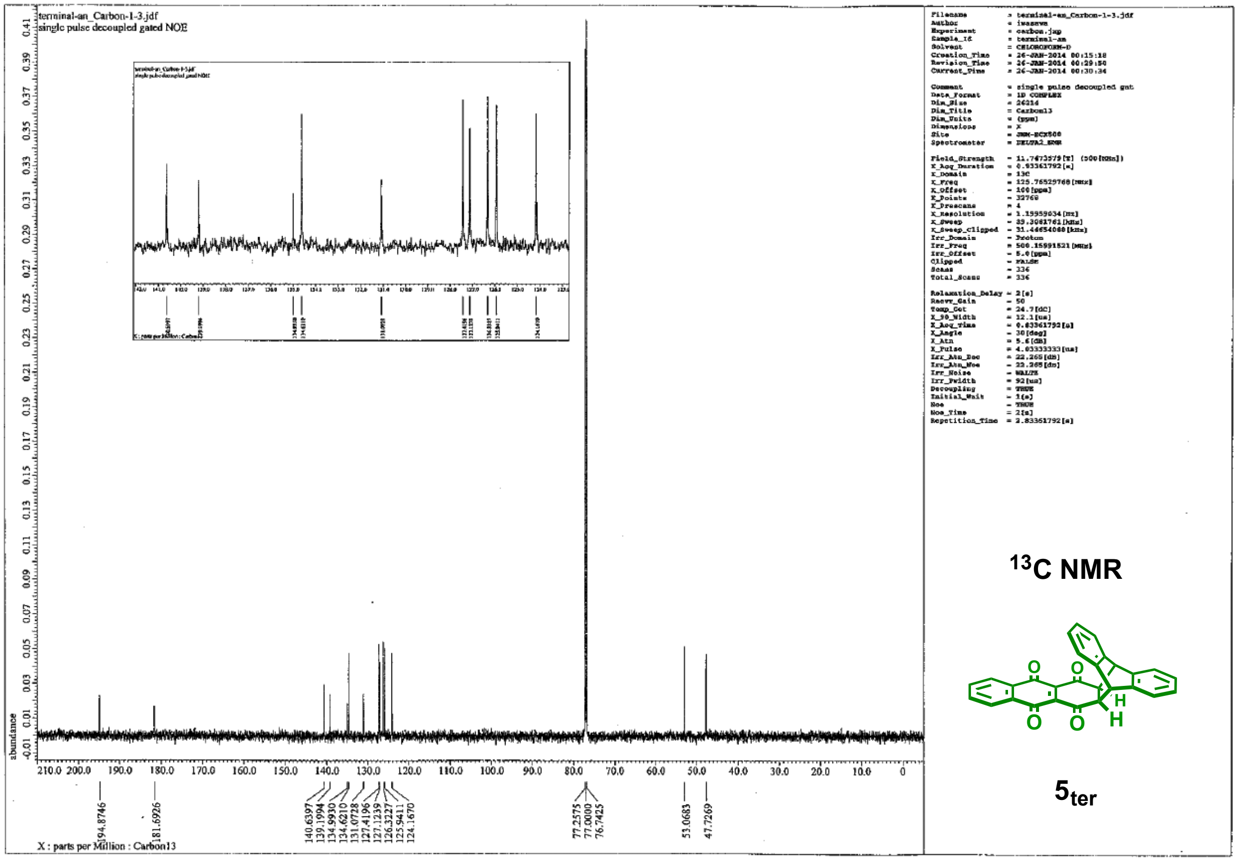

4-2-1. The Diels-Alder Reaction of **1** with 2-Mono and 2,3-Di-substituted 1,3-Butadienes

$A = 1/([\text{diene}]_0 - [\mathbf{1}]_0)\ln([\text{diene}][\mathbf{1}]_0/[\mathbf{1}][\text{diene}]_0)$

Condition: Table 2 entry 1

(a)

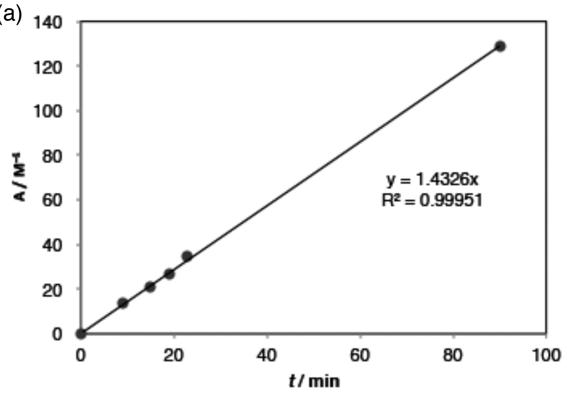

(b)

t/min	Conversion %	[1]	[6]	A/M⁻¹
0	0	0.00560	0.01743	0.0000
9	20.5	0.00522	0.01608	13.7196
15	29	0.00466	0.01553	20.8773
19	35	0.00427	0.01513	26.6385
23	42.5	0.00377	0.01464	34.8780
90	83	0.00112	0.01198	128.5799

(c) $k = 1.43\ \text{min}^{-1}\text{M}^{-1}$

Fig. S13 (a) Second-order plot (A / M^{-1} vs. t / min) (b) table of time dependence of conversion, [1], [6] and A and (c) reaction rate

Condition: Table 2 entry 2

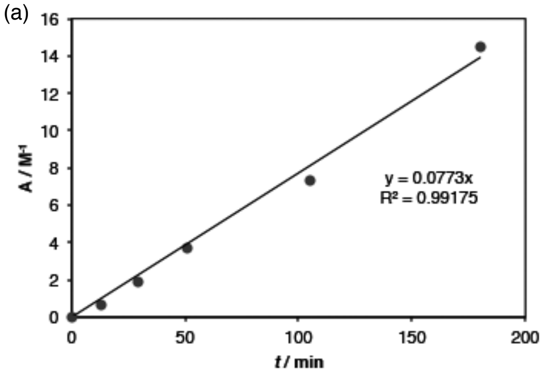

(b)

t/min	Conversion %	[1]	[6]	A/M^{-1}
0	0	0.01500	0.04500	0.0000
13	3	0.01455	0.04455	0.6803
29	8	0.01380	0.04380	1.8784
51	15	0.01275	0.04275	3.7075
105	27	0.01095	0.04095	7.3467
180	45	0.00825	0.03825	14.5106

(c) $k = 0.077$ min^{-1} M^{-1}

Fig. S14 (a) Second-order plot (A / M^{-1} vs. t / min) (b) table of time dependence of conversion, [1], [6] and A and (c) reaction rate

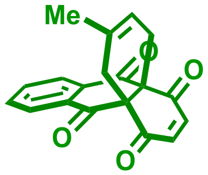

Physical data of **S1$_{int}$**

^{1}H NMR (500 MHz, CDCl$_3$): δ 8.10-8.07 (m, 2H), 7.79-7.76 (m, 2H), 6.78 (br, 4H), 5.42 (br, 1H), 2.64 (br, 2H), 2.53 (br, 2H), 1.71 (s, 3H).

^{13}C NMR (125 MHz, CDCl$_3$): δ 193.5, 191.9, 139.5, 135.2, 133.2, 130.8, 127.9, 117.2, 65.6, 64.7, 31.7, 27.8, 23.0.

HRMS (FAB$^+$, NBA): *m/z* Calcd. for $C_{19}H_{15}O_4$: 307.0971., Found: 307.0944 [M+H]$^+$.

IR (ATR): 1710, 1679, 1592, 1259, 1234 cm^{-1}.

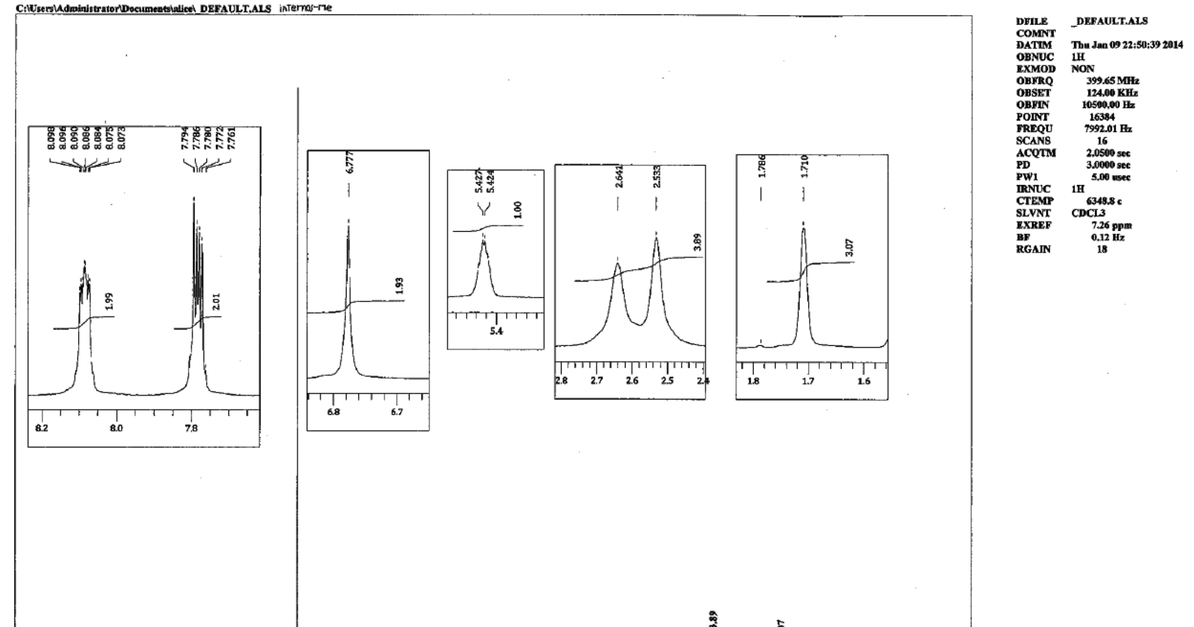

^{1}H NMR

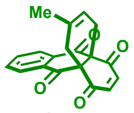

S1$_{int}$

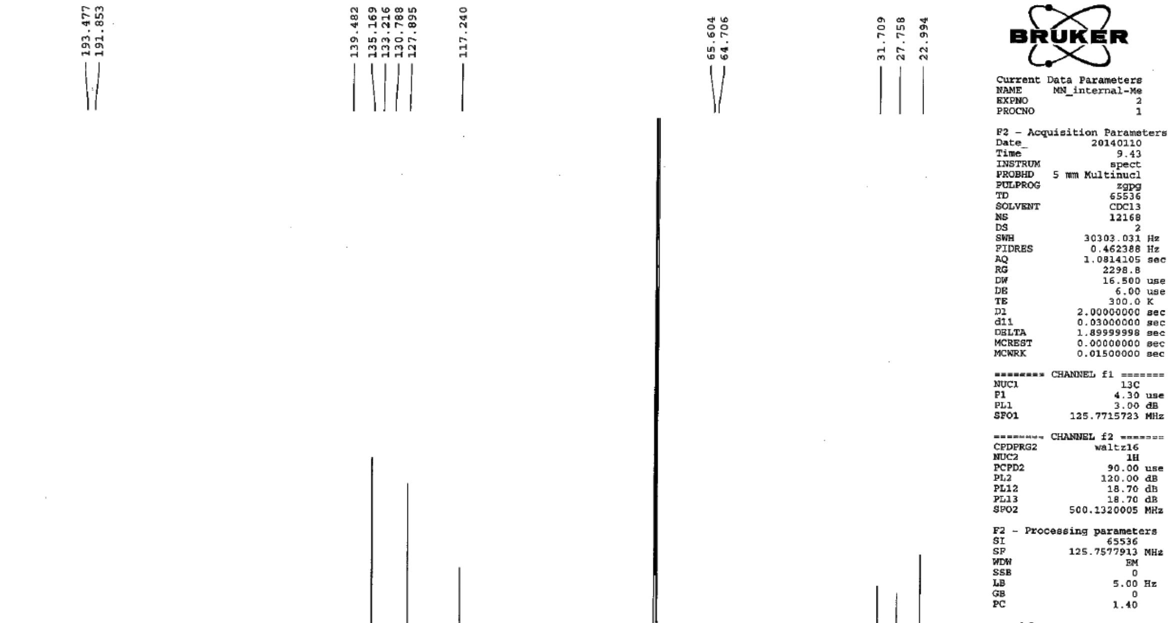

^{13}C NMR

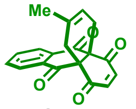

S1$_{int}$

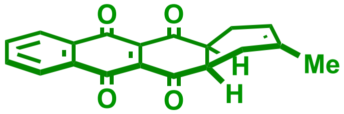

Physical data of **S1$_{ter}$**

^{1}H NMR (500 MHz, $CDCl_3$): δ 8.07-8.05 (m, 2H), 7.83-7.80 (m, 2H), 5.41 (br, 1H), 3.55 (q, J = 5.4 Hz, 1H), 3.45 (q, J = 5.4 Hz, 1H), 2.41 (td, J = 18.1, 5.0 Hz, 2H), 2.26-2.23 (m, 1H), 2.14 (dd, J = 17.5, 5.0 Hz, 1H), 1.70 (s, 3H).

^{13}C NMR (125 MHz, $CDCl_3$): δ 196.8, 196.4, 183.4, 183.3, 134.8, 133.8, 133.5, 131.9, 131.8, 131.3, 126.5, 117.8, 49.8, 49.1, 28.2, 24.1, 23.3.

HRMS (FD$^+$): *m/z* Calcd. for $C_{19}H_{14}O_4$: 306.0892., Found: 306.0868 $[M]^+$.

IR (ATR): 1708, 1665, 1591, 1284, 1179 cm^{-1}.

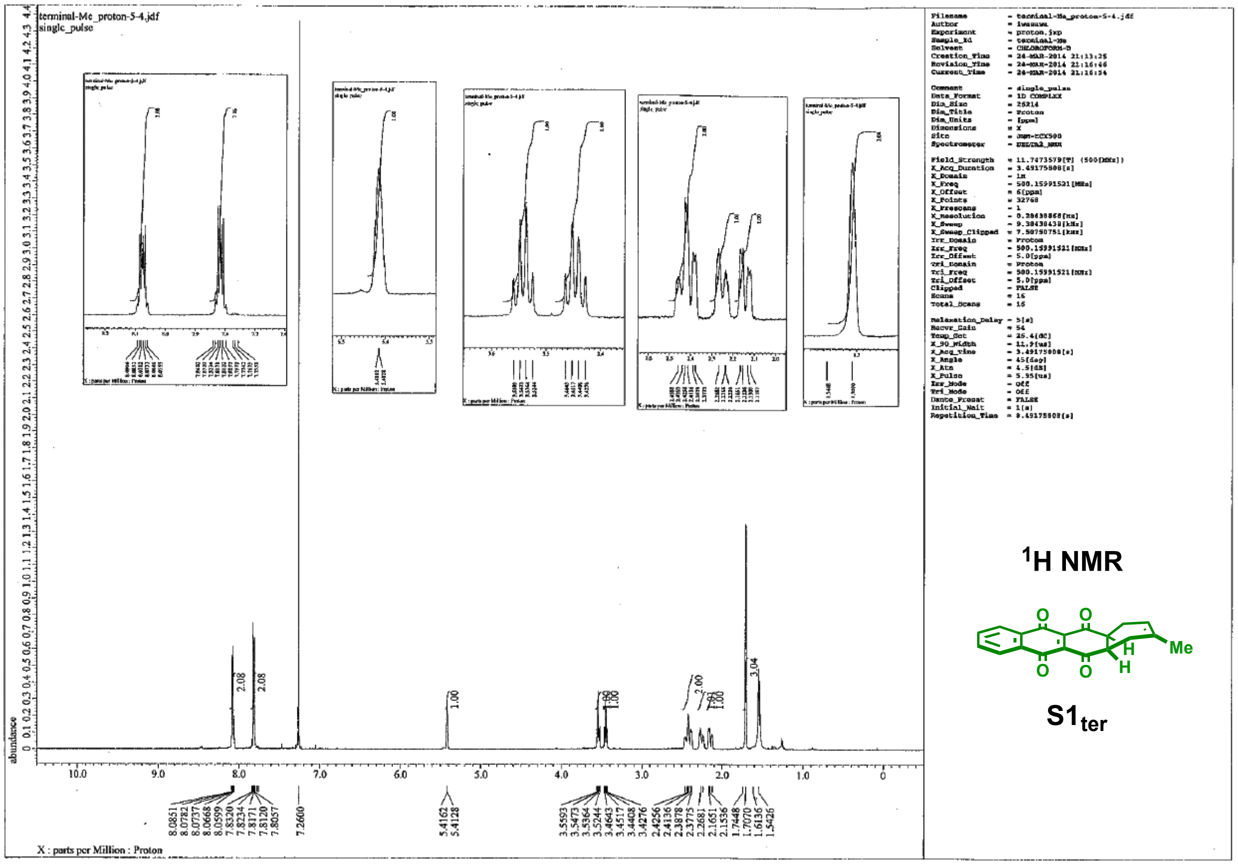

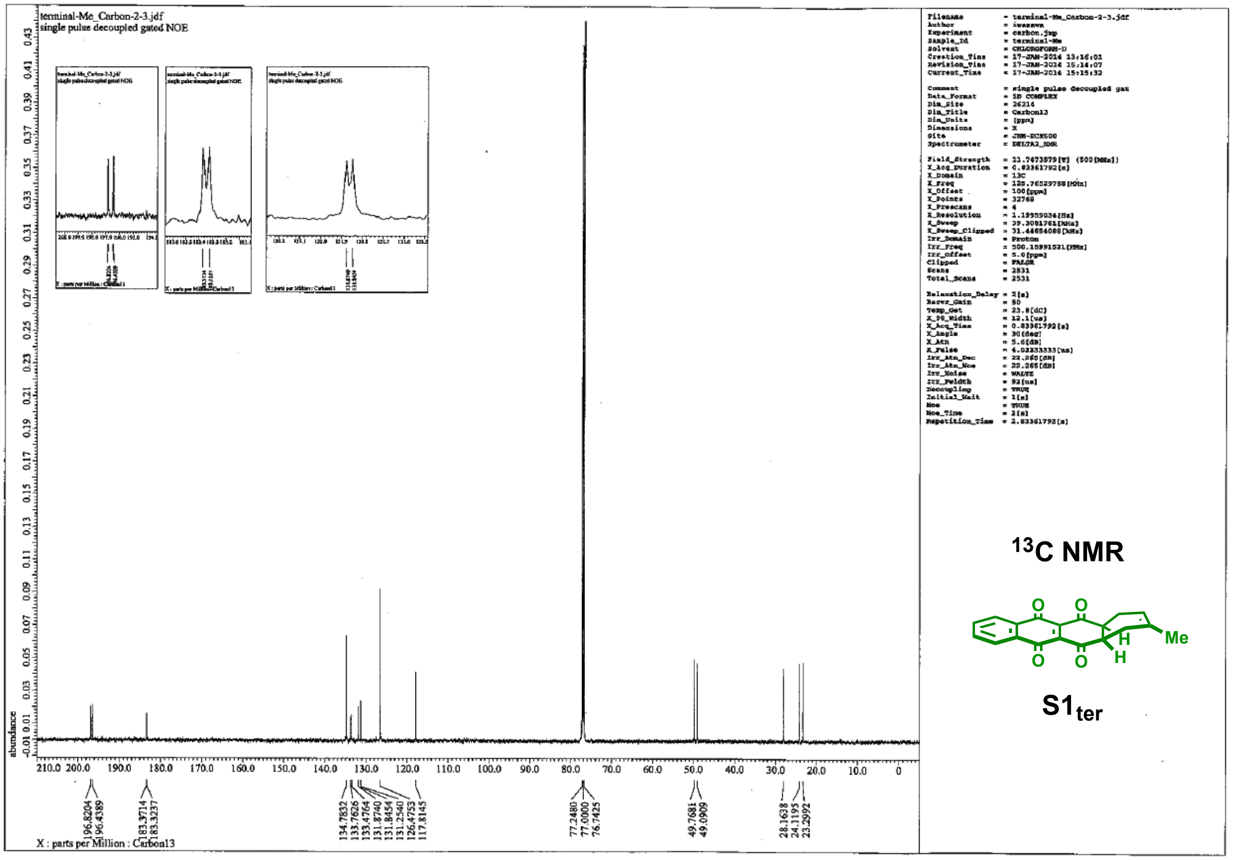

Condition: Table 2 entry 3

(a)

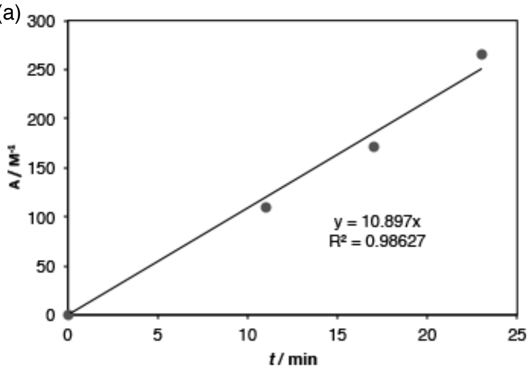

(b)

t/min	Conversion %	[1]	[7]	A/M⁻¹
0	0	0.00656	0.01076	0.0000
11	60	0.00263	0.00682	109.7686
17	73	0.00177	0.00597	171.5903
23	84	0.00105	0.00525	265.5539

(c) $k = 10.90\ \mathrm{min^{-1}\,M^{-1}}$

Fig. S15 (a) Second-order plot (A / M^{-1} vs. t / min) (b) table of time dependence of conversion, [1], [7] and A and (c) reaction rate

Condition: Table 2 entry 4

(a)

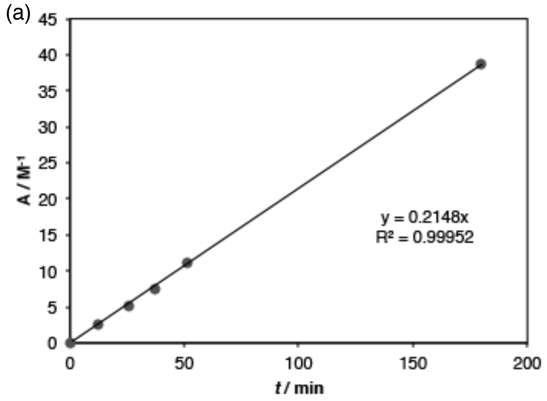

(b)

t/min	Conversion %	[1]	[7]	A/M-1
0	0	0.01500	0.02250	0.0000
12	5.5	0.01418	0.02168	2.5619
26	10.5	0.01343	0.02093	5.1148
37	14.8	0.01278	0.02028	7.5051
51	20.8	0.01188	0.01938	11.1894
180	50.3	0.00746	0.01496	38.7594

(c) $k = 0.21\ \text{min}^{-1}\ \text{M}^{-1}$

Fig. S16 (a) Second-order plot (A / M^{-1} vs. t / min) (b) table of time dependence of conversion, [1], [7] and A and (c) reaction rate

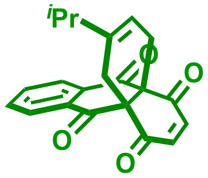

Physical data of **S2$_{int}$**

^{1}H NMR (500 MHz, $CDCl_3$): δ 8.10-8.07 (m, 2H), 7.80-7.77 (m, 2H), 6.80-6.75 (m, 2H), 5.42 (br, 1H), 2.66 (br, 2H), 2.54 (br, 2H), 2.23 (sep, J = 6.9 Hz, 1H), 1.01 (d, J = 6.9 Hz, 6H).

^{13}C NMR (125 MHz, $CDCl_3$): δ 193.6, 192.1, 191.7, 140.2, 139.6, 139.3, 135.1, 133.2, 127.9, 114.5, 65.5, 64.9, 34.7, 28.6, 27.6, 20.8.

HRMS (FAB$^+$, NBA): m/z Calcd. for $C_{21}H_{19}O_4$: 334.1283., Found: 335.1279 $[M+H]^+$.

IR (ATR): 2961, 2927, 1710, 1683, 1592, 1465, 1424, 1363, 1259, 1235, 1083 cm^{-1}.

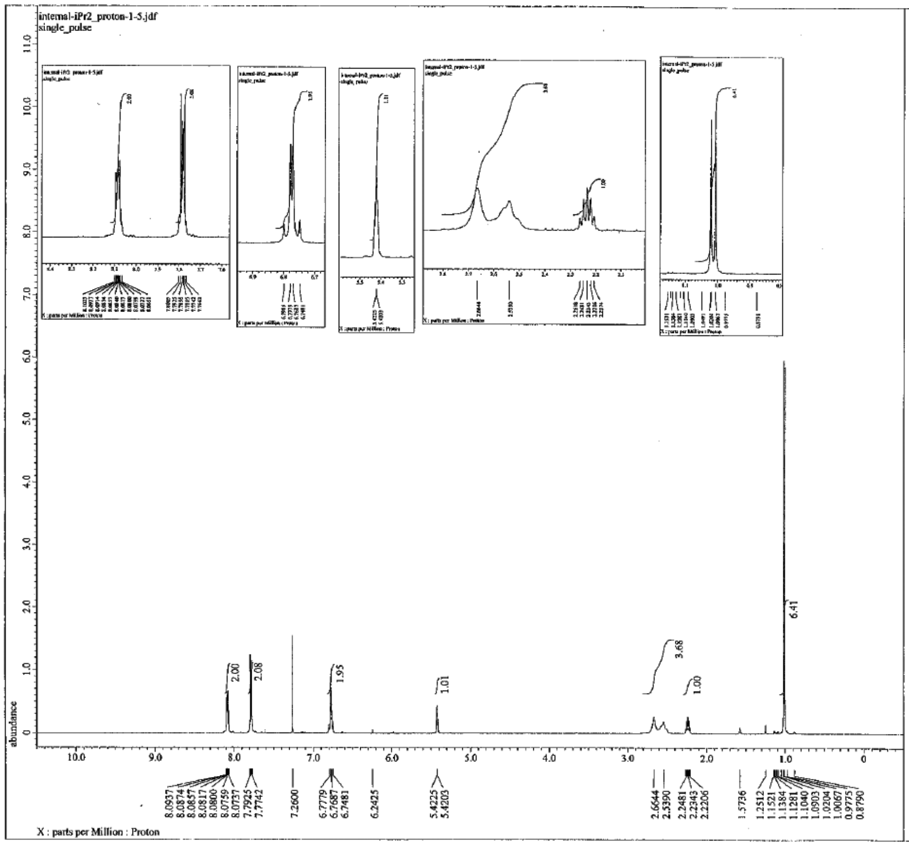

^{1}H NMR

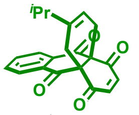

S2$_{int}$

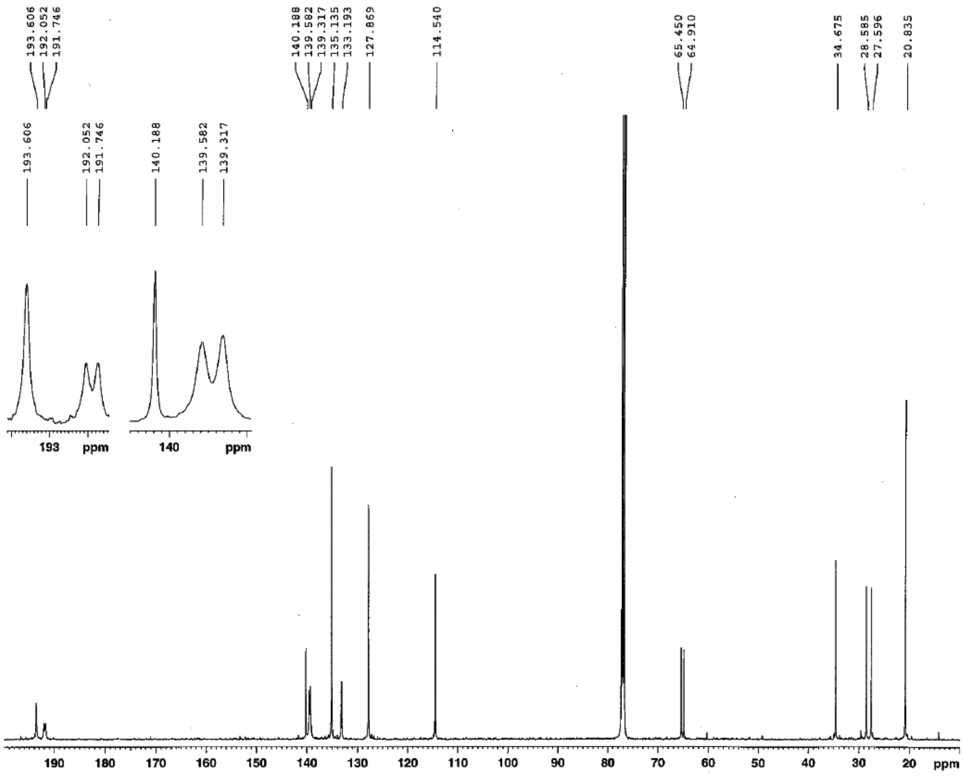


```
Current Data Parameters
NAME        MN_internal-iPr
EXPNO                     2
PROCNO                    1

F2 - Acquisition Parameters
Date_              20131226
Time                   9.44
INSTRUM               spect
PROBHD      5 mm Multinucl
PULPROG                zgpg
TD                    65536
SOLVENT               CDCl3
NS                    13070
DS                        2
SWH               30303.031 Hz
FIDRES             0.462388 Hz
AQ                1.0814105 sec
RG                     3251
DW                   16.500 use
DE                     6.00 use
TE                    300.0 K
D1               2.00000000 sec
d11              0.03000000 sec
DELTA            1.89999998 sec
MCREST           0.00000000 sec
MCWRK            0.01500000 sec

======== CHANNEL f1 ========
NUC1                    13C
P1                     4.30 use
PL1                    3.00 dB
SFO1            125.7715723 MHz

======== CHANNEL f2 ========
CPDPRG2             waltz16
NUC2                     1H
PCPD2                 90.00 use
PL2                  120.00 dB
PL12                  18.70 dB
PL13                  18.70 dB
SFO2            500.1320005 MHz

F2 - Processing parameters
SI                    65536
SF              125.7577932 MHz
WDW                      EM
SSB                       0
LB                     5.00 Hz
GB                        0
PC                     1.40
```

^{13}C NMR

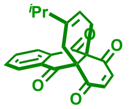

S2$_{int}$

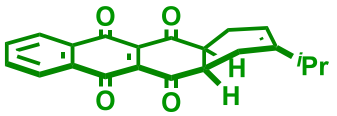

Physical data of **S2**$_{ter}$

^{1}H NMR (500 MHz, $CDCl_3$): δ 8.09-8.06 (m, 2H), 7.82-7.79 (m, 2H), 5.42 (br, 1H), 3.54-3.50 (m, 1H), 3.49-3.45 (m, 1H), 2.48-2.40 (m, 2H), 2.29-2.15 (m, 3H), 1.03-1.00 (m, 6H).

^{13}C NMR (125 MHz, $CDCl_3$): δ 196.8, 196.6, 183.4, 183.4, 140.8, 134.8, 133.7, 133.4, 131.9, 126.5, 115.1, 49.9, 49.5, 34.9, 24.6, 24.0, 21.1, 21.0.

HRMS (FAB$^+$, NBA): *m/z* Calcd. for $C_{21}H_{18}O_4$: 334.1205., Found: 334.1204 $[M]^+$.

IR (ATR): 1707, 1663, 1592, 1281, 1221 cm^{-1}.

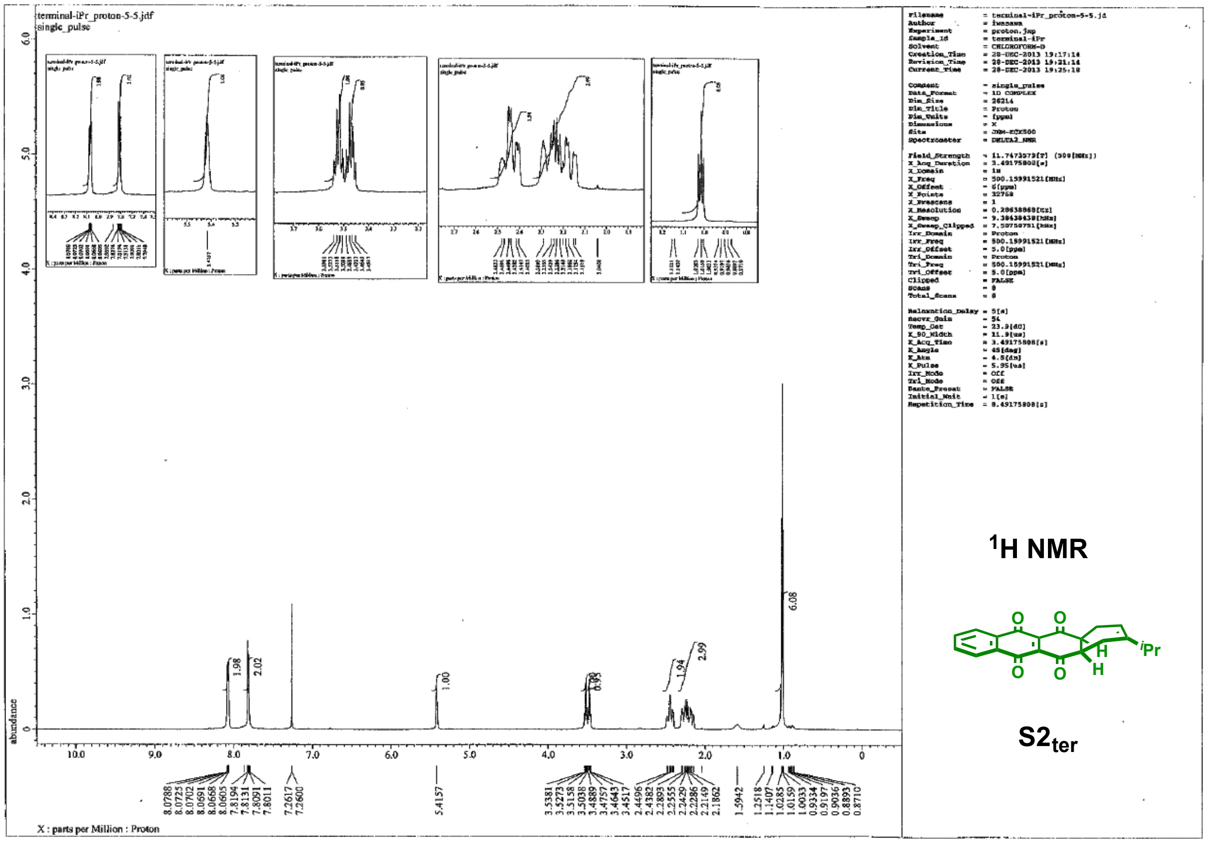

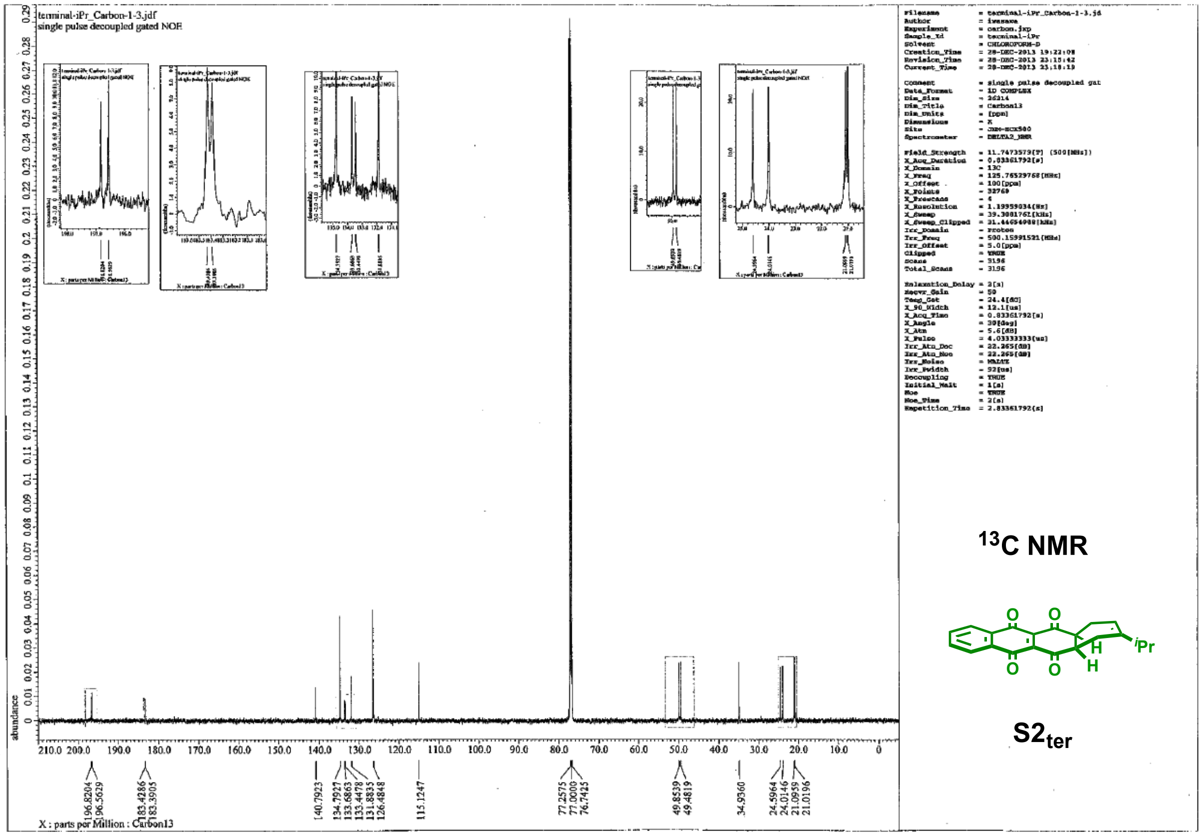

Condition: Table 2 entry 5

(a)

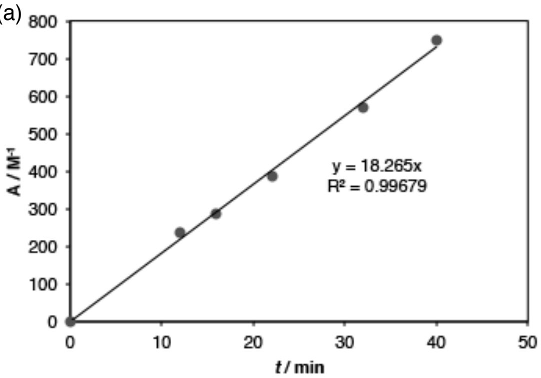

(b)

t/min	Conversion %	[1]	[8]	A/M^{-1}
0	0	0.00656	0.00938	0.0000
12	76	0.00158	0.00439	237.2985
16	80.6	0.00127	0.00409	287.5593
22	86.7	0.00087	0.00369	385.0140
32	93	0.00046	0.00328	570.6877
40	96	0.00026	0.00308	747.3511

(c) $k = 18.27\ \text{min}^{-1}\,\text{M}^{-1}$

Fig. S17 (a) Second-order plot (A / M^{-1} vs. t / min) (b) table of time dependence of conversion, [1], [8] and A and (c) reaction rate

Condition: Table 2 entry 6

(a)

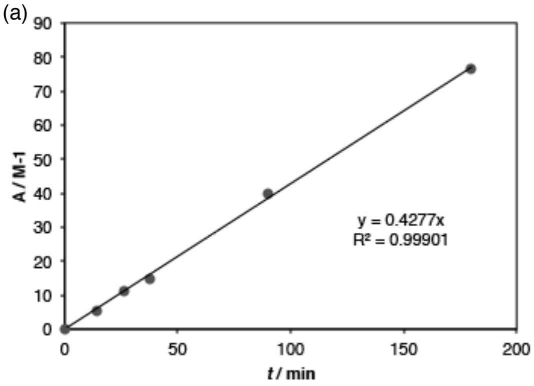

(b)

t/min	Conversion %	[1]	[8]	A/M⁻¹
0	0	0.01500	0.02250	0.0000
14	11	0.01335	0.02085	5.3830
26	21	0.01185	0.01935	11.3199
38	26	0.01110	0.01860	14.7668
90	51	0.00735	0.01485	39.7113
180	70	0.00450	0.01200	76.7152

(c) $k = 0.43\ \mathrm{min}^{-1}\,\mathrm{M}^{-1}$

Fig. S18 (a) Second-order plot (A / M^{-1} vs. t / min) (b) table of time dependence of conversion, [1], [8] and A and (c) reaction rate

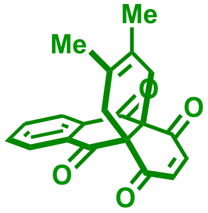

Physical data of **$S3_{int}$**

^{1}H NMR (500 MHz, $CDCl_3$): δ 8.09-8.07 (m, 2H), 7.79-7.77 (m, 2H), 6.77 (s, 2H), 2.55 (br, 4H), 1.64 (s, 6H).

^{13}C NMR (125 MHz, $CDCl_3$): δ 193.6, 191.9, 139.5, 135.1, 133.2, 127.8, 122.5, 65.6, 33.2, 18.6.

HRMS (FAB$^+$, NBA): *m/z* Calcd. for $C_{20}H_{16}O_4$: 320.1049., Found: 320.1079 $[M]^+$.

IR (ATR): 2915, 2873, 1712, 1678, 1592, 1425, 1259, 1240, 1081, 1066, 859 cm^{-1}.

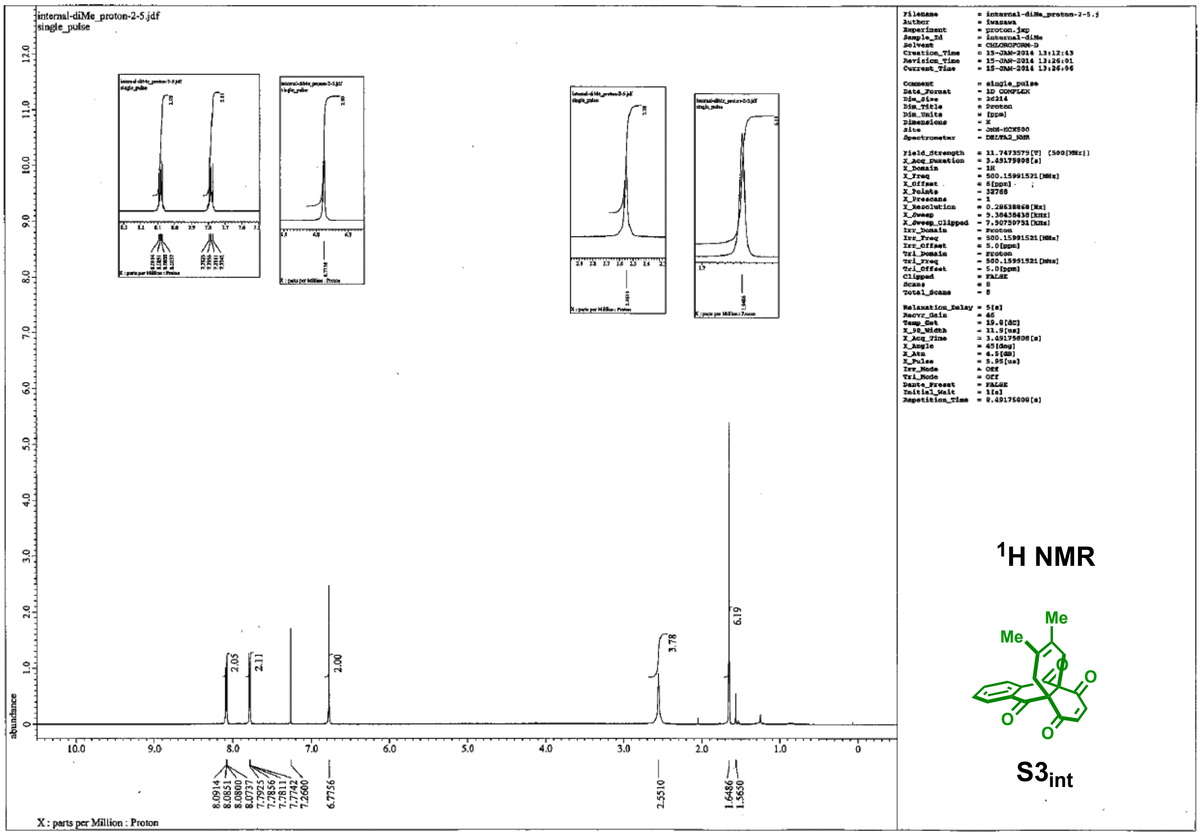
internal-diMe_proton-2-5.jdf
single_pulse
abundance
X : parts per Million : Proton
8.0914
8.0851
8.0800
8.0737
7.7925
7.7856
7.7811
7.7742
7.2600
6.7756
2.5510
1.6486
1.5650
2.05
2.11
2.00
3.78
6.19
^{1}H NMR
Me
Me
S3$_{int}$

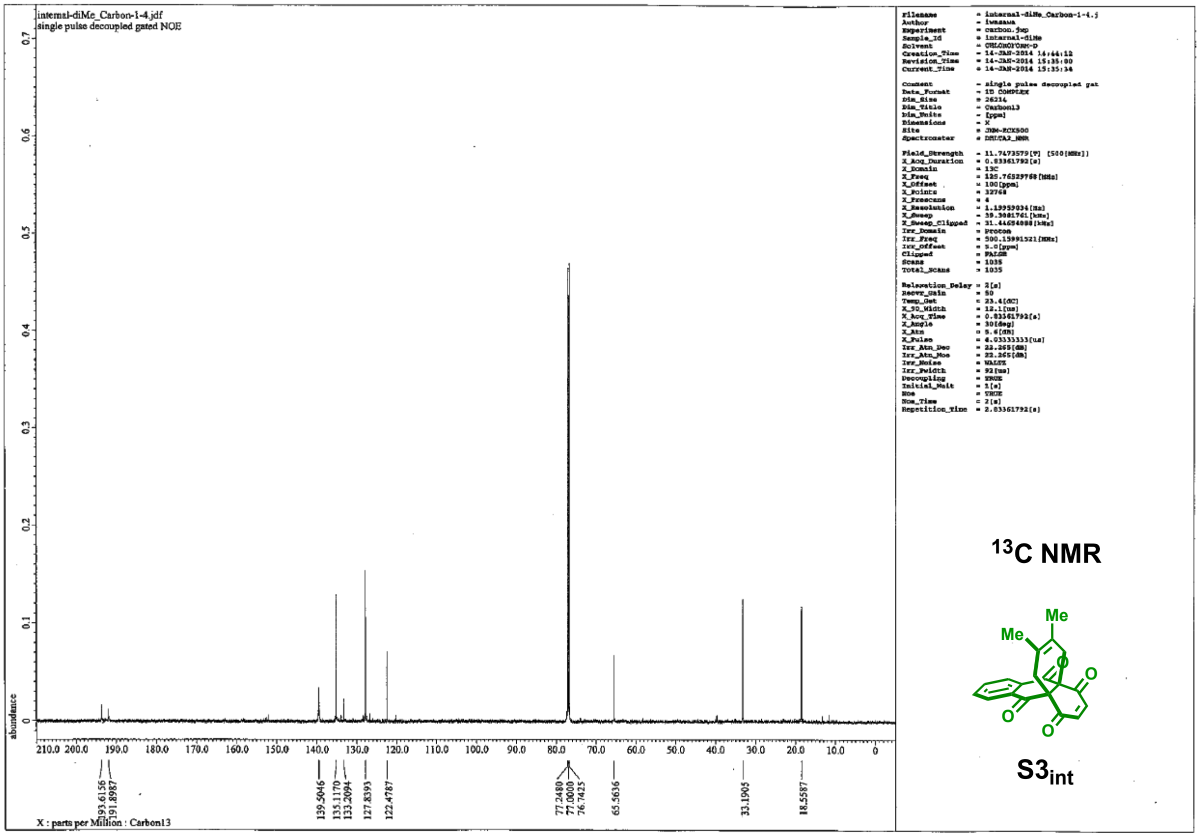
internal-diMe_Carbon-1-4.jdf
single pulse decoupled gated NOE
abundance
X : parts per Million : Carbon13
193.6156
191.8987
139.5046
135.1170
133.2094
127.8393
122.4787
77.2480
77.0000
76.7425
65.5636
33.1905
18.5587
^{13}C NMR
Me
Me
S3$_{int}$

Condition: Table 2 entry 7

(a)

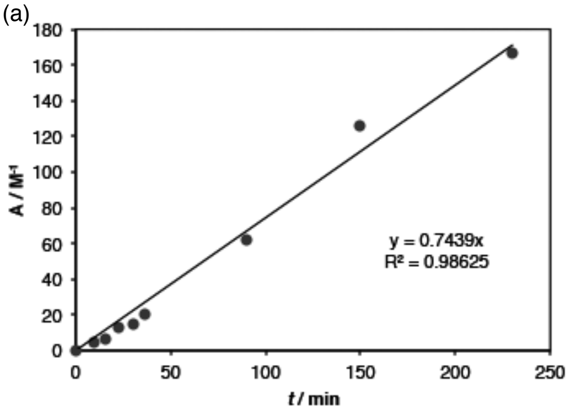

(b)

t/min	Conversion %	[1]	[9]	A/M^{-1}
0	0	0.00656	0.00971	0.0000
10	4	0.00630	0.00945	4.2633
16	6	0.00617	0.00932	6.5080
23	11	0.00584	0.00899	12.4831
30	13	0.00571	0.00886	15.0310
36	17	0.00545	0.00859	20.4274
90	40	0.00394	0.00709	62.1737
150	60	0.00263	0.00577	125.9169
230	68	0.00210	0.00525	166.5255

(c) $k = 0.74\ \mathrm{min^{-1} M^{-1}}$

Fig. S19 (a) Second-order plot (A / M^{-1} vs. t / min) (b) table of time dependence of conversion, [1], [9] and A and (c) reaction rate

Condition: Table 2 entry 8

(a)

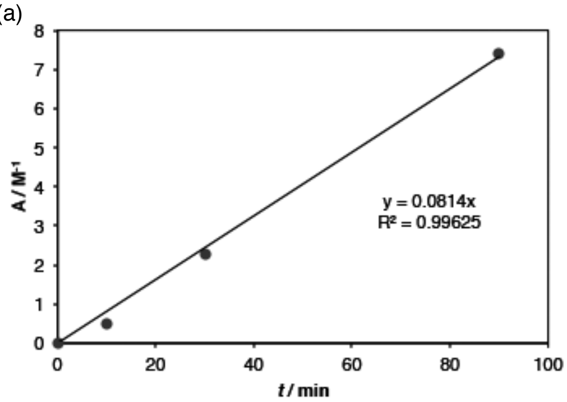

(b)

t/min	Conversion %	[1]	[9]	A/M^{-1}
0	0	0.01310	0.01960	0.0000
10	1	0.01297	0.01947	0.5125
30	4.3	0.01254	0.01904	2.2669
90	13	0.01140	0.01789	7.4129

(c) $k = 0.081\ \mathrm{min^{-1} M^{-1}}$

Fig. S20 (a) Second-order plot (A / M^{-1} vs. t / min) (b) table of time dependence of conversion, [1], [9] and A and (c) reaction rate

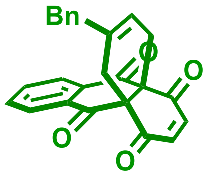

Physical data of $\mathbf{S4}_{\mathbf{int}}$

^{1}H NMR (500 MHz, $CDCl_3$): δ 8.09-8.05 (m, 2H), 7.79-7.75 (m, 2H), 7.28 (d, J = 7.6 Hz, 2H), 7.19 (t, J = 7.2 Hz, 1H), 7.11 (d, J = 7.6 Hz, 2H), 6.75 (m, 2H), 5.50 (br, 1H), 3.37-3.34 (m, 2H), 2.70 (br, 2H), 2.47 (br, 2H).

^{13}C NMR (125 MHz, $CDCl_3$): δ 193.4, 191.9, 139.4, 138.1, 135.2, 133.9, 133.2, 128.9, 128.4, 127.9, 126.4, 119.0, 65.5, 64.8, 43.5, 30.3, 27.8.

HRMS (FAB^+, NBA): m/z Calcd. for $C_{25}H_{18}O_4$: 382.1205., Found: 382.1213 $[M]^+$.

IR (ATR): 2893, 1710, 1681, 1592, 1494, 1453, 1423, 1258, 1234, 1070 cm^{-1}.

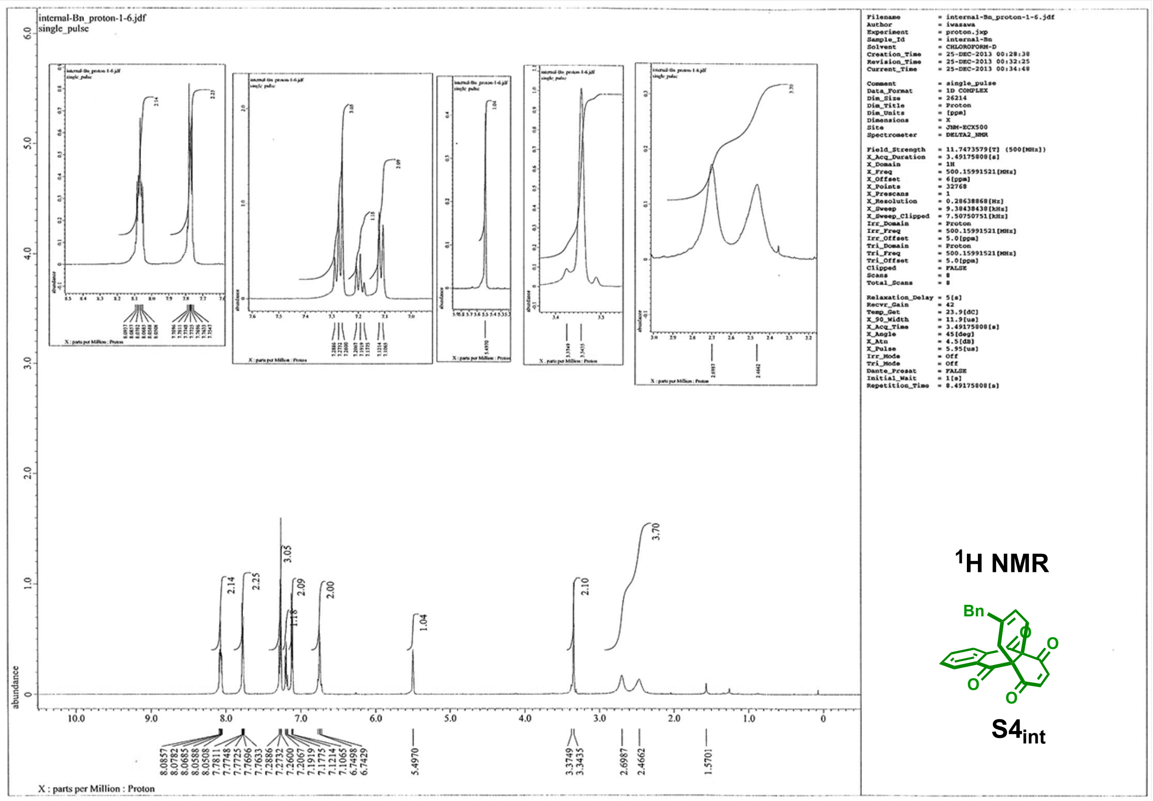

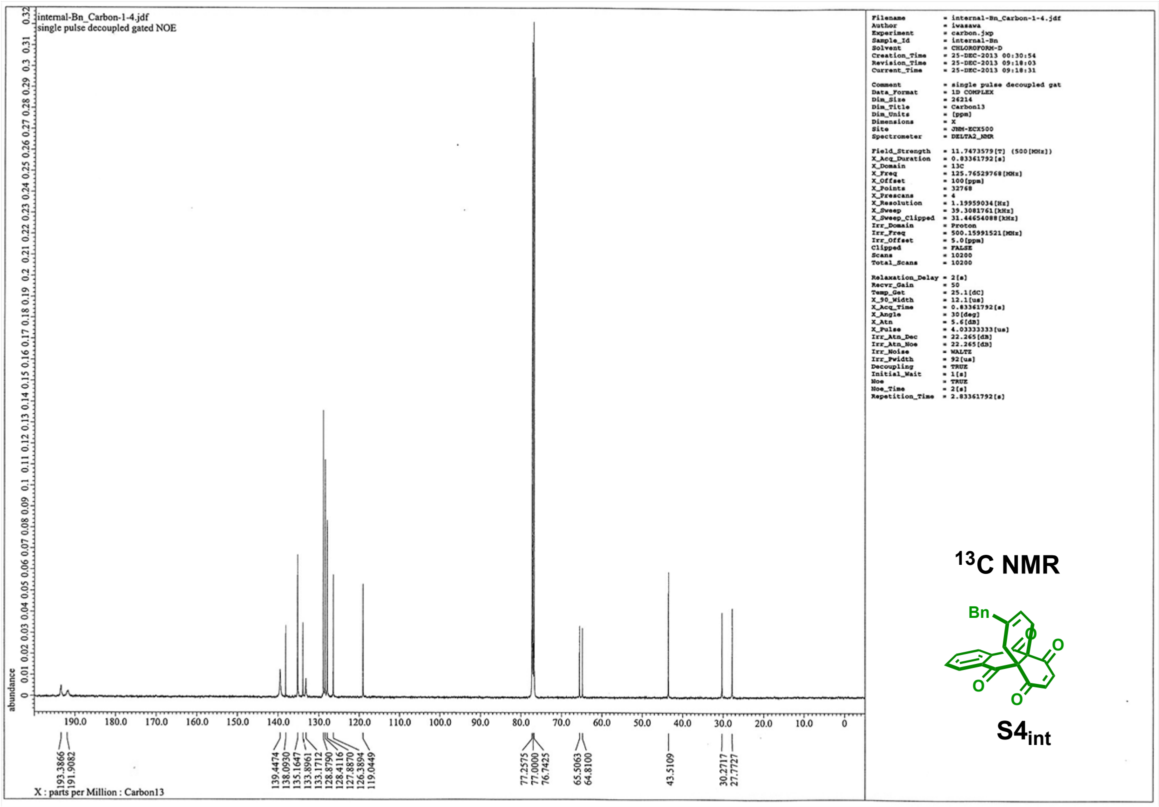

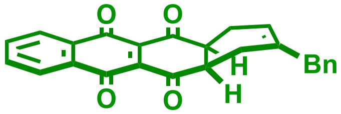

Physical data of **S4$_{ter}$**

^{1}H NMR (500 MHz, CDCl$_3$): δ 8.06-8.04 (m, 2H), 7.81-7.79 (m, 2H), 7.28 (d, *J* = 7.5 Hz, 2H), 7.18 (t, *J* = 7.5 Hz, 2H), 7.15 (d, *J* = 7.5 Hz, 1H), 5.52 (br, 1H), 3.45-3.52 (m, 2H), 2.05 (s, 2H), 2.51-2.54 (m, 1H), 2.30 (dd, *J* = 16.5, 4.9 Hz, 2H), 2.09 (dd, *J* = 16.5, 4.9 Hz, 1H).

^{13}C NMR (125 MHz, CDCl$_3$): δ 196.5, 196.4, 183.3, 183.3, 138.7, 134.8, 134.4, 133.6, 133.5, 131.8, 128.8, 128.4, 126.5, 126.3, 119.7, 49.7, 49.1, 43.9, 26.6, 24.1.

HRMS (FAB^{+}, NBA): *m/z* Calcd. for $C_{25}H_{20}O_4$: 384.1362., Found: 384.1359 [M+2H]$^{+}$. The peak derived from reduction product was observed in MS analysis.

IR (ATR): 1707, 1665, 1592, 1492, 1453, 1279, 1222, 1180 cm^{-1}.

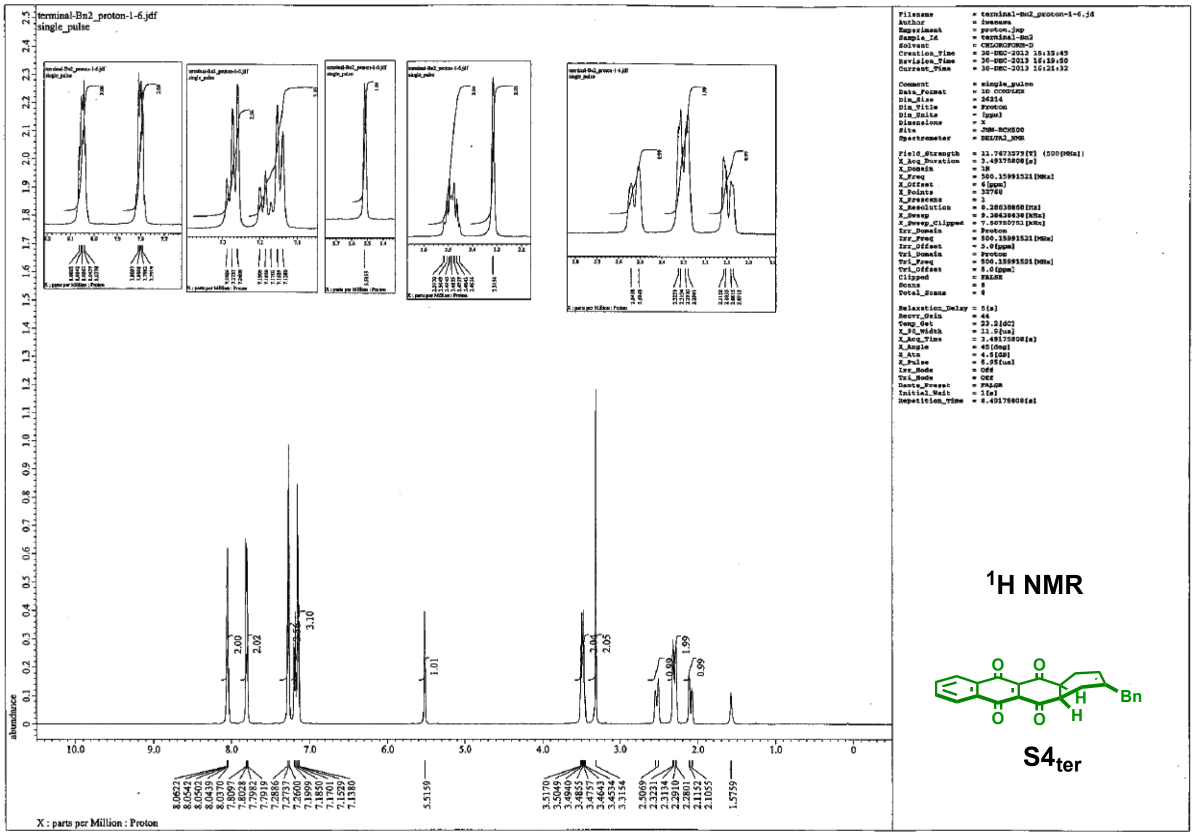

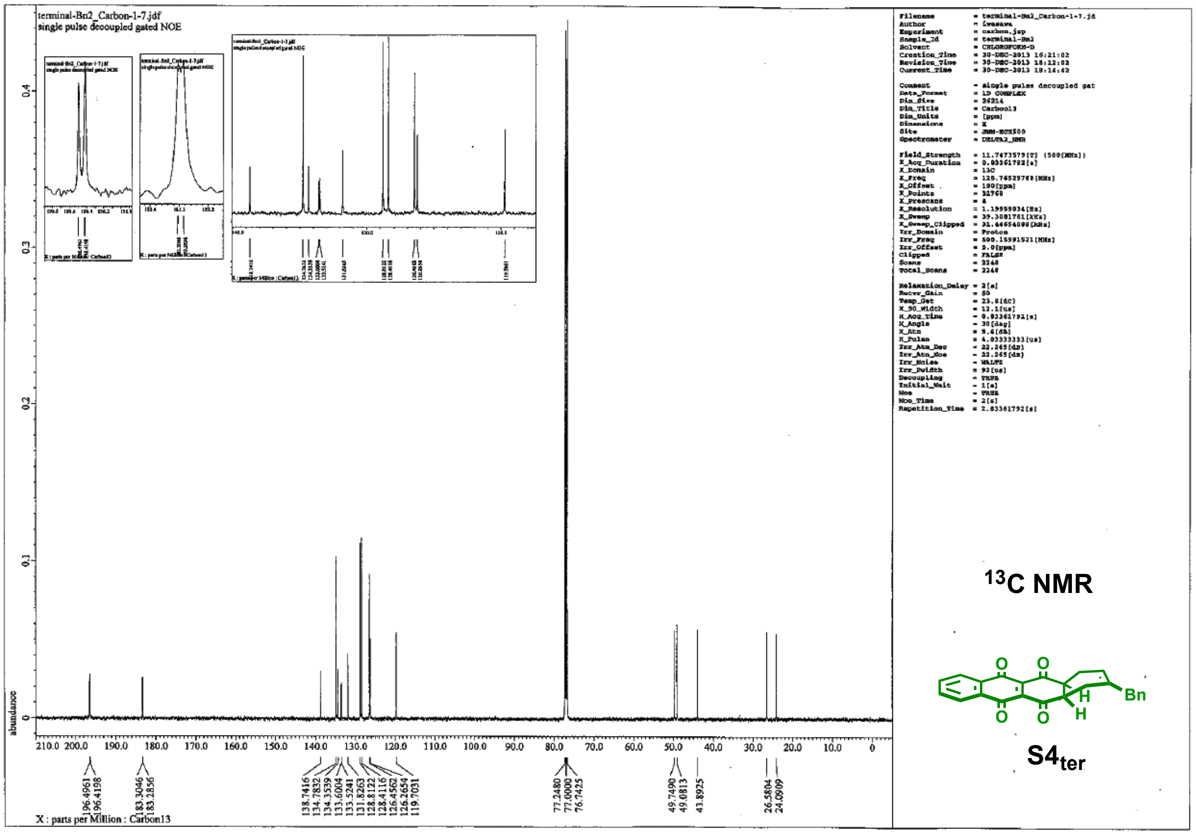

Condition: Table 2 entry 9

(a)

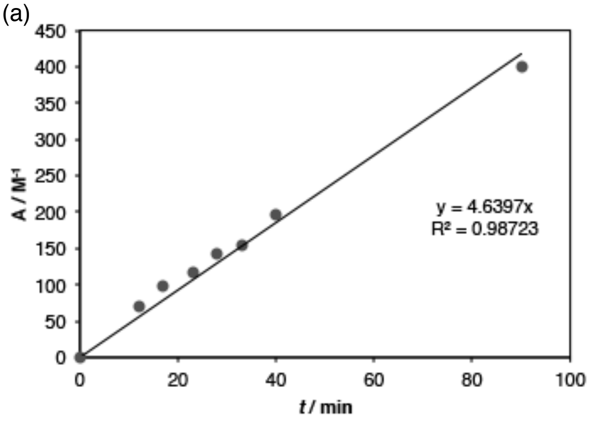

(b)

t/min	Conversion %	[1]	[10]	A/M⁻¹
0	0	0.00656	0.00879	0.0000
12	39.6	0.00396	0.00619	69.0239
17	49	0.00335	0.00557	97.8570
23	54	0.00302	0.00525	116.9502
28	59.5	0.00266	0.00489	142.1244
33	62	0.00249	0.00472	155.3970
40	68.5	0.00207	0.00429	197.1166
90	85	0.00098	0.00321	399.8263

(c) $k = 4.64\ \mathrm{min^{-1}\,M^{-1}}$

Fig. S21 (a) Second-order plot (A / M^{-1} vs. t / min) (b) table of time dependence of conversion, [1], [10] and A and (c) reaction rate

Condition: Table 2 entry 10

(a)

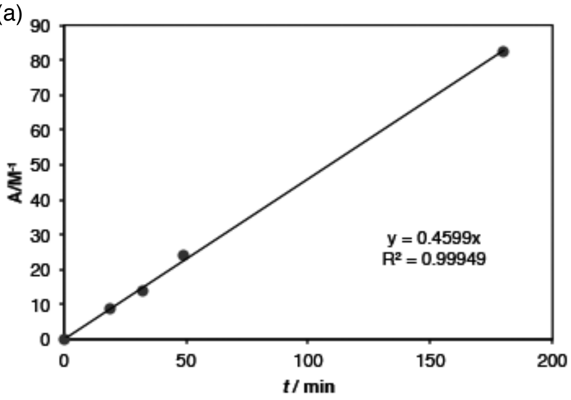

(b)

t/min	Conversion %	[1]	[10]	A/M⁻¹
0	0	0.01500	0.02250	0.0000
19	17	0.01245	0.01995	8.8058
32	25	0.01125	0.01875	14.0481
49	37	0.00945	0.01695	23.8384
180	72	0.00420	0.01170	82.5386

(c) $k = 0.46\ \mathrm{min}^{-1}\,\mathrm{M}^{-1}$

Fig. S22 (a) Second-order plot (A / M^{-1} vs. t / min) (b) table of time dependence of conversion, [1], [10] and A and (c) reaction rate

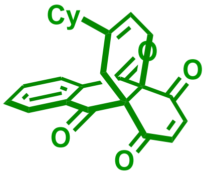

Physical data of **S5$_{int}$**

^{1}H NMR (500 MHz, $CDCl_3$): δ 8.09-8.07 (m, 2H), 7.82-7.77 (m, 2H), 6.77 (m, 2H), 5.40 (br, 1H), 2.66 (br, 2H), 2.55 (br, 2H), 1.85 (t, J = 14.0 Hz, 1H), 1.75-1.64 (m, 4H), 1.28-1.10 (m, 6H).

^{13}C NMR (125 MHz, $CDCl_3$): δ 193.6, 191.8, 139.6, 139.3, 135.1, 133.2, 127.9, 127.8, 114.9, 65.5, 65.5, 65.5, 64.9, 45.0, 31.4, 29.3, 27.7, 26.5, 26.2.

HRMS (FAB$^+$, NBA): m/z Calcd. for $C_{24}H_{23}O_4$: 375.1596., Found: 375.1605 $[M+H]^+$.

IR (ATR): 2925, 2851, 1711, 1683, 1593, 1448, 1424, 1258, 1238 cm^{-1}.

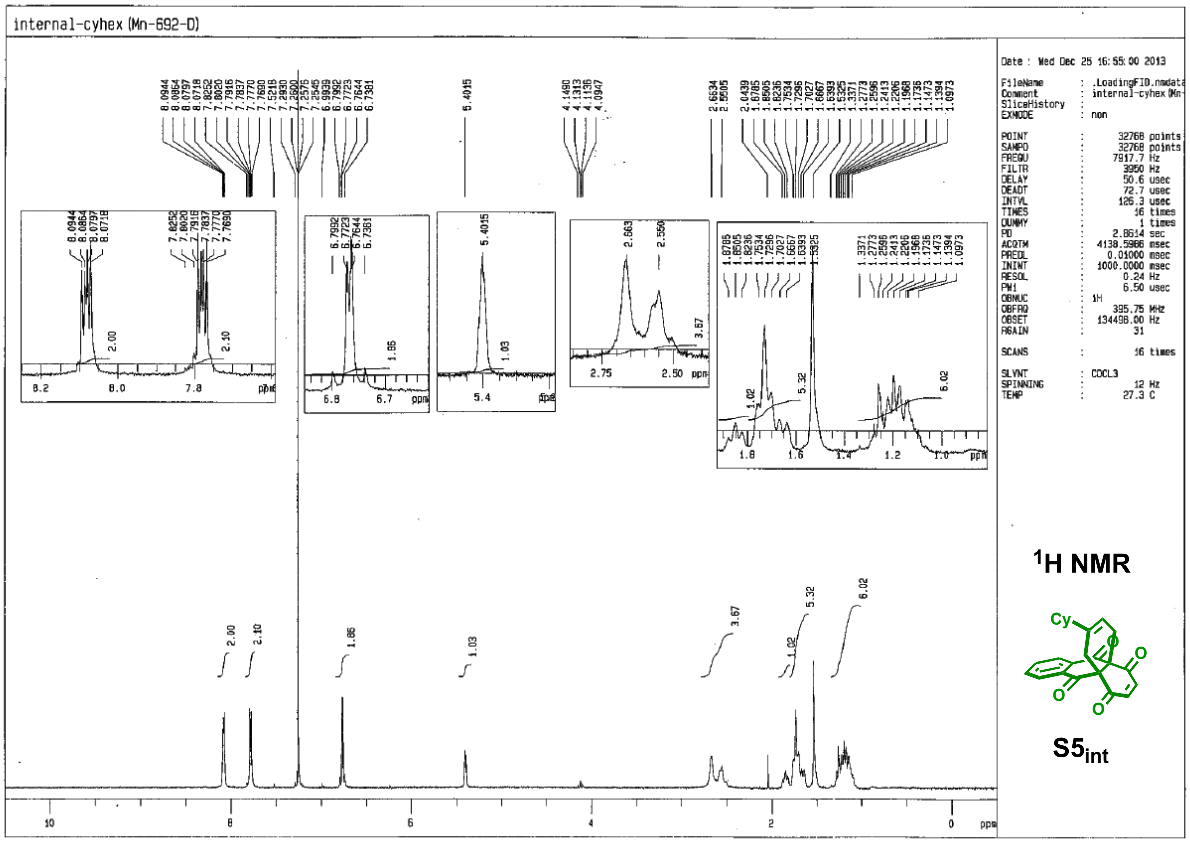
internal-cyhex (Mn-692-D)
Date : Wed Dec 25 16:55:00 2013
1H NMR
Cy
S5int

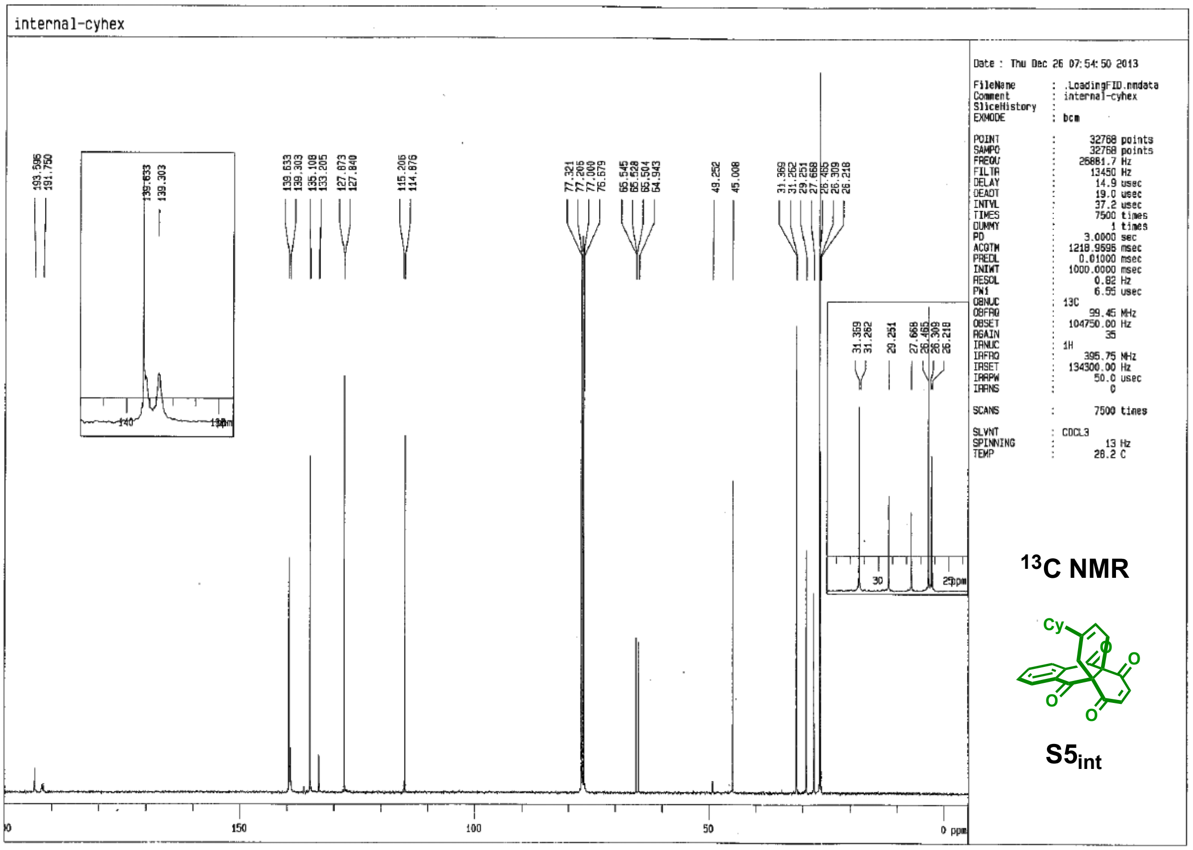
internal-cyhex
Date : Thu Dec 26 07:54:50 2013
13C NMR
Cy
S5int

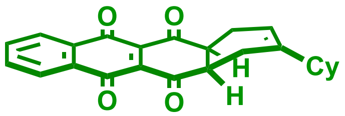

Physical data of **S5**$_{ter}$

^{1}H NMR (500 MHz, $CDCl_3$): δ 8.07-8.06 (m, 2H), 7.82-7.77 (m, 2H), 5.39 (br, 1H), 3.52-3.45 (m, 2H), 2.44 (dt, *J* = 18.6, 4.3 Hz, 2H), 2.27 (d, *J* = 18.6 Hz, 1H), 2.17 (dd, *J* = 18.6 Hz, 1H), 1.83 (t, *J* = 11.5 Hz, 1H), 1.76-1.65 (m, 5H), 1.29-1.09 (m, 5H).

^{13}C NMR (125 MHz, $CDCl_3$): δ 196.8, 196.6, 183.4, 183.4, 140.3, 134.8, 133.7, 133.4, 131.9, 126.5, 115.5, 49.9, 49.5, 45.4, 31.6, 31.5, 26.6, 26.3, 25.3, 24.0.

HRMS (FAB$^+$, NBA): *m/z* Calcd. for $C_{24}H_{24}O_4$: 375.1674., Found: 376.1646 [M+2H]$^+$. The peak derived from reduction product was observed in MS analysis.

IR (ATR): 1707, 1665, 1592, 1492, 1453, 1279, 1222, 1180 cm^{-1}.

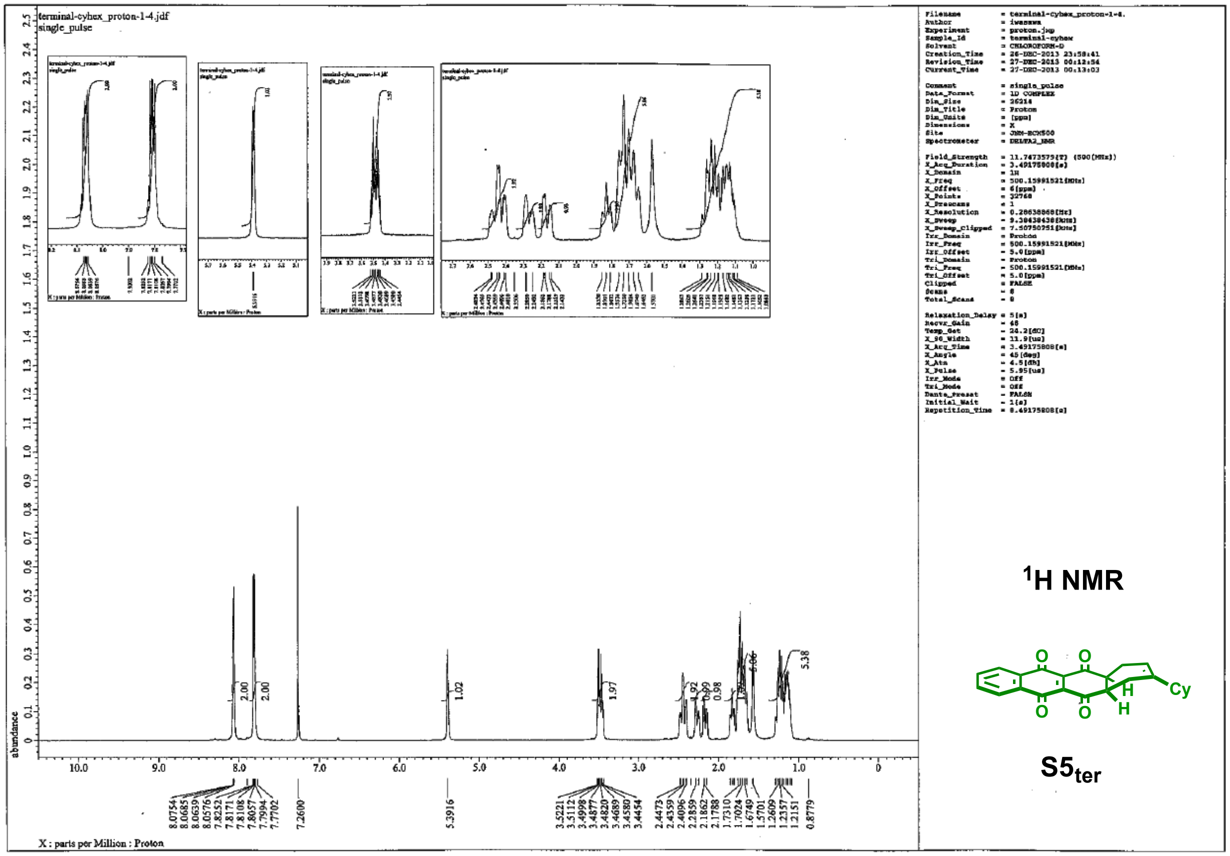

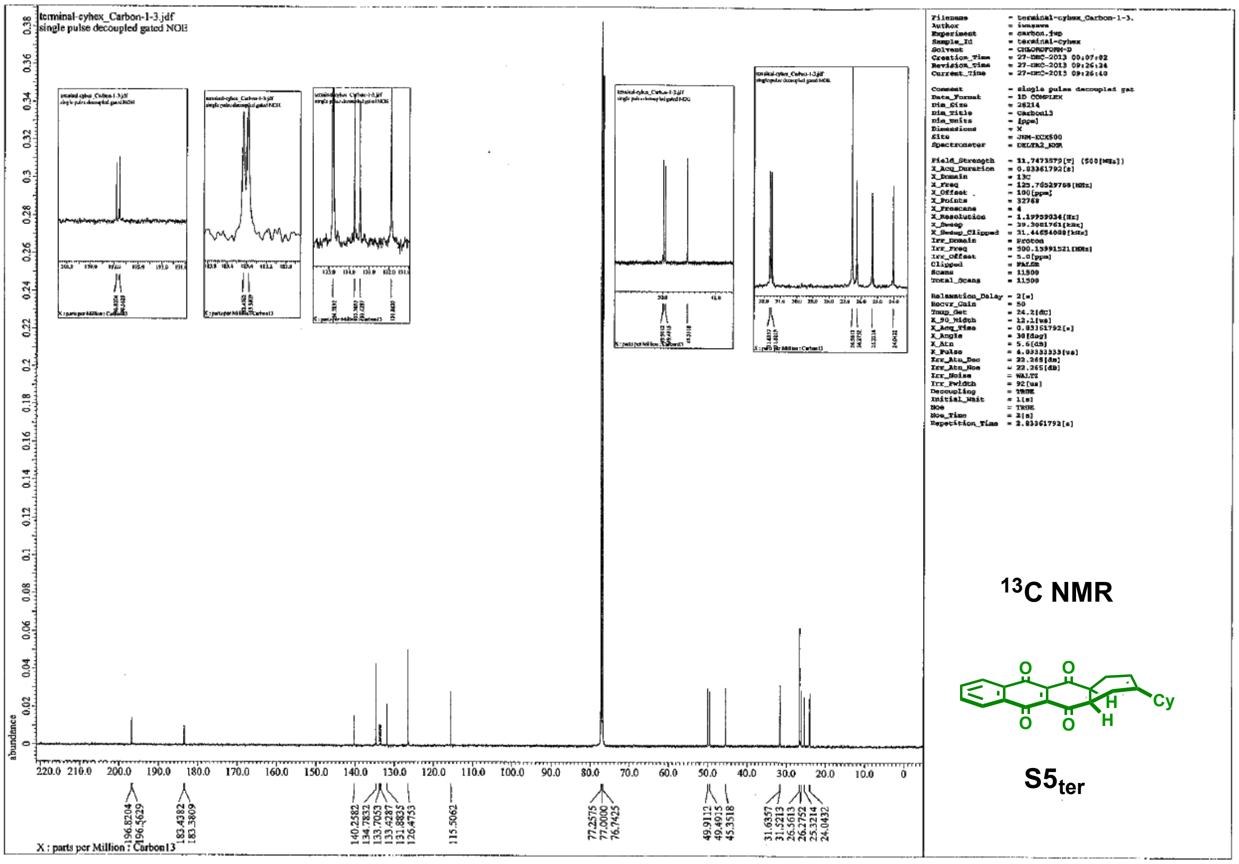

Condition: Table 2 entry 11

(a)

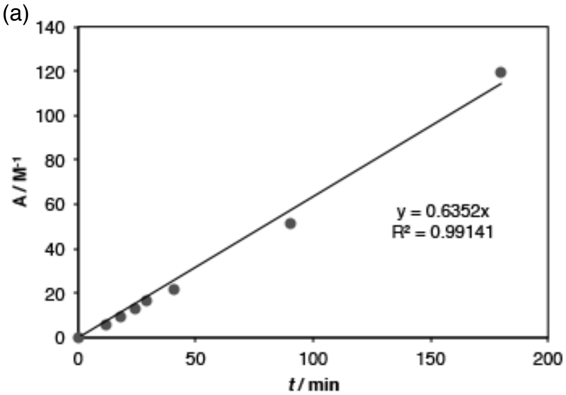

(b)

t/min	Conversion %	[1]	[11]	A/M^{-1}
0	0	0.00656	0.01037	0.0000
12	6	0.00617	0.00998	6.0853
18	9	0.00597	0.00978	9.3699
24	12	0.00578	0.00958	12.8336
29	15	0.00558	0.00939	16.4918
41	19	0.00532	0.00912	21.7023
90	37	0.00413	0.00794	51.2942
180	61	0.00256	0.00637	119.2066

(c) $k = 0.64\ \mathrm{min^{-1}\,M^{-1}}$

Fig. S23 (a) Second-order plot (A / M^{-1} vs. t / min) (b) table of time dependence of conversion, [1], [11] and A and (c) reaction rate

Condition: Table 2 entry 12

(a)

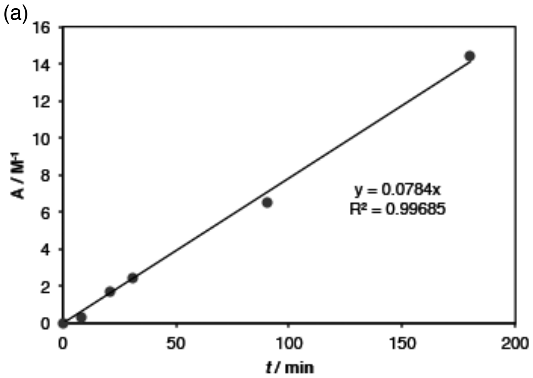

(b)

t/min	Conversion %	[1]	[11]	A/M⁻¹
0	0	0.01500	0.03000	0.0000
8	1	0.01485	0.02985	0.3359
21	5	0.01425	0.02925	1.7317
31	7	0.01395	0.02895	2.4629
90	17	0.01245	0.02745	6.4999
180	32.5	0.01013	0.02513	14.3806

(c) $k = 0.078\ \text{min}^{-1}\text{M}^{-1}$

Fig. S24 (a) Second-order plot (A / M^{-1} vs. t / min) (b) table of time dependence of conversion, [1], [11] and A and (c) reaction rate

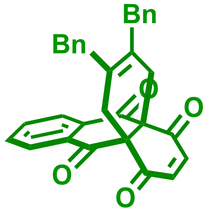

Physical data of **S6$_{\text{int}}$**

^{1}H NMR (500 MHz, $CDCl_3$): δ 8.05-8.04 (m, 2H), 7.77-7.74 (m, 2H), 7.28 (d, *J* = 7.5 Hz, 4H), 7.19 (t, *J* = 7.5 Hz, 2H), 7.12 (d, *J* = 7.5 Hz, 4H), 6.71 (br, 2H), 3.57 (q, *J* = 12.9 Hz, 4H), 2.59 (br, 4H).

^{13}C NMR (125 MHz, $CDCl_3$): δ 193.4, 191.7, 139.4, 138.5, 135.1, 133.1, 128.5, 128.4, 127.9, 127.7, 126.3, 65.4, 38.7, 31.7.

HRMS (FAB^{+}, NBA): *m/z* Calcd. for $C_{32}H_{24}O_4$: 472.1675., Found: 472.1699 $[M]^{+}$.

IR (ATR): 2920, 1710, 1684, 1593, 1494, 1260, 1074 cm^{-1}.

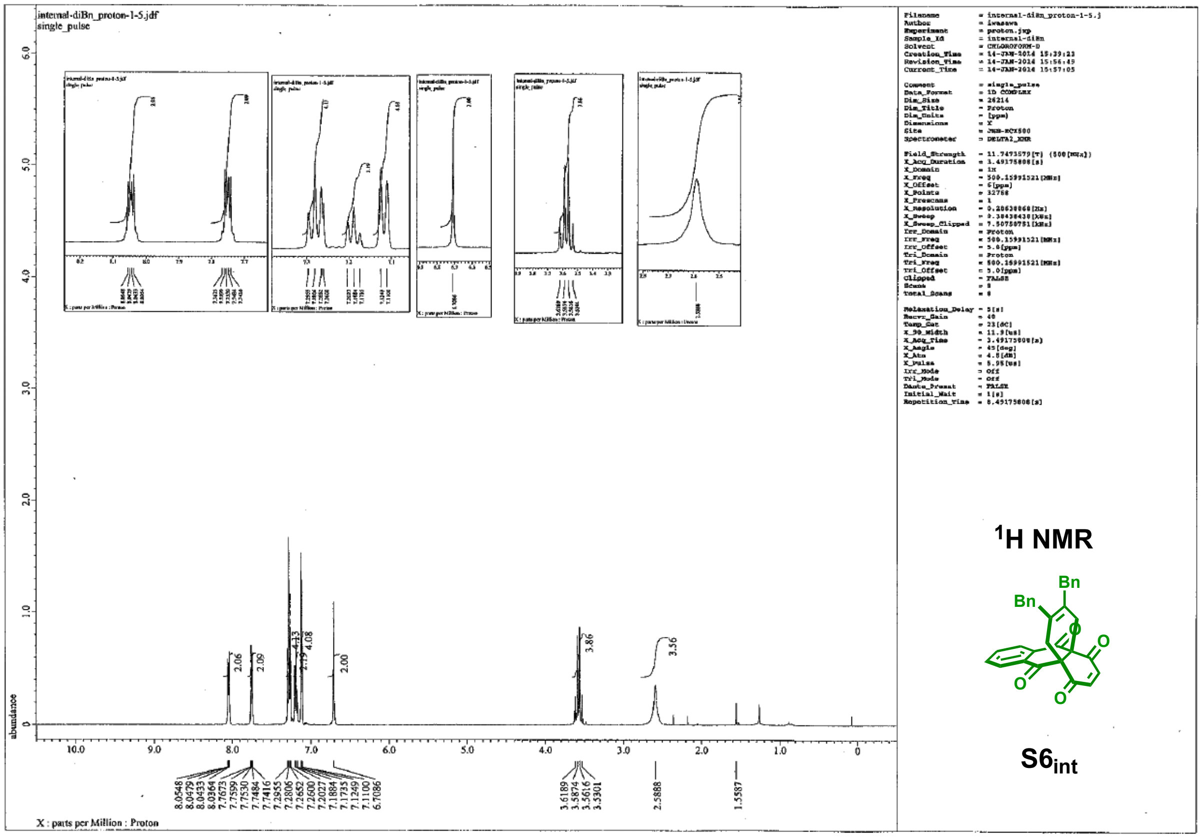
internal-diBn_proton-1-5.jdf
single_pulse
2.06
2.09
4.13
2.17
4.08
2.00
3.86
3.56
8.0548
8.0479
8.0433
8.0364
7.7673
7.7593
7.7530
7.7484
7.7416
7.2955
7.2806
7.2652
7.2600
7.2027
7.1884
7.1735
7.1249
7.1100
6.7086
3.6189
3.5874
3.5616
3.5301
2.5888
1.5587
X : parts per Million : Proton
abundance
¹H NMR
Bn
Bn
S6int

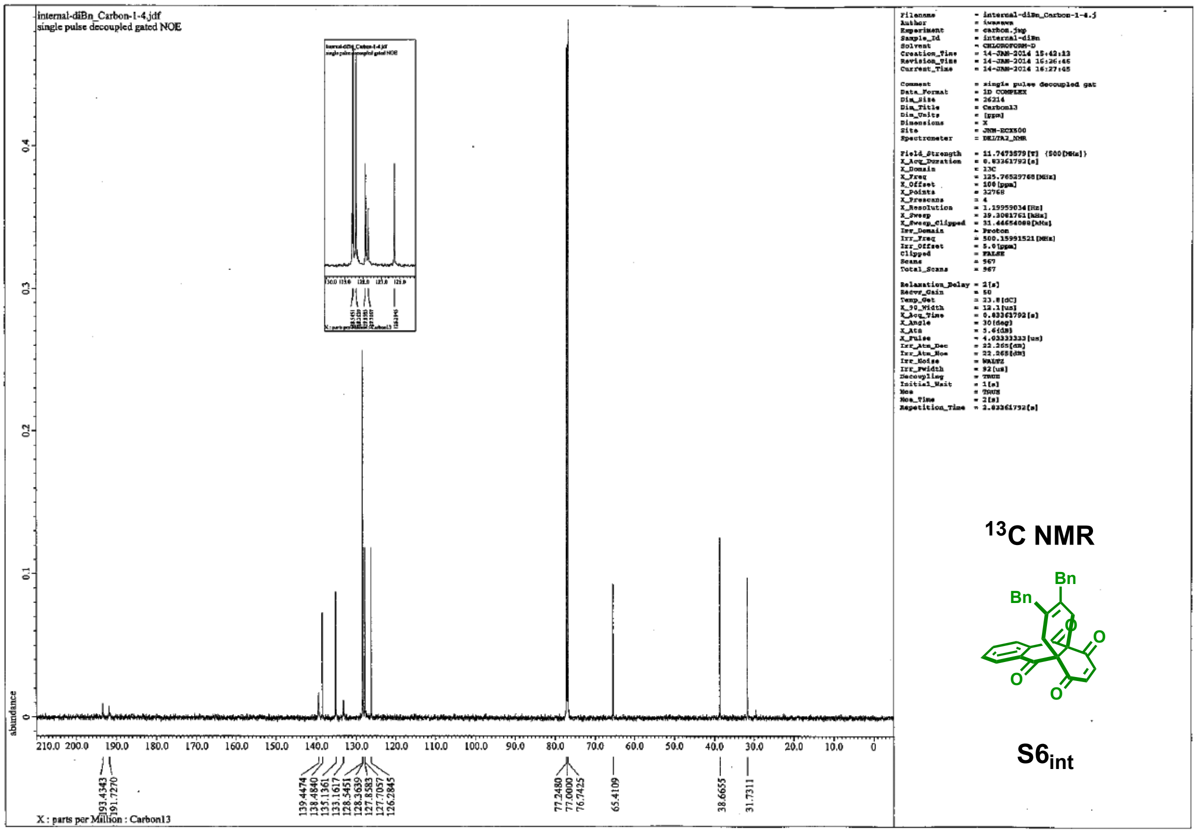
internal-diBn_Carbon-1-4.jdf
single pulse decoupled gated NOE
193.4343
191.7270
139.4474
138.4840
135.1561
133.1617
128.5451
128.3639
127.8583
127.7057
126.2845
77.2480
77.0000
76.7425
65.4109
38.6655
31.7311
X : parts per Million : Carbon13
abundance
¹³C NMR
Bn
Bn
S6int

ITC study revealed that the association constants of adducts **S1$_{int}$**, **S4$_{int}$** and **S6$_{int}$** were almost 0 M^{-1}. The binding behavior of **[2+2]$_{crown}$** containing K^+ with **S6$_{int}$** was also examined by ^{1}H NMR study. **[2+2]$_{crown}$** (2.0 mM), 2 equivalents of KOTf (4.0 mM), and 1 equivalent of **S6$_{int}$** (2.0 mM) were mixed in $CDCl_3/CD_3CN$ (0.8 mL, 1:1) and ^{1}H NMR spectrum was measured at 303 K. No obvious shift of proton signals of **S6$_{int}$** was observed, compared with the signal of free **S6$_{int}$** (Fig. S25a,b).

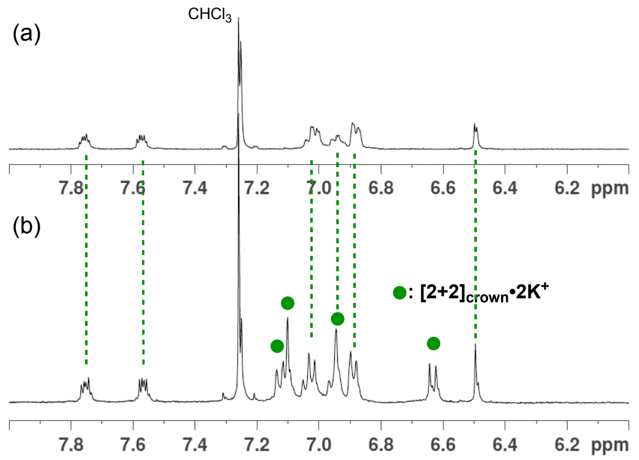

Fig. S25 Partial ^{1}H NMR spectra (400 MHz, $CDCl_3/CD_3CN$ (1:1)) of (a) S6$_{int}$ and (b) a mixture of [2+2]$_{crown}$ (2.0 mM), S6$_{int}$ (2.0 mM), and KOTf (4.0 mM).

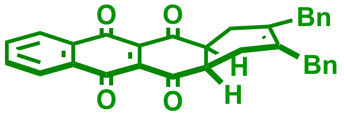

Physical data of **S6$_{ter}$**

^{1}H NMR (500 MHz, $CDCl_3$): δ 8.06-8.04 (m, 2H), 7.82-7.79 (m, 2H), 7.29 (t, J = 7.2 Hz, 4H), 7.16-7.20 (m, 6H), 3.60-3.51 (q, J = 14.3 Hz, 4H), 3.48 (t, J = 4.1 Hz, 2H), 2.43 (dd, J = 16.9, 4.1 Hz, 2H), 2.20 (dd, J = 18.0, 4.1 Hz, 2H).

^{13}C NMR (125 MHz, $CDCl_3$): δ 196.5, 183.3, 139.1, 134.8, 133.6, 131.8, 128.6, 128.4, 128.2, 126.5, 126.2, 49.8, 38.8, 28.3.

HRMS (FAB$^+$, NBA): *m/z* Calcd. for $C_{32}H_{26}O_4$: 474.1831., Found: 474.1815 $[M+2H]^+$. The peak derived from reduction product was observed in MS analysis.

IR (ATR): 1717, 1706, 1666, 1594, 1285 cm^{-1}.

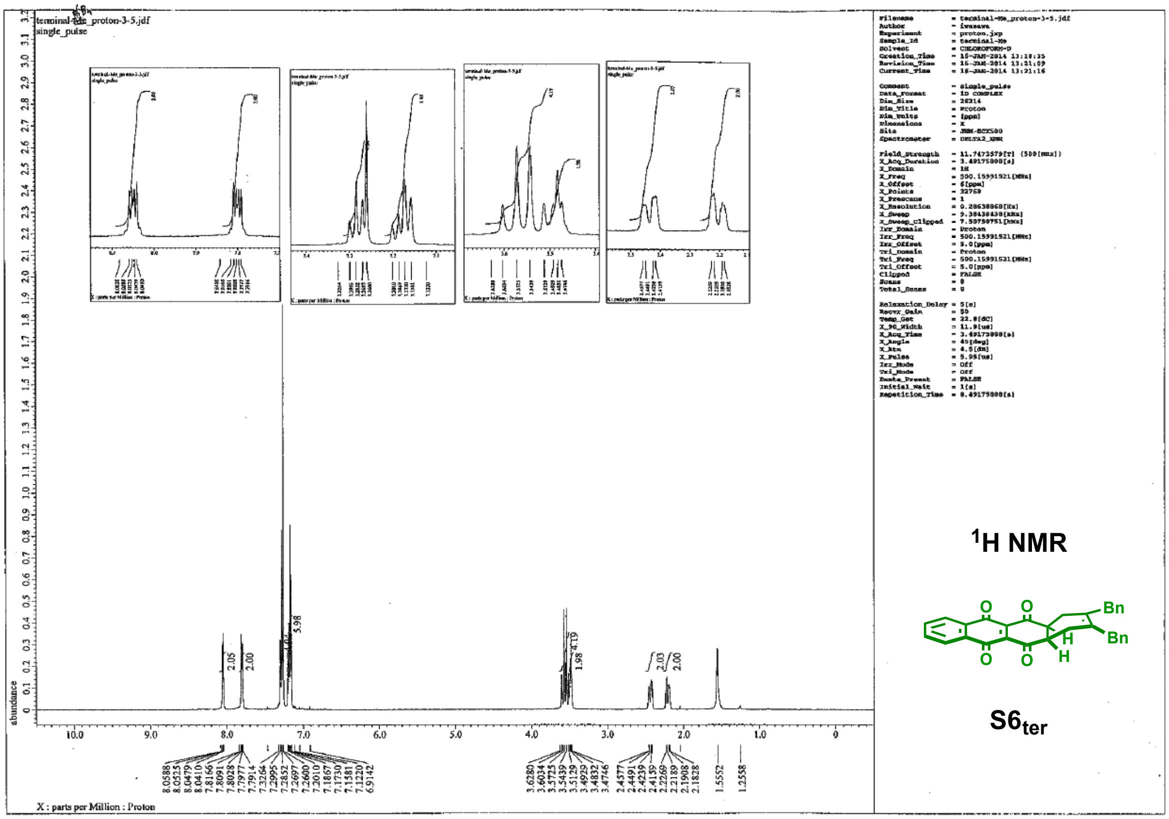
terminal-Me_proton-3-5.jdf
single_pulse
1H NMR
S6ter
Bn
Bn
H
H
2.05
2.00
5.98
4.19
1.98
2.03
2.00
8.0588 8.0525 8.0479 8.0410 7.8166 7.8091 7.8028 7.7977 7.7914 7.3264 7.2995 7.2852 7.2697 7.2600 7.2010 7.1867 7.1730 7.1581 7.1220 6.9142
3.6280 3.6034 3.5725 3.5439 3.5129 3.4929 3.4832 3.4746
2.4577 2.4491 2.4239 2.4159 2.2269 2.2189 2.1908 2.1828
1.5552
1.2558
X : parts per Million : Proton
abundance

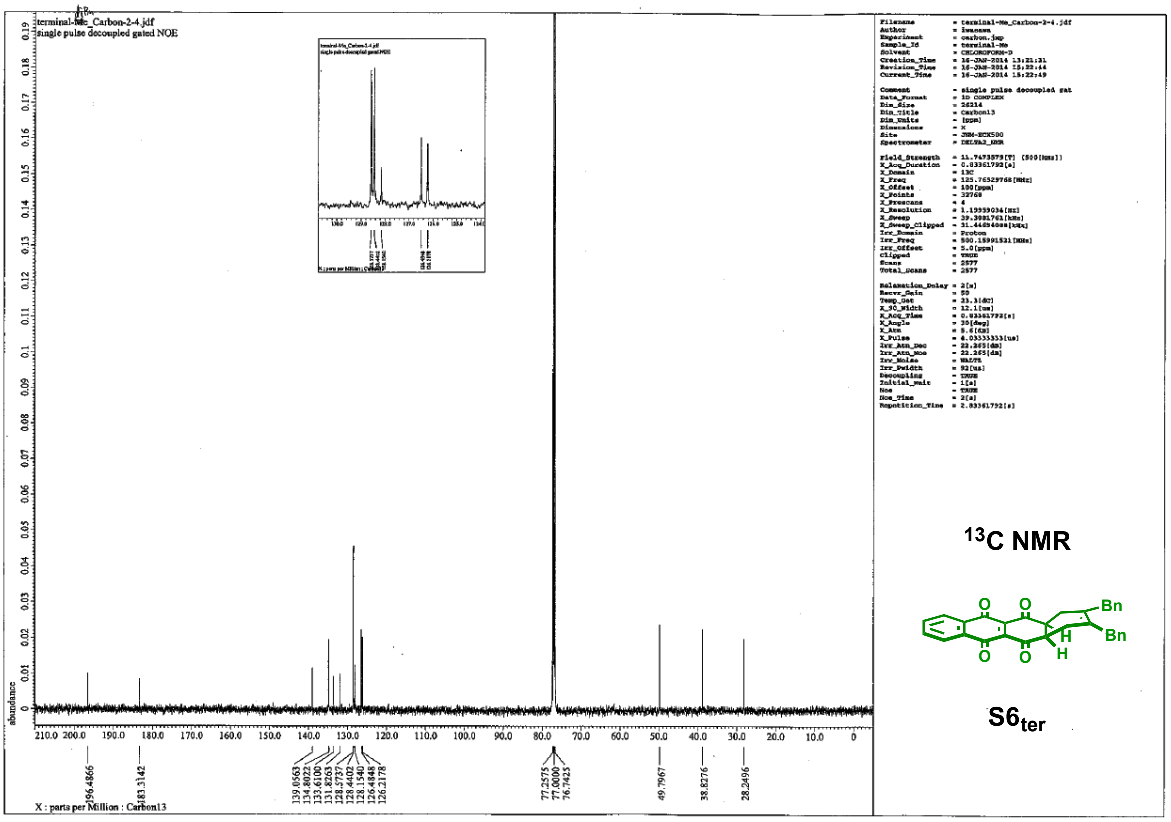
terminal-Me_Carbon-2-4.jdf
single pulse decoupled gated NOE
13C NMR
S6ter
Bn
Bn
H
196.4866
183.3142
139.0563 134.8022 133.6100 131.8263 128.5737 128.4402 128.1540 126.4848 126.2178
77.2575 77.0000 76.7425
49.7967
38.8276
28.2496
X : parts per Million : Carbon13
abundance

Condition: Table 2 entry 13

(a)

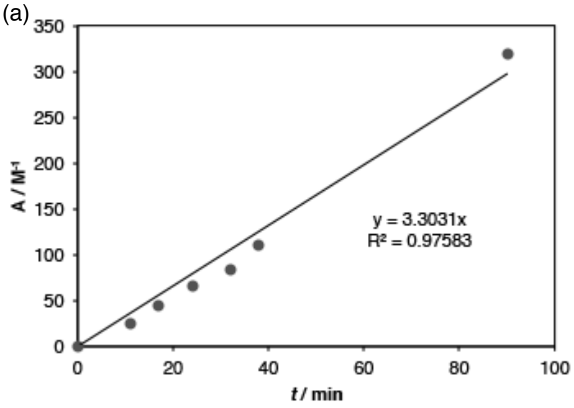

(b)

t/min	Conversion %	[1]	[12]	A/M^{-1}
0	0	0.00656	0.01050	0.0000
11	22	0.00512	0.00906	25.5388
17	34	0.00433	0.00827	44.8646
24	44	0.00368	0.00761	65.5947
32	51	0.00322	0.00715	83.7029
38	59	0.00269	0.00663	109.6171
90	87	0.00085	0.00479	318.9085

(c) $k = 3.30\ \mathrm{min}^{-1}\,\mathrm{M}^{-1}$

Fig. S26 (a) Second-order plot (A / M^{-1} vs. t / min) (b) table of time dependence of conversion, [1], [12] and A and (c) reaction rate

Condition: Table 2 entry 14

(a)

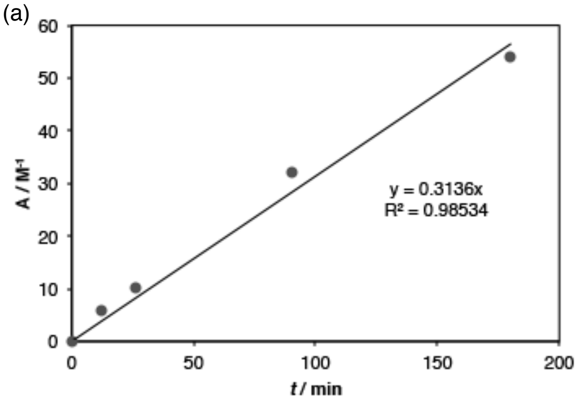

(b)

t/min	Conversion %	[1]	[12]	A/M^{-1}
0	0	0.01500	0.02250	0.0000
12	12	0.01320	0.02070	5.9269
26	19	0.01215	0.01965	10.0377
90	45	0.00825	0.01575	32.1549
180	60	0.00600	0.01350	54.0620

(c) $k = 0.32\ \mathrm{min}^{-1}\,\mathrm{M}^{-1}$

Fig. S27 (a) Second-order plot (A / M^{-1} vs. t / min) (b) table of time dependence of conversion, [1], [12] and A and (c) reaction rate

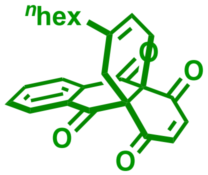

Physical data of **S7$_{\mathrm{int}}$**

^{1}H NMR (500 MHz, $CDCl_3$): δ 8.10-8.07 (m, 2H), 7.80-7.77 (m, 2H), 6.77 (br, 2H), 5.42 (br, 1H), 2.66 (br, 2H), 2.53 (br, 2H), 1.98 (t, J = 7.45 Hz, 2H), 1.39-1.38 (m, 2H), 1.25-1.23 (m, 6H), 0.87 (t, J = 7.2 Hz, 3H).

^{13}C NMR (125 MHz, $CDCl_3$): δ 193.6, 192.0, 139.4, 135.2, 134.8, 133.2, 127.9, 116.5, 65.5, 64.8, 36.9, 31.6, 30.4, 28.8, 27.7, 27.1, 22.5, 14.1.

HRMS (FAB$^+$, NBA): m/z Calcd. for $C_{24}H_{24}O_4$: 376.1675., Found: 376.1648 $[M]^+$.

IR (ATR): 2926, 2856, 1712, 1679, 1592, 1466, 157, 1427, 1365, 1257, 1084, 1067 cm^{-1}.

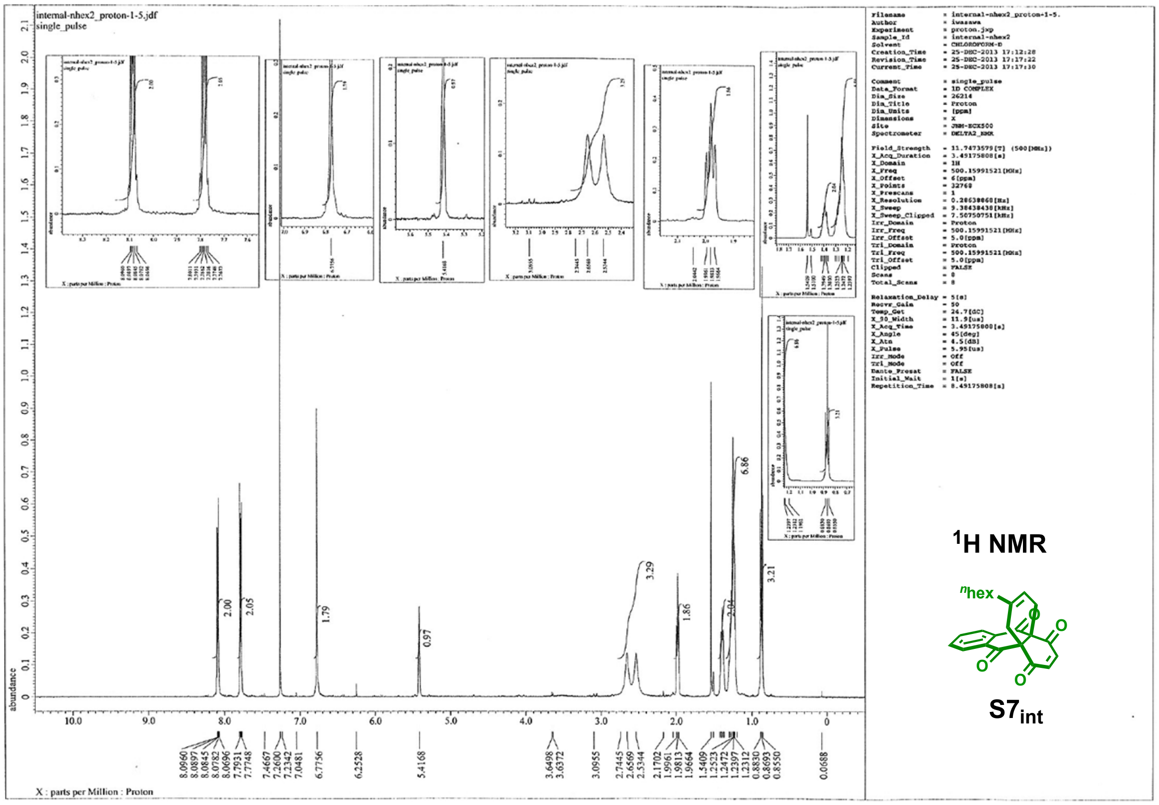
internal-nhex2_proton-1-5.jdf
single_pulse
X : parts per Million : Proton
1H NMR
nhex
S7int

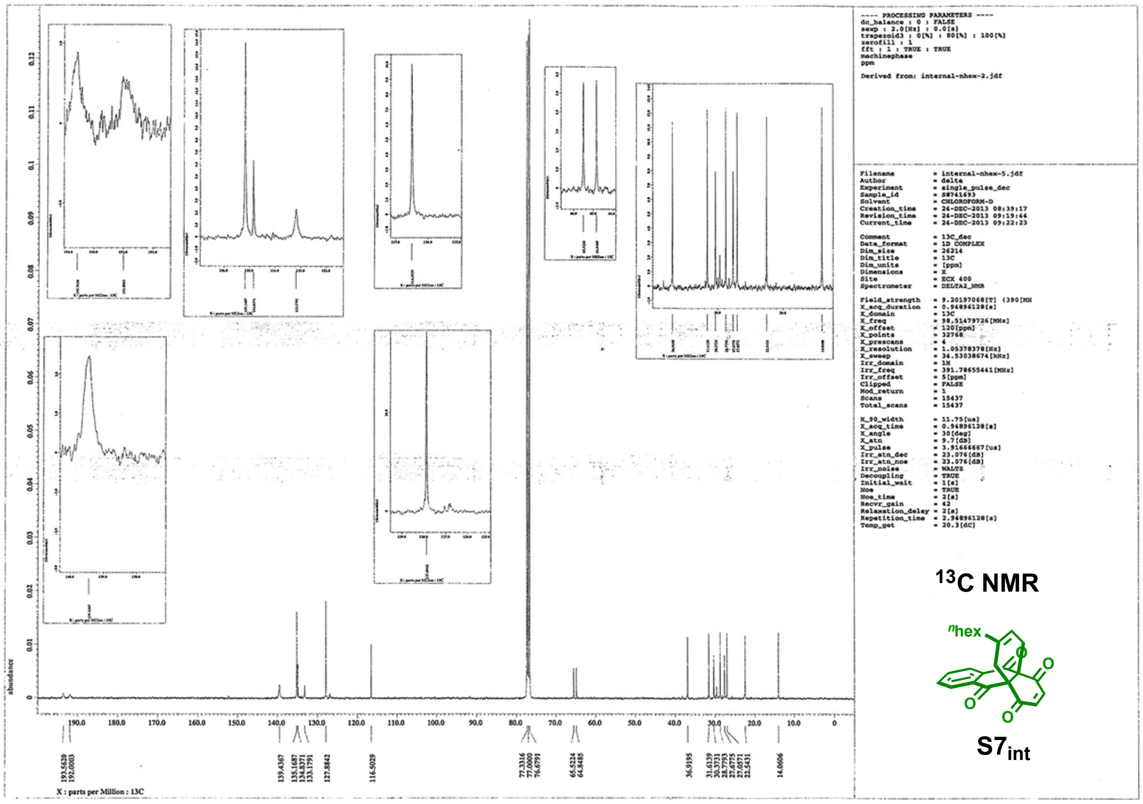
X : parts per Million : 13C
13C NMR
nhex
S7int

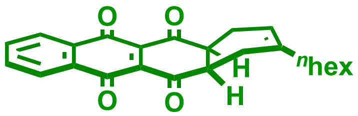

Physical data of **S7$_{ter}$**

^{1}H NMR (500 MHz, CDCl$_3$): δ 8.08-8.05 (m, 2H), 7.83-7.80 (m, 2H), 5.40 (br, 1H), 3.53 (q, J = 5.5 Hz, 1H), 3.45 (q, J = 5.5 Hz, 1H), 2.47-2.38 (m, 2H), 2.28-2.24 (m, 1H), 2.16-2.12 (m, 1H), 1.98 (t, J = 7.8 Hz, 2H), 1.39-1.38 (m, 2H), 1.31-1.26 (m, 6H), 0.87 (t, J = 7.2 Hz, 3H).

^{13}C NMR (125 MHz, CDCl$_3$): δ 196.8, 196.5, 183.4, 183.4, 135.3, 134.8, 133.6, 133.6, 131.9, 126.5, 117.2, 49.8, 49.3, 37.3, 31.7, 28.9, 27.4, 26.7, 24.1, 22.6, 14.1.

HRMS (FAB$^+$, NBA): m/z Calcd. for $C_{24}H_{26}O_4$: 378.1831., Found: 378.1856 [M+2H]$^+$.

IR (ATR): 1717, 1706, 1668, 1593, 1287 cm^{-1}.

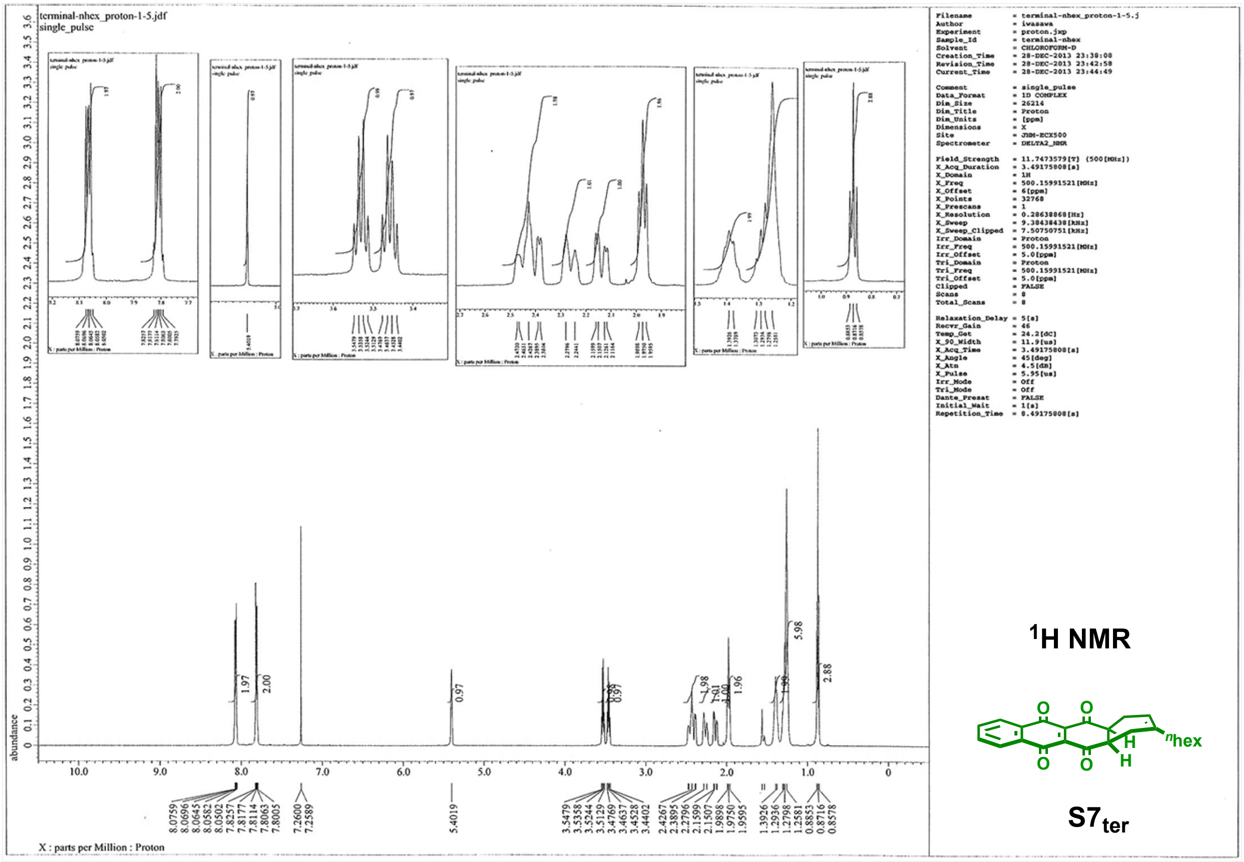

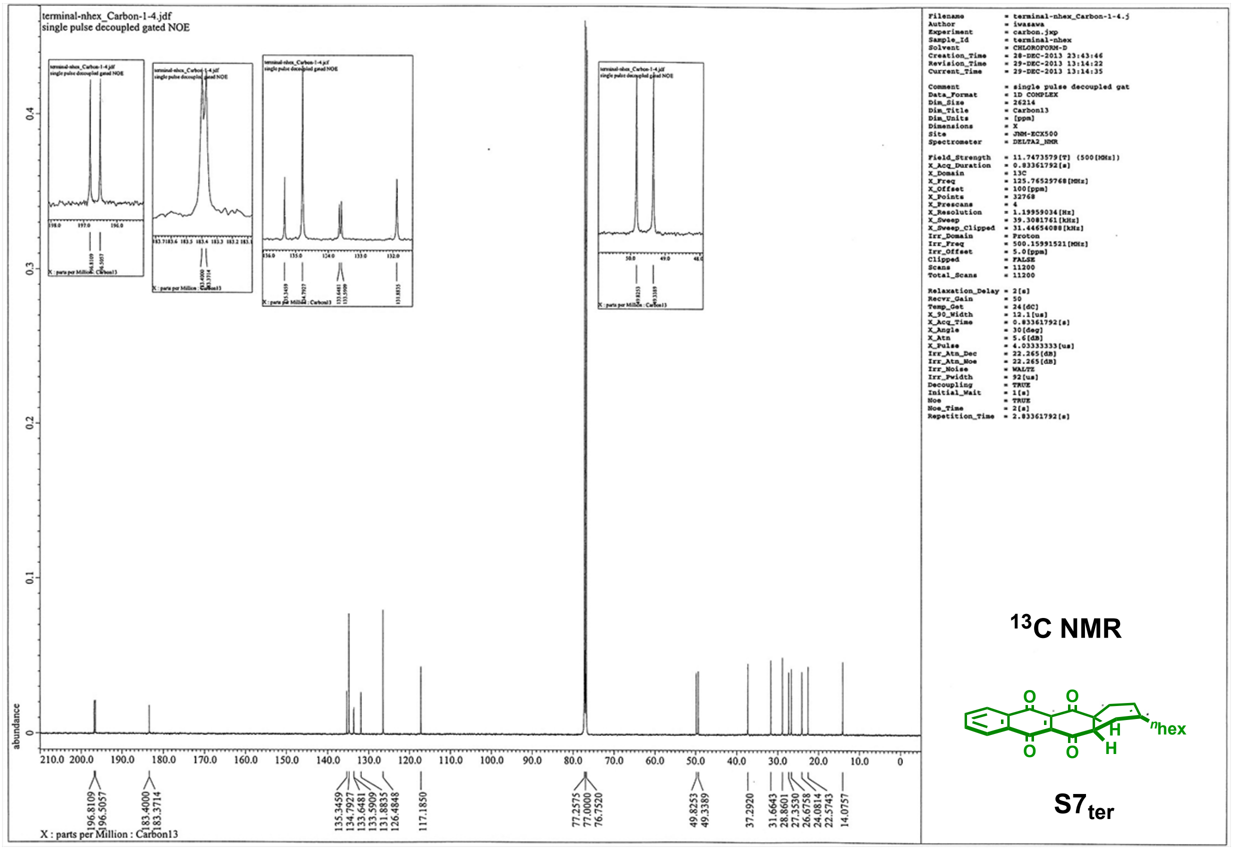

4-2-2. The Enantioselective Diels-Alder Reaction of **1** with 2-Substituted 1,3-Butadienes

The enantioselectivities of the reaction of **1** and various 2-substituted 1,3-butadienes in the presence of chiral host catalyst **[2+2]**$_{crown}$**•2K**$^{+}$ were examined (Table S1). Chirality induction was slightly observed (up to 19% ee). It also suggested that **[2+2]**$_{\mathbf{crown}}$ framework recognized the substituent of dienes and the reaction proceeded inside the host.

Typical experiment: **1** (10.5 μmol), 2-substituted 1,3-butadiene, **[2+2]**$_{crown}$ (5.25 μmol), and KOTf (10.5 μmol) were dissolved in 0.8 mL of $CHCl_3$/CH_3CN (1:1) in a test tube and the solution was stirred at -10 ºC for 3h. The reaction mixture was evaporated, dissolved in dichloromethane and washed with water. The organic layer was concentrated under reduced pressure, followed by purification by GPC to afford the product. The enantiomeric excess (*ee)* of the product was determined by HPLC using Chiralpak IF column (DAICEL, 4.6 mmφ x 250 mm) [EtOAc/hexane (20:80)]; flow rate 1.0 mL/min, UV detector 254 nm (Fig. S28).

Table S1 The enantioselective Diels-Alder reaction of 1 with 2-substituted 1,3-butadines in the presence of [2+2]$_{\mathbf{crown}}$**•2K**$^{+}$

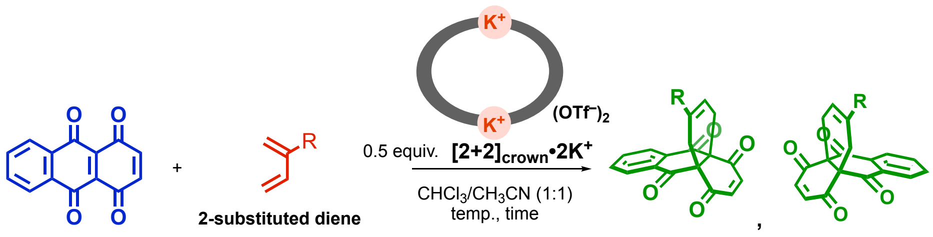

entry		diene	temp.	time	ee %
1	3 equiv.	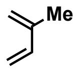 **6**	rt	3 h	11% ee
2			-10 °C	3 h	15% ee
3			-40 °C	3 h	19% ee
4[a]			rt	3 h	12% ee
5[b]			rt	24 h	11% ee
6	2.5 equiv.	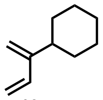 **10**	-10 °C	3 h	16% ee
7[c]			-10 °C	3 h	15% ee
8	1.5 equiv.		-40 °C	3 h	19% ee
9	2.5 equiv.	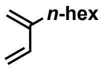 **12**	-10 °C	3 h	9% ee
10	2.5 equiv.	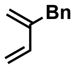 **9**	-10 °C	3 h	1% ee
11	2.5 equiv.	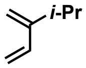 **7**	-10 °C	3 h	0.5% ee

[a] KPF_6 was employed. [b] Solvent was CH_3CN only. [c] 1 equiv. **[2+2]crown•2K+** and 2 equiv. KOTf were employed.

entry 1

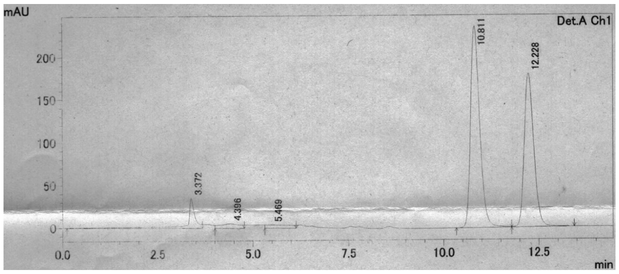

entry2

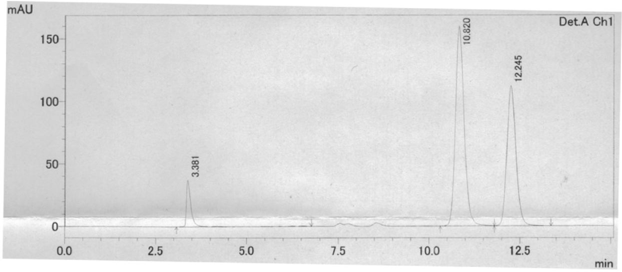

entry 3

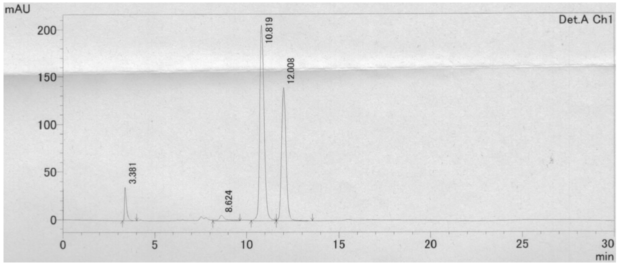

entry 4

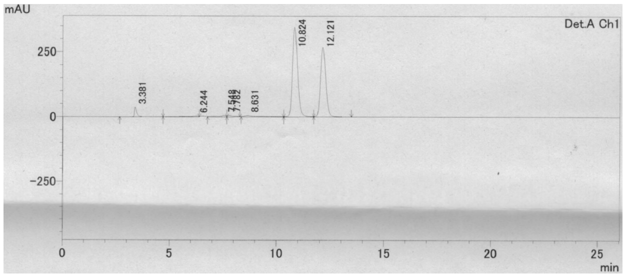

entry 5

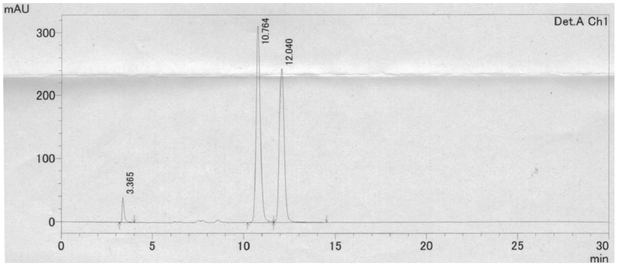

entry 6

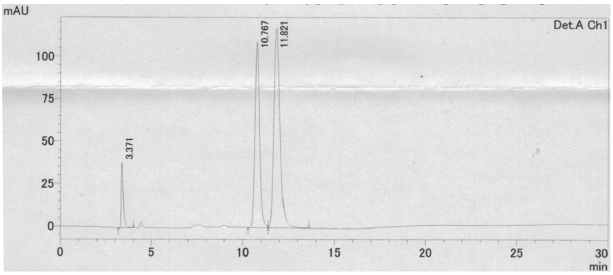

entry 7

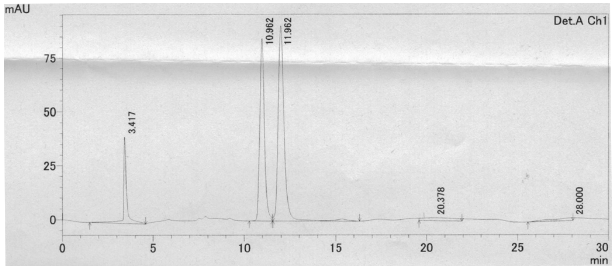

entry 8

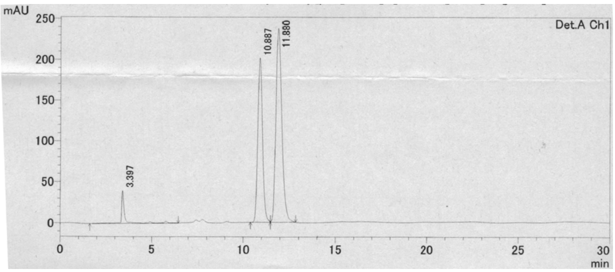

entry 9

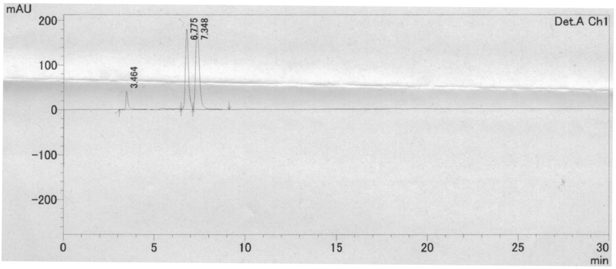

entry 10

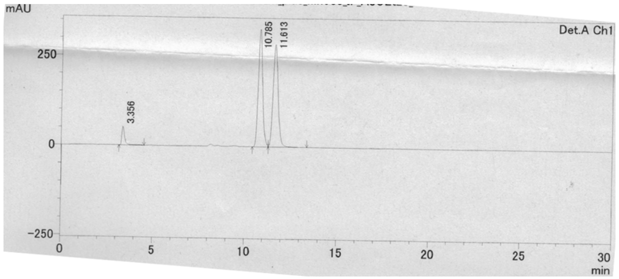

entry 11

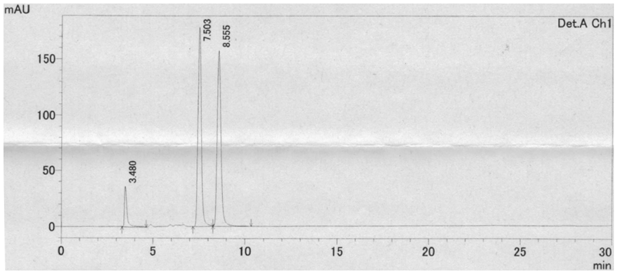

Fig. S28 HPLC charts

4-3. The Diels-Alder Reaction of **1** with 1-Mono and 1,4-Di-substituted 1,3-Butadienes

The limitation of applicable diene in this system was also assessed with 1-mono, and 1,4-di-substituted 1,3-butadienes **S8**, **S9**, and **S10** (Table S1). In the reaction with these dienes, the acceleration of the reaction was not observed clearly, and terminal adduct was obtained as major product regardless of whether the catalyst was used. This result indicates that substituents at 1- or 1,4-position of 1,3-butadienes are too much steric hindrance for this reaction.

Table S2 The Diels-Alder reaction of 1 and S8, S9 or S10 in the presence or absence of $[2+2]_{crown}$•$2K^+$ [a]

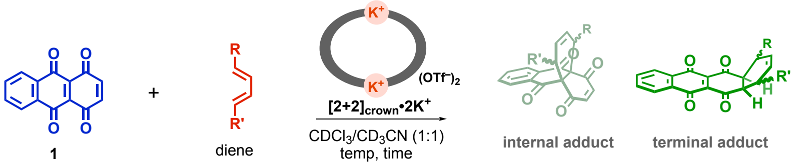

Entry		Diene	$[2+2]_{crown}$•$2K^+$	Temp.	Time	Yield[b] Internal	Terminal
1	1.5 equiv.	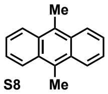 S8	1.0 equiv.	–10 °C	4 h	none	45%
2			none	–10 °C	4 h	none	34%
3	1.5 equiv.	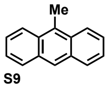 S9	1.0 equiv.	27 °C	1.5 h	none	68%
4			none	27 °C	1.5 h	trace	61%
5	3.0 equiv.	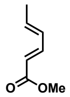 S10	1.0 equiv.	60 °C	4 h	none	44%
6			none	60 °C	4 h	trace	14%

[a] Reaction conditions: [**1**], 6.5 mM; [KOTf], 13 mM; 0.8 mL $CDCl_3/CD_3CN$ (1:1). [b] NMR yield.

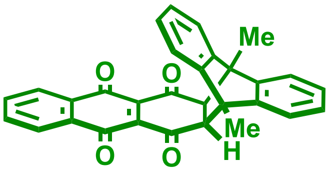

Physical data of **$S8_{ter}$**

^{1}H NMR (500 MHz, $CDCl_3$): δ 7.93 (br, 2H), 7.75 (br, 2H), 7.44 (br, 2H), 7.29 (br, 2H), 7.12 (br, 2H), 6.77 (br, 2H), 3.04 (s, 2H), 2.09 (s, 6H).

^{13}C NMR (125 MHz, $CDCl_3$): δ 194.6, 181.2, 143.7, 141.6, 136.1, 134.5, 130.9, 127.2, 126.8, 126.3, 123.1, 121.2, 60.0, 44.8, 15.9.

HRMS (FAB$^+$, NBA): *m/z* Calcd. for $C_{30}H_{21}O_4$: 445.1440, Found: 445.1460 $[M+H]^+$.

IR (ATR): 1710, 1661, 1590, 1454, 1386, 1291, 1272, 1191, 908 cm^{-1}.

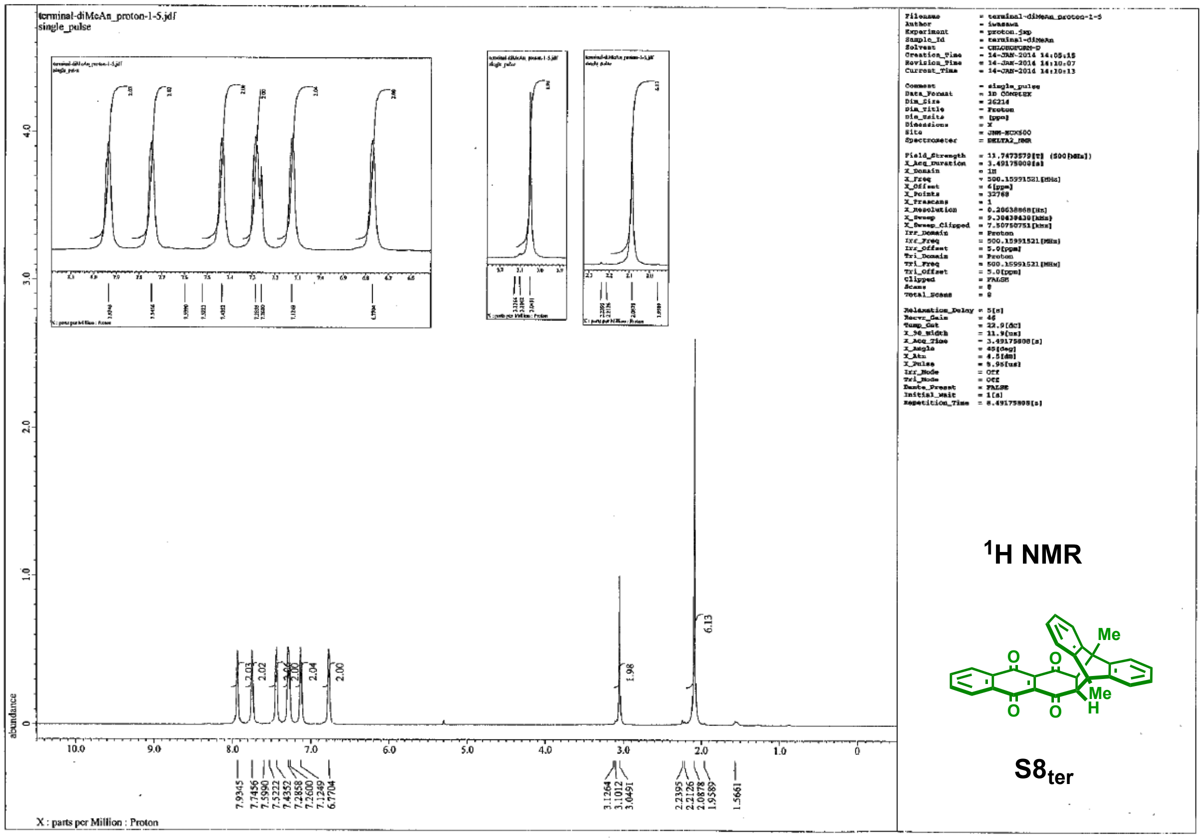
terminal-diMeAn_proton-1-5.jdf
single_pulse
X : parts per Million : Proton
abundance
1H NMR
Me
Me
H
O
O
O
O

S8ter

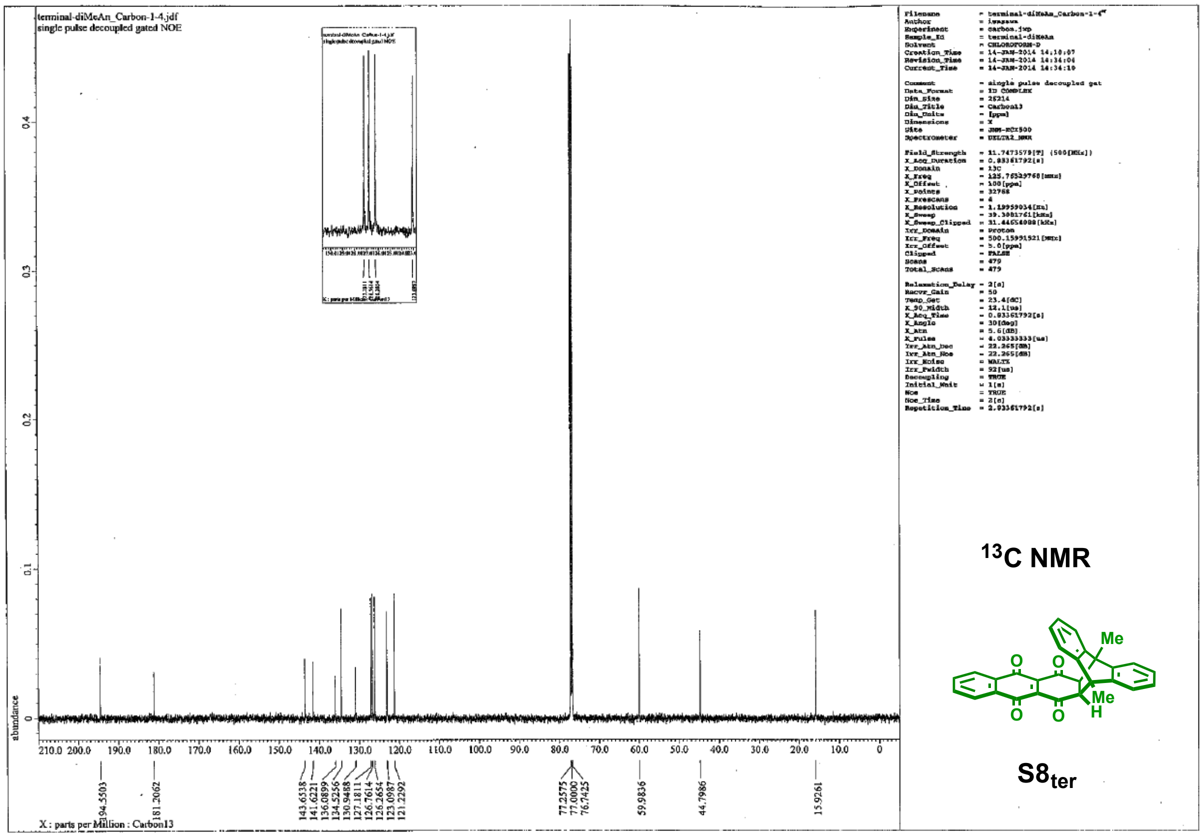
terminal-diMeAn_Carbon-1-4.jdf
single pulse decoupled gated NOE
X : parts per Million : Carbon13
abundance
13C NMR
Me
Me
H
O
O
O
O

S8ter

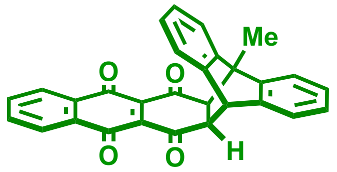

Physical data of **S9**$_{\mathbf{ter}}$

^{1}H NMR (500 MHz, $CDCl_3$): δ 7.95-7.92 (m, 2H), 7.77-7.74 (m, 2H), 7.41-7.44 (m, 2H), 7.28-7.23 (m, 2H), 7.17-7.15 (m, 1H), 7.13-7.12 (m, 1H), 6.79-6.77 (m, 2H), 4.73 (d, J = 2.9 Hz, 1H), 3.38 (dd, J = 8.8 Hz, 1H), 2.97 (d, J = 8.8 Hz, 1H), 2.09 (s, 3H).

^{13}C NMR (125 MHz, $CDCl_3$): δ 195.1, 194.9, 181.7, 181.2, 143.3, 141.3, 140.8, 139.3, 136.6, 134.7, 134.5, 134.1, 131.0, 130.9, 127.4, 127.4, 127.0, 126.9, 126.4, 126.2, 125.7, 124.0, 123.4, 121.4, 58.3, 54.8, 48.5, 45.7, 15.5.

HRMS (FAB^{+}, NBA): m/z Calcd. for $C_{29}H_{19}O_4$: 438.1284, Found: 431.1298 [M+H]$^{+}$.

IR (ATR): 1705, 1660, 1591, 1455, 1387, 1286, 1272, 1173 cm^{-1}.

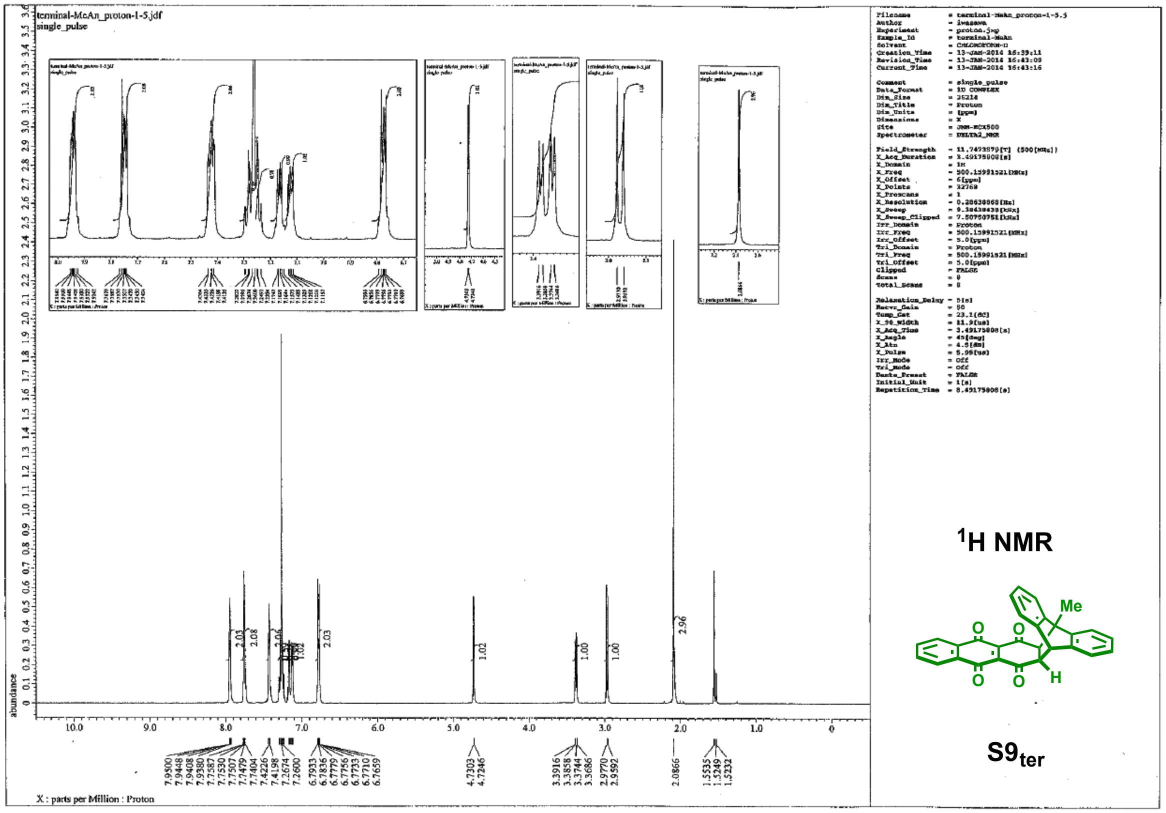

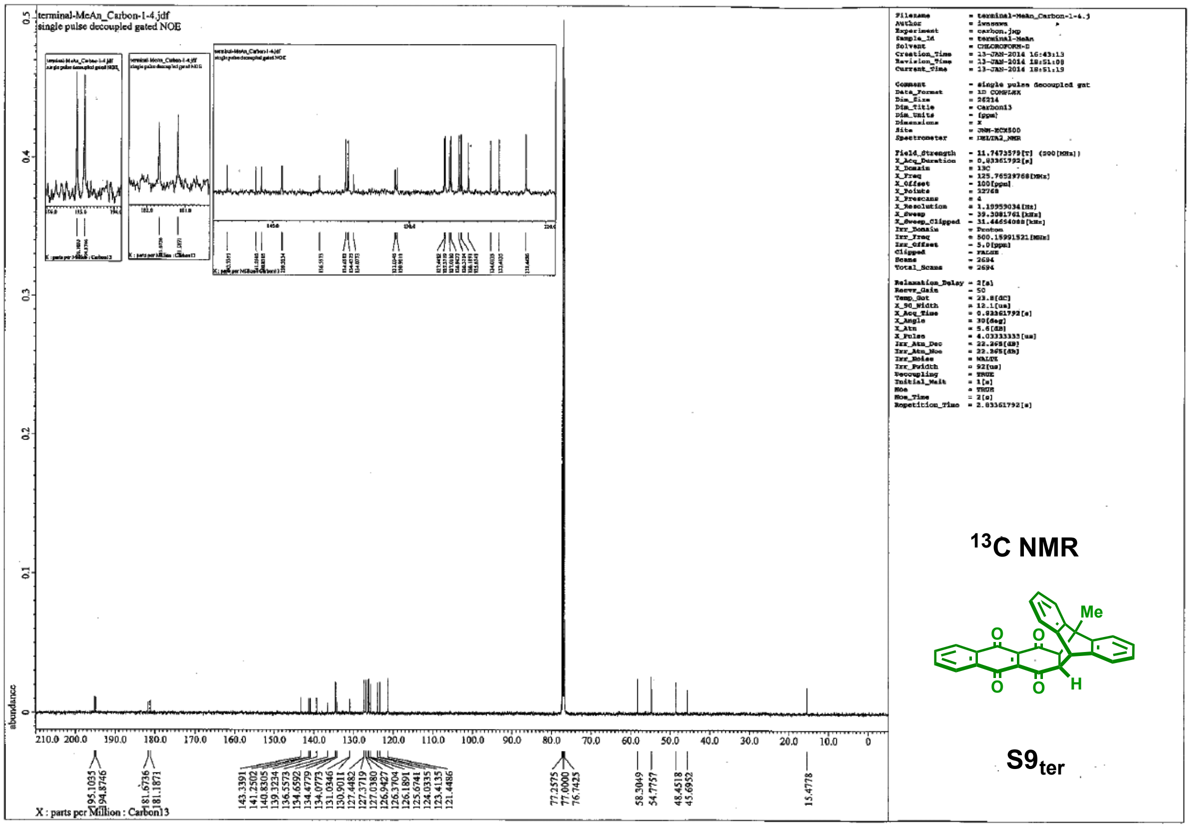

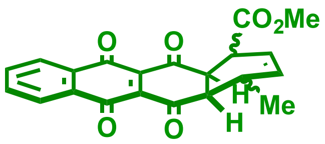

Physical data of **S10**$_{\mathbf{ter}}$

^{1}H NMR (500 MHz, $CDCl_3$): δ 8.09-8.05 (br, 2H), 7.84-7.80 (br, 2H), 6.18-6.15 (m, 1H), 5.71-5.75 (m, 1H), 4.10-4.09 (m, 1H), 3.75 (s, 3H), 3.63 (t, J = 6.9 Hz, 1H), 3.34-3.31 (m, 1H), 2.82-2.77 (m, 1H), 0.95 (d, J = 7.5 Hz, 3H).

^{13}C NMR (125 MHz, $CDCl_3$): δ 196.8, 194.6, 182.7, 136.2, 134.9, 134.8, 133.9, 132.0, 131.7, 130.8, 126.5, 121.5, 52.4, 52.1, 49.1, 40.0, 31.0, 17.7.

HRMS (FAB$^+$, NBA): m/z Calcd. for $C_{21}H_{18}O_6$: 336.1103, Found: 336.1125 $[M+2H]^+$. The peak derived from reduction product was observed in MS analysis.

IR (ATR): 1710, 1668, 1591, 1435, 1277, 1191, 908 cm^{-1}.

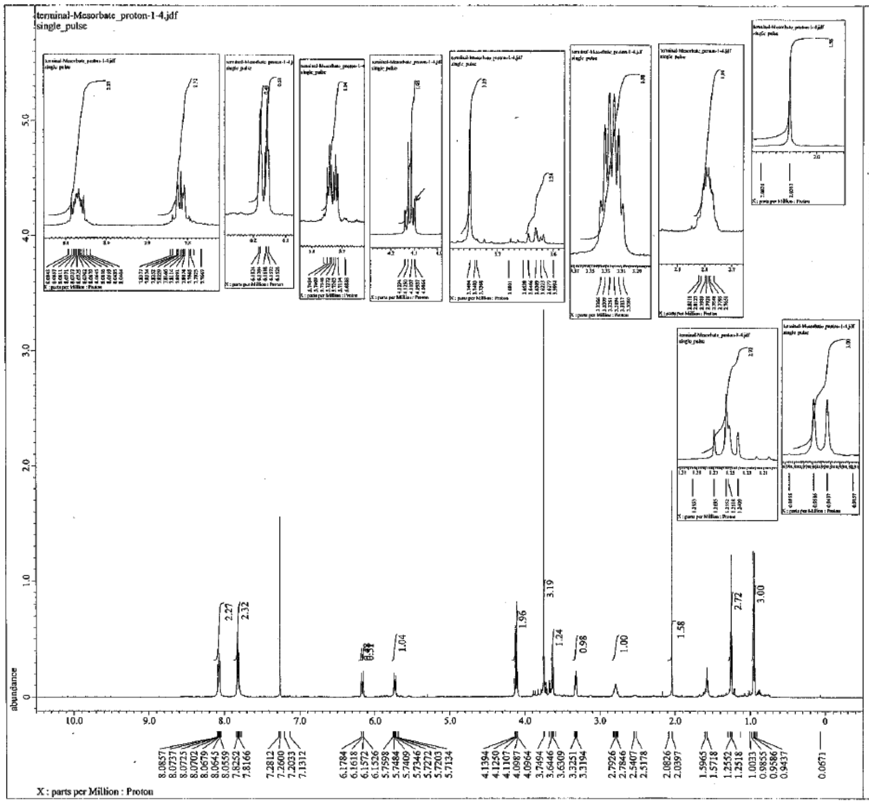

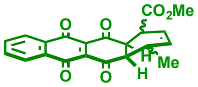

^{1}H NMR

$S10_{ter}$

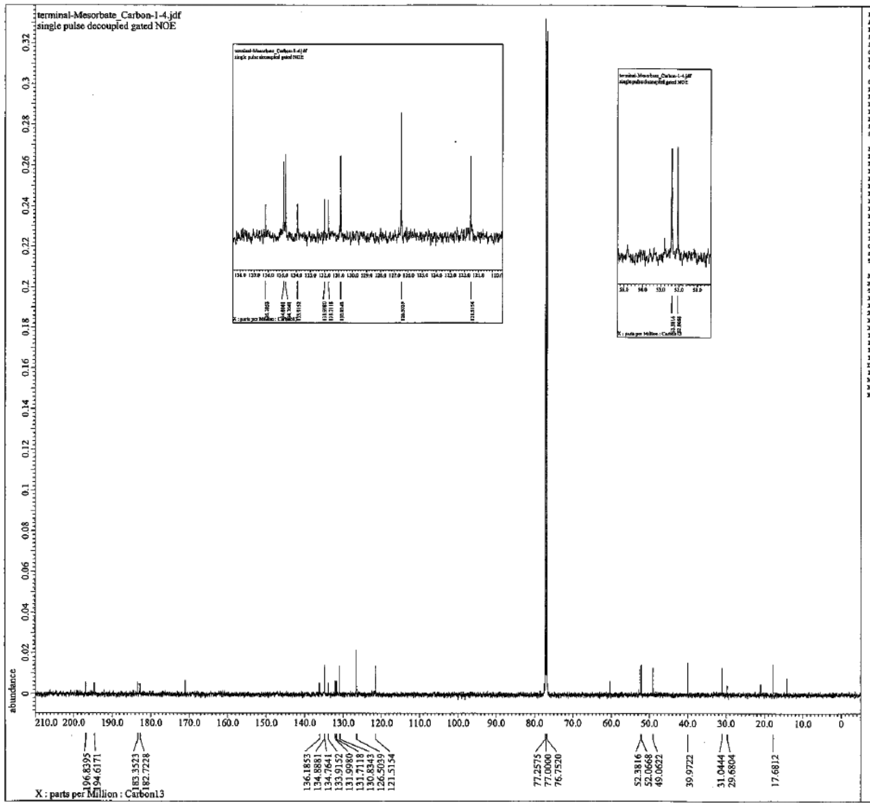

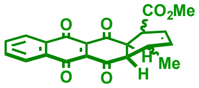

^{13}C NMR

$S10_{ter}$

5. Experimental Procedure and Characterization of New Compounds

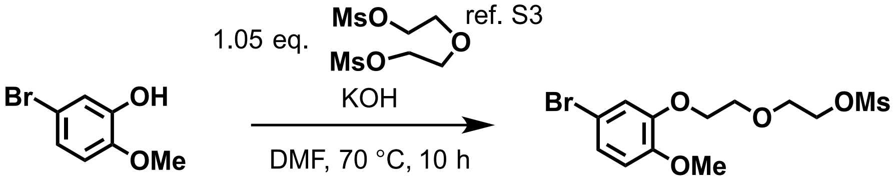

S11

To a DMF (100 mL) solution of 5-Bromo-2-methoxyphenol (5.42 g, 26.7 mmol) was added KOH (1.57 g, 27.9 mmol) and the mixture was heated at 70 ℃ for 1 h. After cooling at room temperature, to this solution was added a DMF (60 ml) solution of bis-[2-(methlsulfonyl)oxyethyl]ether (10.5 g, 40.1 mmol). After the mixture was heated at 70 ºC for 10 h, the reaction mixture was quenched with water. The organic materials were extracted with ethyl acetate three times, and the combined extracts were washed with brine, and dried over $MgSO_4$. After removal of the solvent under reduced pressure, the residue was purified by silica gel column chromatography (*n*-hexane/ethyl acetate = 1:1), and white solid of **S11** was obtained (4.77 g, 48% yield).

Physical data of **S11**

^{1}H NMR (500 MHz, $CDCl_3$): δ 7.05 (dd, J = 8.5, 2.1 Hz, 1H), 7.01 (d, J = 2.1 Hz, 1H), 6.74 (d, J = 8.5 Hz, 1H), 4.39-4.41 (m, 2H), 4.14-4.16 (m, 2H), 3.88-3.90 (m, 2H), 3.83-3.85 (m, 2H), 3,82 (s, 3H), 3.06 (s, 3H).

^{13}C NMR (125 MHz, $CDCl_3$): δ 148.8, 148.8, 124.2, 117.0, 113.0, 112.5, 69.6, 69.2, 68.7, 56.0, 37.7.

HRMS (FAB$^+$, NBA): *m/z* Calcd. for $C_{12}H_{17}O_6BrS$: 367.9929., Found: 367.9420 $[M]^+$.

IR (ATR): 1586, 1507, 1350, 1254, 1224, 1173, 1129, 1023, 1016, 959, 812 cm^{-1}.

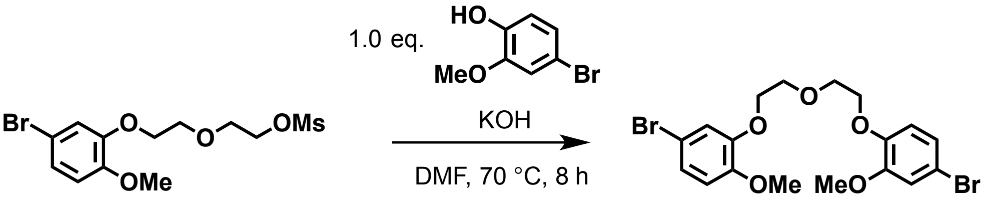

S11 **S12**

To a DMF (33 mL) solution of 4-Bromo-2-methoxyphenol (2.71 g, 12.7 mmol) was added KOH (712 mg, 12.7 mmol) and the mixture was heated at 70 ºC for 1 h. After cooling at room temperature, to this solution was added a DMF (37 ml) solution of **S11** (4.71g, 12.7 mmol). After the mixture was heated at 70 ºC for 8 h, the reaction mixture was quenched with water. The organic materials were extracted with ethyl acetate three times, and the combined extracts were washed with brine, and dried over $MgSO_4$. After removal of the solvent under reduced pressure, the residue was purified by silica gel column chromatography (*n*-

hexane/ethyl acetate = 2:1), and white solid of **S12** was obtained (4.83 g, 81% yield).

Physical data of **S12**

^{1}H NMR (500 MHz, $CDCl_3$): δ 7.04-7.01 (m, 2H), 6.99-6.96 (m, 2H), 6.78 (d, *J* = 8.3 Hz, 1H), 6.72-6.71 (m, 1H), 4.17-4.15 (m, 4H), 3.94-3.91 (m, 4H), 3.82 (s, 3H), 3,80 (s, 3H).

^{13}C NMR (125 MHz, $CDCl_3$): δ 150.4, 149.0, 148.9, 147.5, 123.9, 123.3, 117.0, 115.2, 115.2, 113.4, 113.0, 112.5, 69.8, 69.7, 68.9, 68.8, 56.1, 56.0.

HRMS (FAB$^+$, NBA): *m/z* Calcd. for $C_{19}H_{20}O_5Br_2$: 473.9677., Found: 473.9672 $[M]^+$.

IR (ATR): 1588, 1505, 1399, 1358, 1325, 1253, 1227, 1184, 1123, 1020, 965, 853, 841 cm^{-1}.

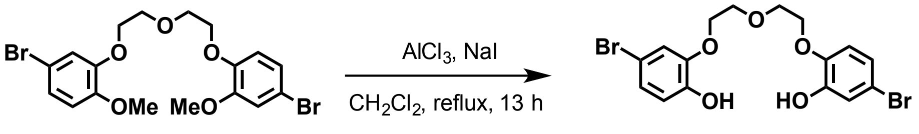

S12 **S13**

To a CH_2Cl_2 (100 mL) solution of **S12** (2 g, 4.2 mmol) was added $AlCl_3$ (3.36 g, 25.2 mmol) and NaI (6.29 g, 42 mmol). After the mixture was refluxed for 13 h, the reaction mixture was quenched with water. The organic materials were extracted with ethyl acetate three times, and the combined extracts were washed with brine, and dried over $MgSO_4$. After removal of the solvent under reduced pressure, the residue was purified by silica gel column chromatography (*n*-hexane/ethyl acetate = 3:2), and colorless oil of **S13** was obtained (1.56 g, 83% yield).

Physical data of **S13**

^{1}H NMR (500 MHz, $CDCl_3$): δ 7.13 (d, *J* = 2.6 Hz, 1H), 7.07-7.04 (m, 2H), 6.94 (dd, *J* = 8.6, 2.6 Hz, 1H), 6.86 (d, *J* = 8.6 Hz, 1H), 6.79 (d, *J* = 8.6 Hz, 1H), 4.19-4.15 (m, 4H), 3.87-3.83 (m, 4H).

^{13}C NMR (125 MHz, $CDCl_3$): δ 148.5, 146.7, 146.4, 145.1, 126.2, 123.0, 119.5, 119.1, 117.8, 117.5, 115.7, 111.2, 70.3, 69.9, 69.4, 69.4.

HRMS (FAB$^+$, NBA): *m/z* Calcd. for $C_{16}H_{16}O_5Br_2$: 445.9344., Found: 445.9335 $[M]^+$.

IR (ATR): 3392, 1490, 1455, 1362, 1259, 1216, 1115, 1050, 941, 870, 792 cm^{-1}.

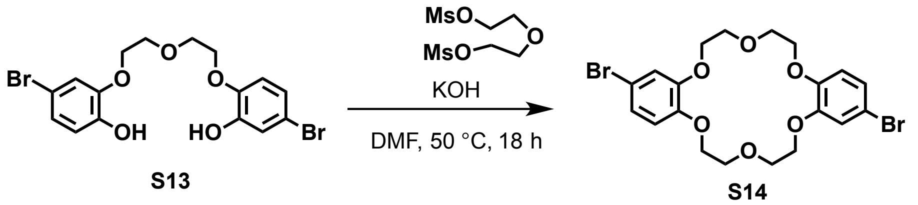

S13 **S14**

To a DMF (70 mL) solution of **S13** (1.5 g, 3.34 mmol) was added KOH (0.39 g, 3.51 mmol) and the mixture was heated at 70 ºC for 1 h. After cooling at room temperature, to this solution was added a DMF (60 ml) solution of bis[2-(methlsulfonyl)oxyethyl]ether (0.92 g, 3.51 mmol). After the mixture was heated at 70 ℃

for 10 h, the reaction mixture was quenched with water. The precipitate was filtered and washed with water and ethyl acetate, and dried to afford the white solid **S14** (1.28 g, 74% yield).

Physical data of **S14**

^{1}H NMR (500 MHz, $CDCl_3$): δ 7.00 (dd, *J* = 6.7, 2.3 Hz, 1H), 6.97 (d, *J* = 2.3 Hz, 1H), 6.73 (d, *J* = 6.7 Hz, 2H), 4.14-4.12 (m, 8H), 4.00-3.99 (m, 8H).

^{13}C NMR (125 MHz, $CDCl_3$): δ 149.5, 148.0, 123.8, 116.8, 114.9, 113.1, 69.9, 69.7, 69.0.

HRMS (FAB^+, NBA): *m/z* Calcd. for $C_{20}H_{22}O_6Br_2$: 513.9763., Found: 513.9756 $[M]^+$.

IR (ATR): 1506, 1252, 1227, 1134, 997, 931, 864 cm^{-1}.

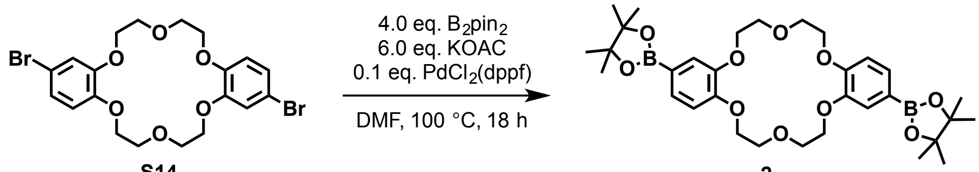

S14 **2pin**

To a DMF (27 mL) solution of **S14** (1.24 g, 2.39 mmol) was added $PdCl_2$(dppf) (174 mg, 0.239 mmol), B_2pin_2(2.42 g, 9.57 mmol) and KOAc (1.40 g, 14.34 mmol). After the mixture was heated at 100 ºC for 18 h, the reaction mixture was quenched with water. The organic materials were extracted with CH_2Cl_2 three times, and the combined extracts were washed with water five times and brine, and dried over $MgSO_4$. After removal of the solvent under reduced pressure, the residue was purified by GPC to afford the desired product $\mathbf{2_{pin}}$ (658 mg, 45% yield).

Physical data of $\mathbf{2_{pin}}$

^{1}H NMR (400 MHz, $CDCl_3$): δ 7.38 (dd, *J* = 8.1, 1.2 Hz, 2H), 7.27 (d, *J* = 1.2 Hz, 2H), 6.86 (d, *J* = 8.1 Hz, 2H), 4.23-4.21 (m, 2H), 4.17 (m, 2H), 4.06-4.04 (m, 2H), 4.02-4.00 (m, 2H), 1.32 (s, 24H).

^{13}C NMR (125 MHz, $CDCl_3$): δ 151.5, 148.1, 128.9, 121.3, 119.2, 112.6, 83.6, 70.0, 69.9, 69.0, 68.5, 24.9.

HRMS (FAB^+, NBA): *m/z* Calcd. for $C_{32}H_{46}O_{10}Br_2$: 612.3277., Found: 612.3274 $[M]^+$.

IR (ATR): 1601, 1520, 1419, 1353, 1300, 1258, 1217, 1138, 1060, 970 cm^{-1}.

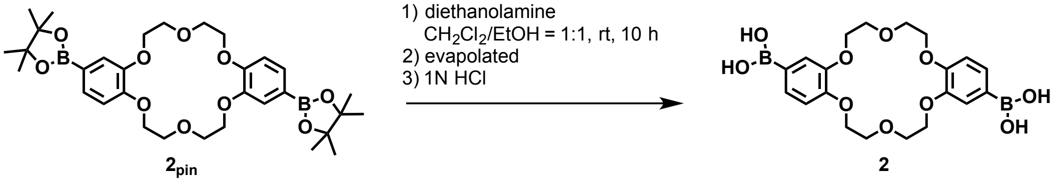

$\mathbf{2_{pin}}$ **2**

To a CH_2Cl_2/EtOH (9:1) (35 mL) solution of $\mathbf{2_{pin}}$ (350 mg, 0.57 mmol) was added diethanolamine (0.35 mL, 3.42 mmol). After stirred at room temperature for 10 h, solvent was removed under reduced pressure. 1N HCl (10 mL) were added to the residue and stirred for 2 h, white precipitate was obtained by filtration. The precipitate was washed with water and CH_2Cl_2, **2** (185 mg, 72% yield) was obtained.

Physical data of **2**

^{1}H NMR (500 MHz, DMSO-d_6): δ 7.85 (s, 4H), 7.36-7.35 (m, 4H), 6.90 (d, J = 8.3 Hz, 2H), 4.07 (br, 8H), 3.84 (br, 8H).

^{13}C NMR (125 MHz, DMSO-d_6): δ 149.7, 147.0, 127.6, 125.7, 117.6, 111.4, 69.0, 68.9, 67.5, 67.4.

IR (ATR): 3363, 1601, 1519, 1420, 1388, 1349, 1315, 1257, 1227, 1157, 1121, 1087, 1052, 988, 942 cm^{-1}.

6. ^{1}H and ^{13}C NMR Spectra of New Compounds

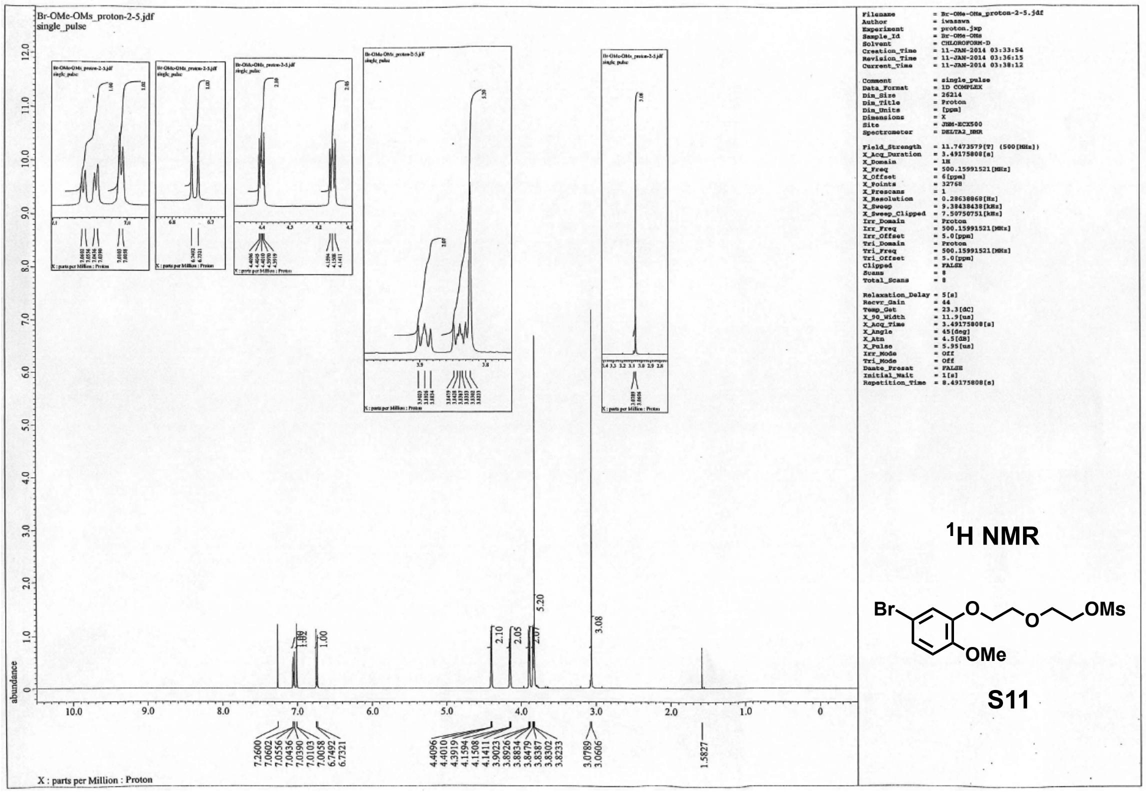

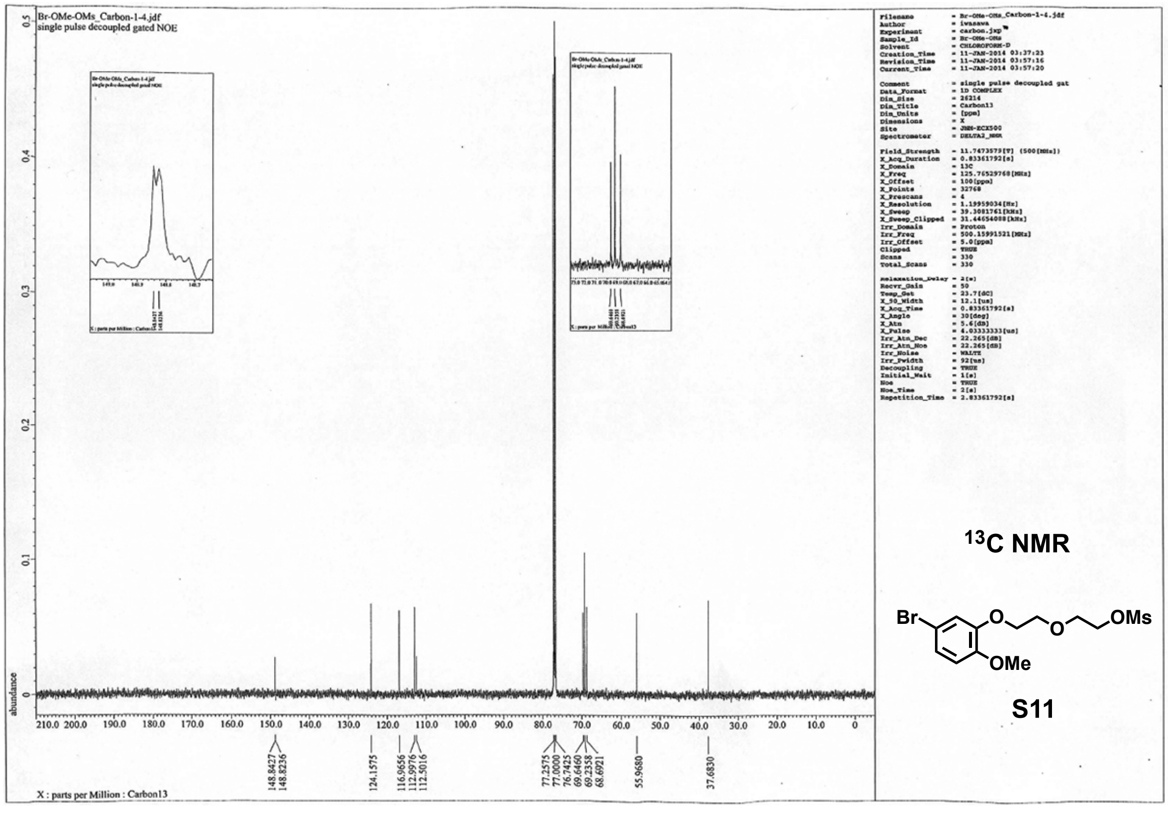

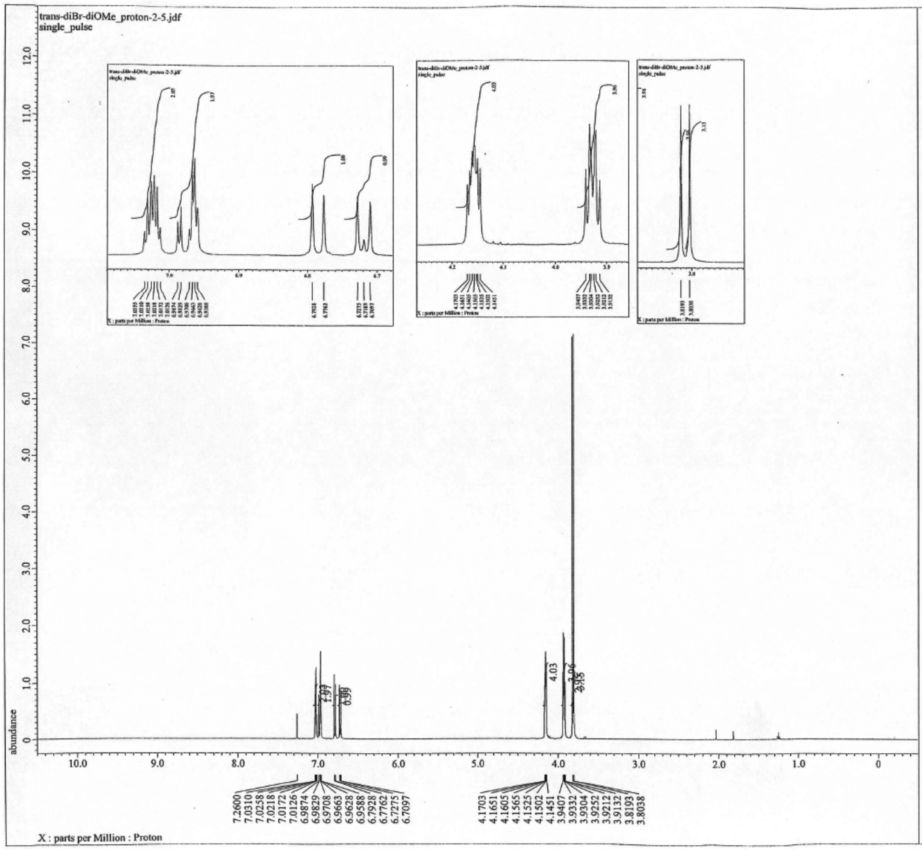

^{1}H NMR

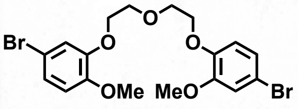

S12

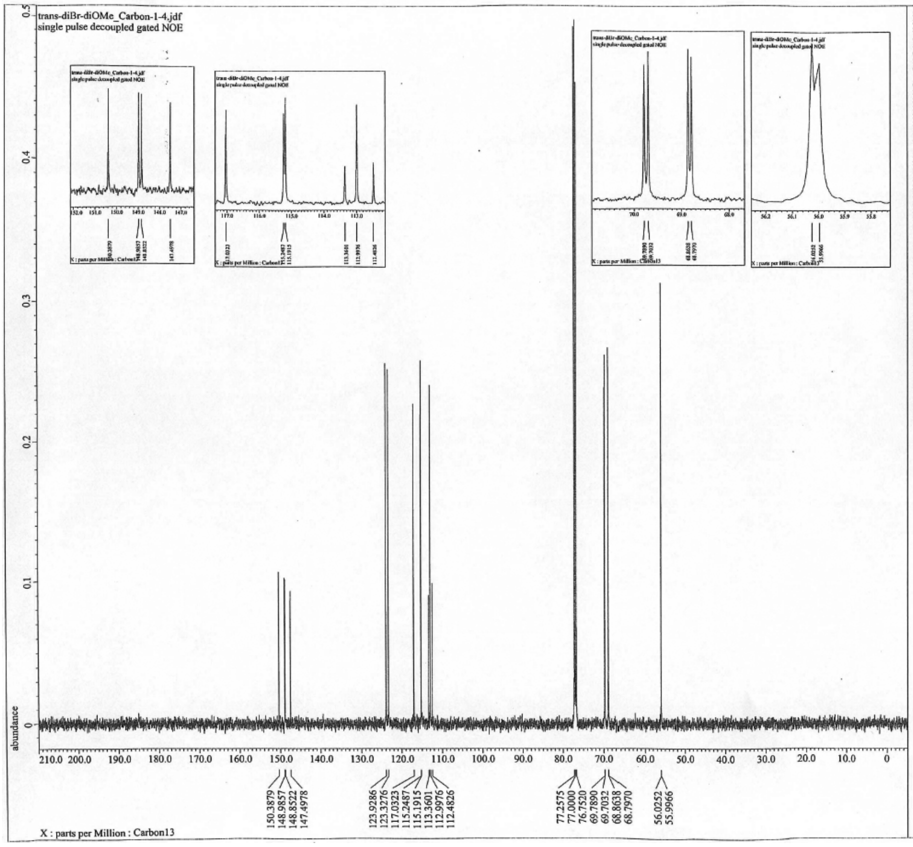

^{13}C NMR

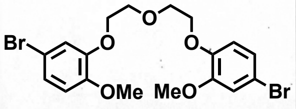

S12

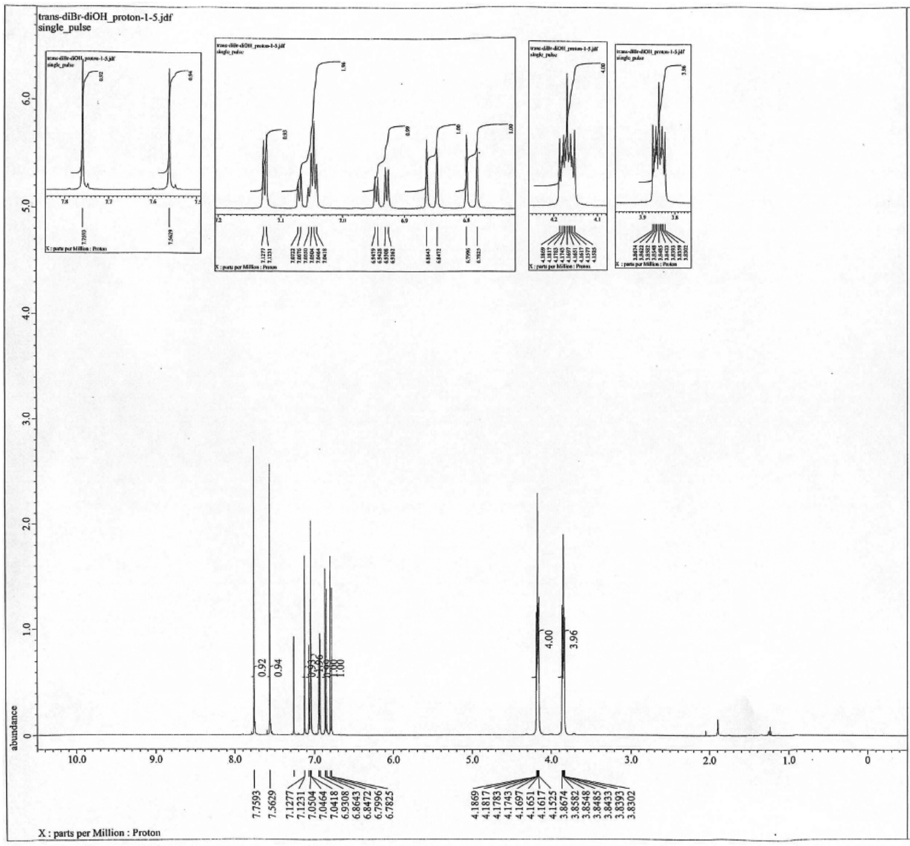

^{1}H NMR

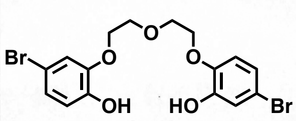

S13

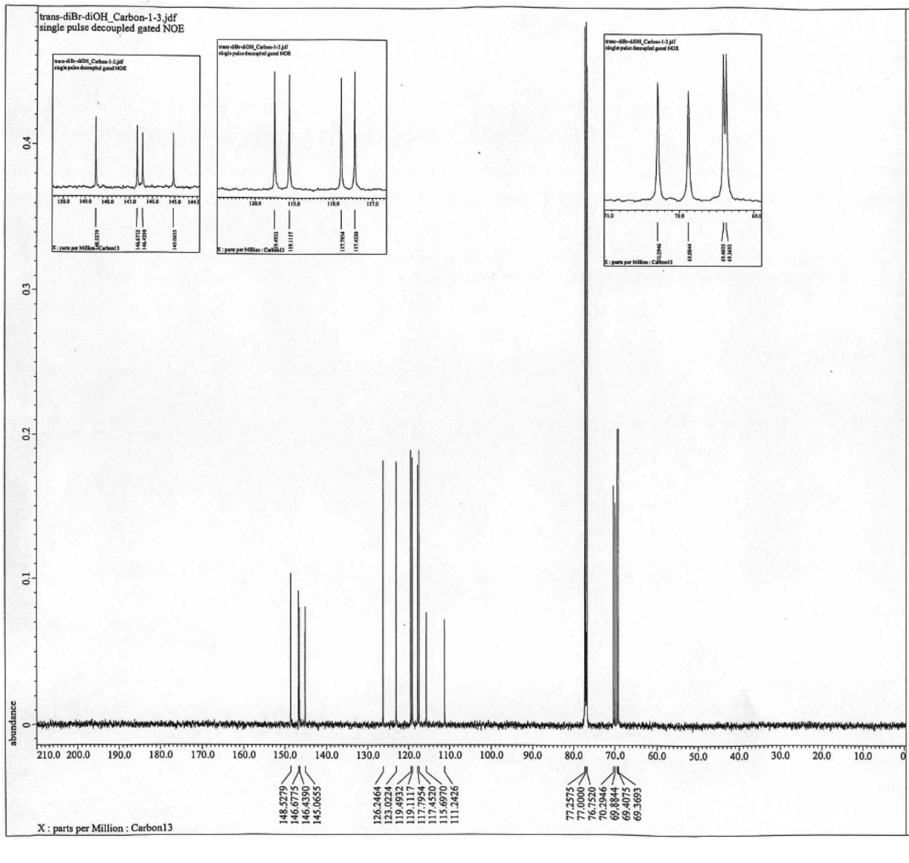

^{13}C NMR

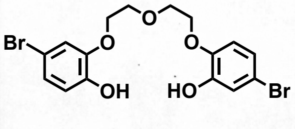

S13

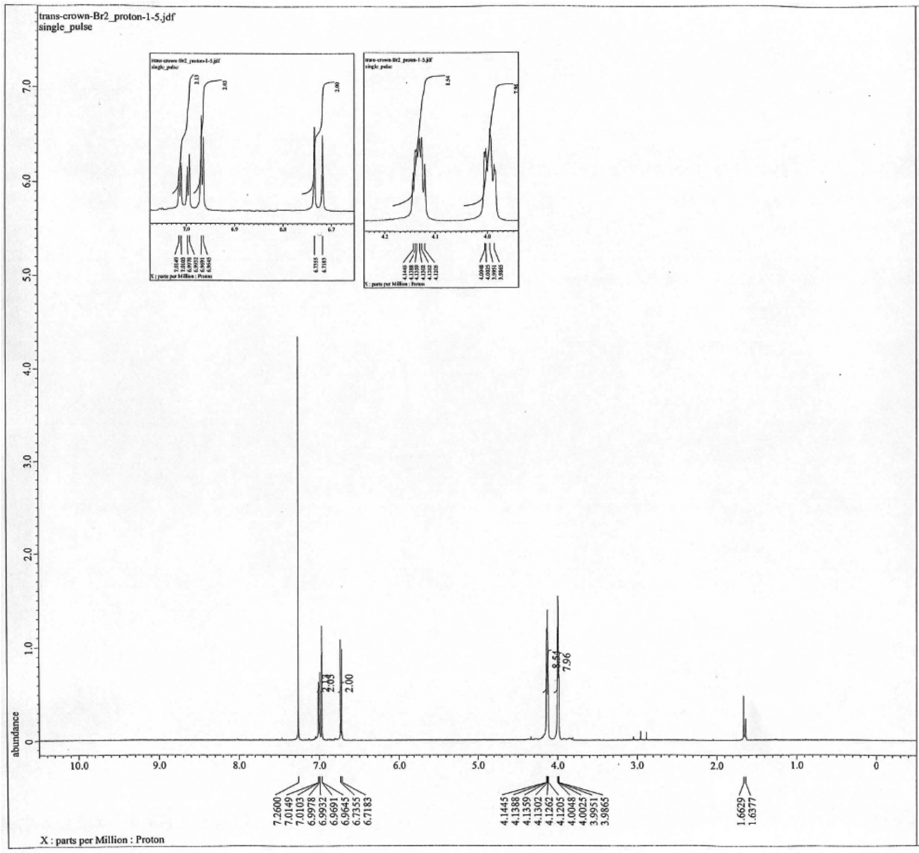

^{1}H NMR

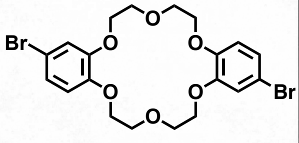

S14

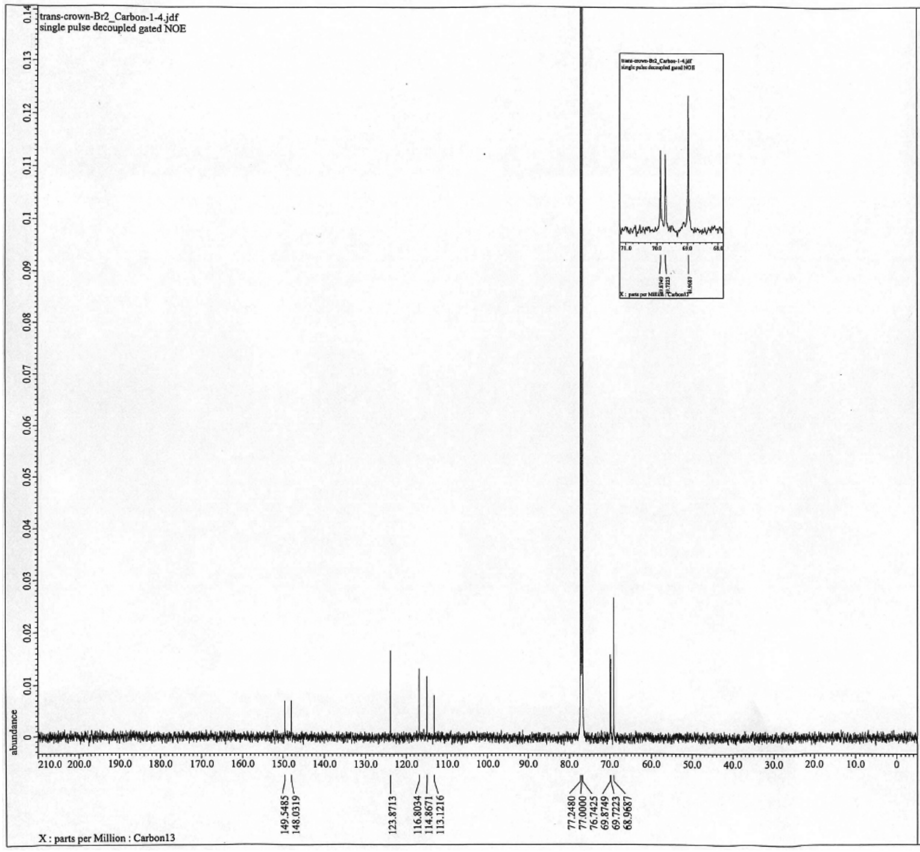

^{13}C NMR

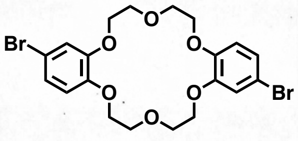

S14

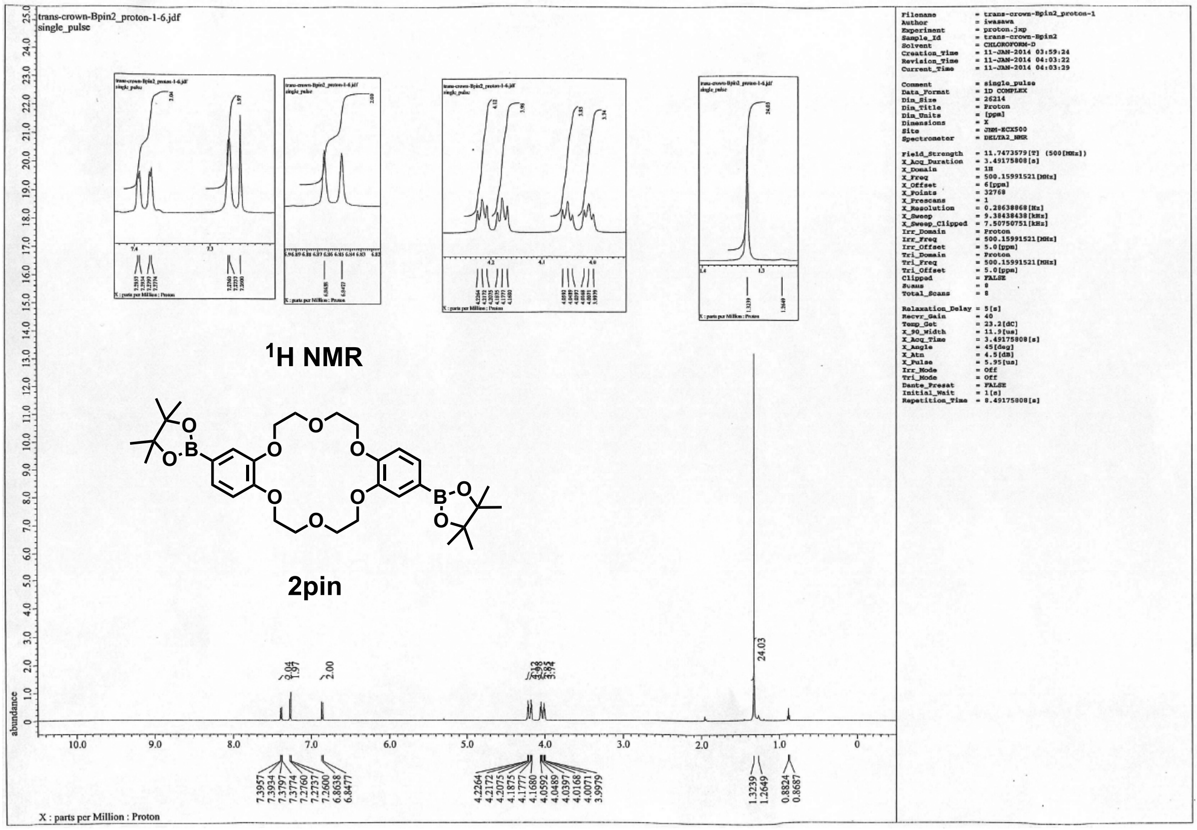
trans-crown-Bpin2_proton-1-6.jdf
single_pulse
1H NMR
2pin
2.04
1.97
2.00
24.03
7.3957
7.3934
7.3797
7.3774
7.2760
7.2737
7.2600
6.8638
6.8477
4.2264
4.2172
4.2075
4.1877
4.1775
4.1680
4.0592
4.0489
4.0397
4.0168
4.0071
3.9979
1.3239
1.2649
0.8824
0.8687
X : parts per Million : Proton

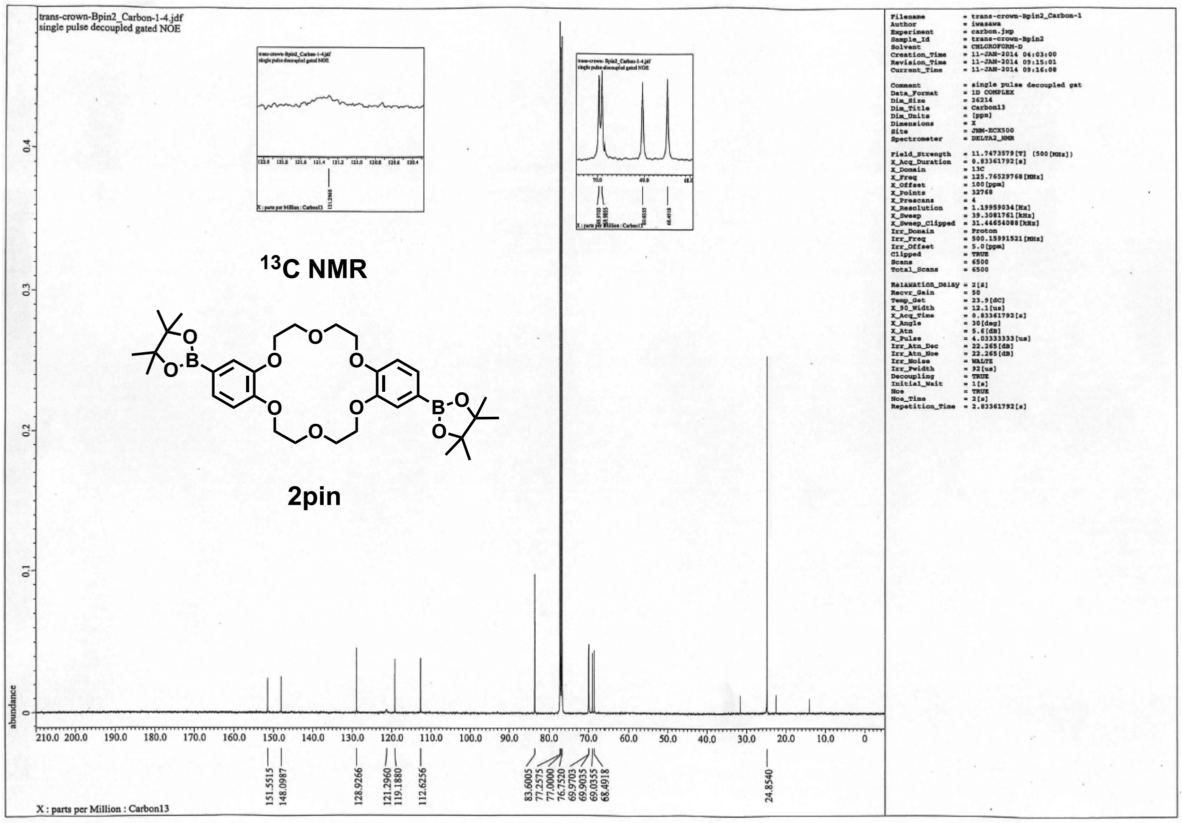
trans-crown-Bpin2_Carbon-1-4.jdf
single pulse decoupled gated NOE
13C NMR
2pin
151.5515
148.0987
128.9266
121.2960
119.1880
112.6256
83.6005
77.2575
77.0000
76.7520
69.9703
69.9035
69.0355
68.4918
24.8540
X : parts per Million : Carbon13

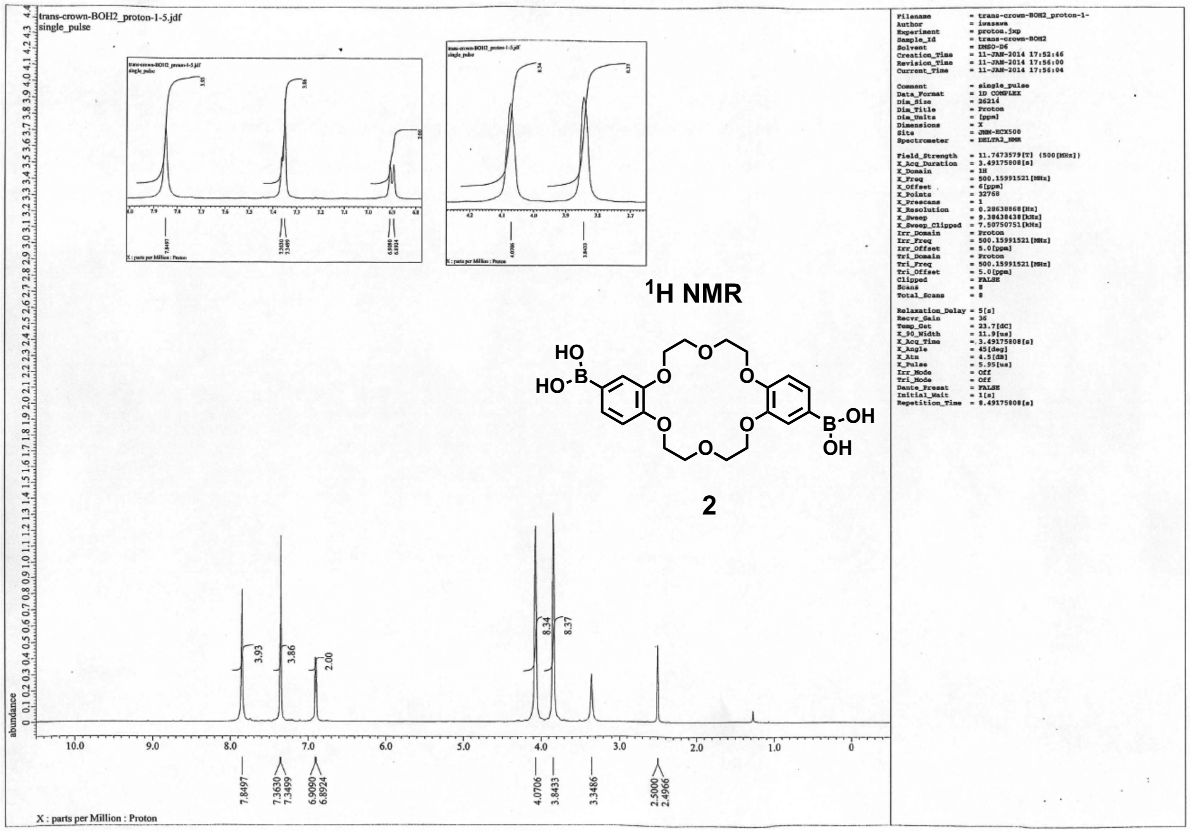
trans-crown-BOH2_proton-1-5.jdf
single_pulse
1H NMR
2
3.93
3.86
2.00
8.34
8.37
7.8497
7.3630
7.3499
6.9090
6.8924
4.0706
3.8433
3.3486
2.5000
2.4966
X : parts per Million : Proton

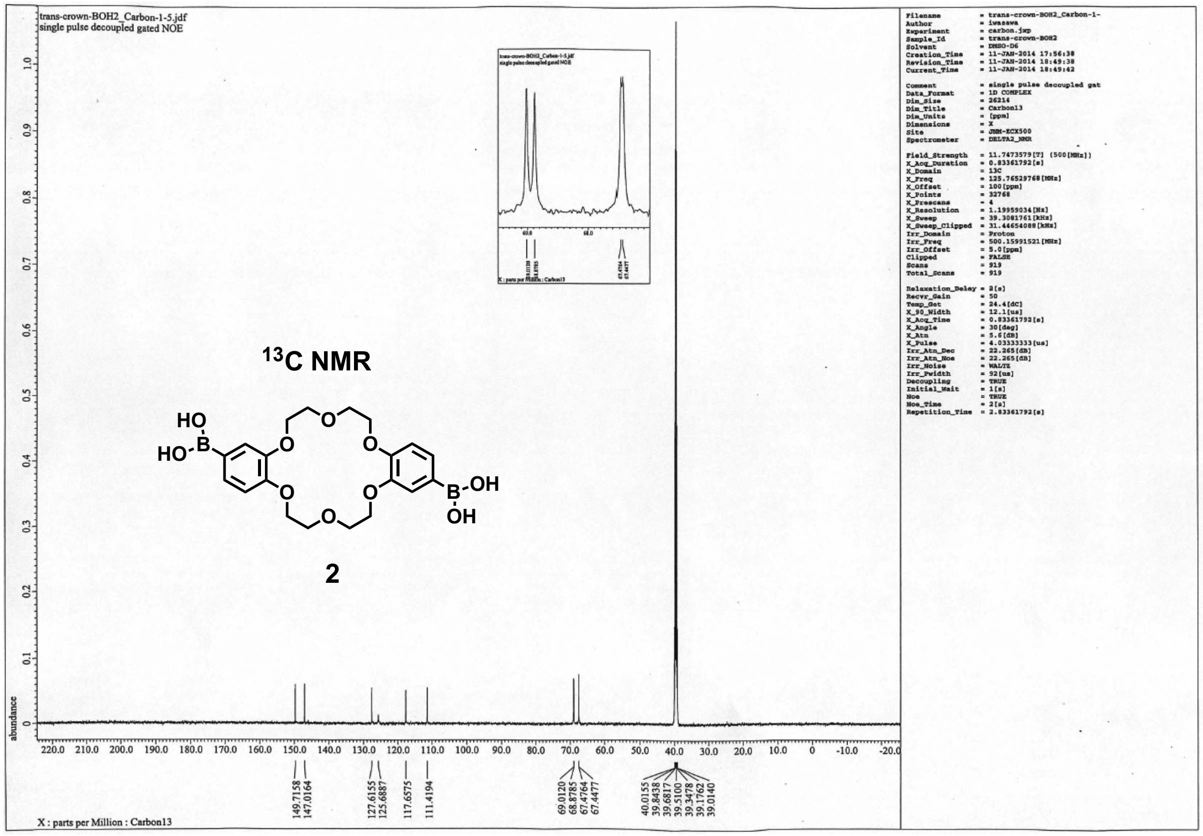
trans-crown-BOH2_Carbon-1-5.jdf
single pulse decoupled gated NOE
13C NMR
2
149.7158
147.0164
127.6155
125.6887
117.6575
111.4194
69.0120
68.8785
67.4764
67.4477
40.0155
39.8438
39.6817
39.5100
39.3478
39.1762
39.0140
X : parts per Million : Carbon13

7. X-ray Crystallographic Analysis of a racemic crystal of [2+2]$_{crown}$

Single crystals, suitable for X-ray diffraction analysis, were obtained as racemic crystals from the mixture of equimolar amounts of macrocyclic boronic esters constructed by using **2** and (+)-tetrol or (–)-tetrol, respectively. Racemic crystals of **[2+2]$_{crown}$** were obtained by vapor diffusion method using a dichloromethane solution as a good solvent, and pentane as a poor solvent.

The single crystal X-ray diffraction data were collected on a Rigaku R-AXIS II with IP area detector using Cu*K*α (λ = 1.54186 Å). The structure was solved by direct methods by *SHELXS*. The cage molecular structure of **[2+2]$_{crown}$** was obtained. The five dichloromethane molecules were included by **[2+2]$_{crown}$** (the site occupancy factors were 0,8, 0.8, 0.7, 0.4 and 0.65, respectively). And a dichloromethane molecule was outside the cage (the site occupancy factor is 0.4). The refinement was performed by full-matrix least squares using *SHELXL*-2014. Crystal structure of **[2+2]$_{crown}$**: C_{72} H_{80} B_4 O_{20}, 3.75(C H_2 Cl_2) M_r = 1627.07, Monoclinic, $P2_1/c$, a = 18.3378(10) Å, b = 20.9587(10) Å, c = 22.0613(12) Å, β = 91.6688(18)° , V=8475.4(8) Å^3, D_{calc} = 1.275 g cm^{-3}, T = 93(2) K, no. of unique reflections = 15473, R_{int} = 0.1521, no. of parameters = 1042, no. of restraints = 60, R_1 = 0.1203, wR_2 = 0.3170, S =0.893 for 15473 reflections, max/min. residual density 0.757/-0.264 eÅ^{-3}. CCDC reference number 1541693.

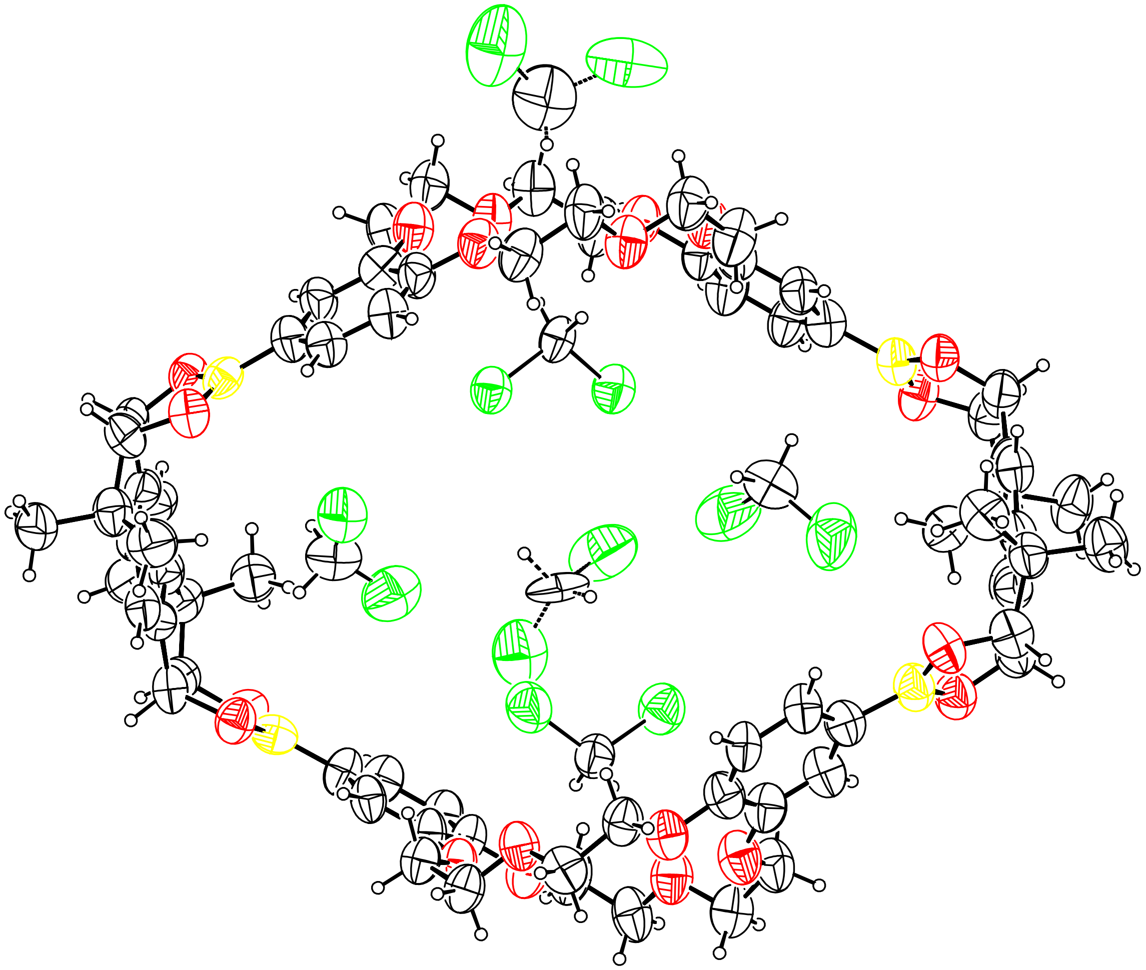

Fig. S29 ORTEP drawing (30% probability ellipsoids) of **[2+2]$_{\mathbf{crown}}$**.

Table S3 Crystal data and structure refinement for **[2+2]$_{\mathbf{crown}}$**.

Empirical formula	$C_{72} H_{80} B_4 O_{20}$, 3.75(CH_2Cl_2)
Formula weight	1627.07
Temperature	93(2) K
Wavelength	1.54186 Å
Crystal system	Monoclinic
Space group	$P\ 2_1/c$
Unit cell dimensions	a = 18.3378(10) Å.
	b = 20.9587(10) Å β= 91.6688(18)°.
	c = 22.0613(12) Å.
Volume	8475.4(8) $Å^3$
Z	4
Density (calculated)	1.275 Mg/m^3
Absorption coefficient	2.828 mm^{-1}
F(000)	3398
Crystal size	0.150 x 0.110 x 0.090 mm^3
Theta range for data collection	3.203 to 68.247°.
Index ranges	$-22<=h<=22$, $-25<=k<=24$, $-26<=l<=26$
Reflections collected	78205
Independent reflections	15473 [R(int) = 0.1521]
Completeness to theta = 67.686°	99.9 %
Absorption correction	Semi-empirical from equivalents
Max. and min. transmission	0.785 and 0.482
Refinement method	Full-matrix least-squares on F^2
Data / restraints / parameters	15473 / 60 / 1042
Goodness-of-fit on F^2	0.893
Final R indices [I>2sigma(I)]	R_1 = 0.1203, wR_2 = 0.3170
R indices (all data)	R_1 = 0.2748, wR_2 = 0.4130
Extinction coefficient	n/a
Largest diff. peak and hole	0.757 and -0.264 e.$Å^{-3}$

8. References

S1) S. J. Bingham and J. H. P. Tyman, *J. Chem. Soc.*, *Perkin Trans. 1,* 1997, 3637–3642.

S2) (a) H. Sakurai, N. Iwasawa and K. Narasaka, *Bull. Chem. Soc. Jpn.,* 1996, **69**, 2585–2594; (b) S. Ito, K. Ono and N. Iwasawa, *J. Am. Chem. Soc.,* 2012, **134**, 13962–13965.

S3) A. M. Ingham, C. Xu, T. W. Whitcombe, C. Xu, J. N. Bridson and A. McAuley, *Can. J. Chem*., 2002, **80**, 155–162.

S4) S. Sen, S. Singh and S. M. Sieburth, *J. Org. Chem.,* 2009, **74**, 2884–2886.
